# The relationship between type, timing and duration of exposure to adverse childhood experiences and adolescent self-harm and depression: findings from three UK prospective population-based cohorts

**DOI:** 10.1111/jcpp.13986

**Published:** 2024-04-13

**Authors:** Bushra Farooq, Abigail E. Russell, Laura D. Howe, Annie Herbert, Andrew D.A.C. Smith, Helen L. Fisher, Jessie R. Baldwin, Louise Arseneault, Andrea Danese, Becky Mars

**Affiliations:** 1 Centre for Academic Mental Health, Population Health Sciences, University of Bristol Medical School, Bristol, UK; 2 Children and Young People’s Mental Health Research Collaboration, University of Exeter Medical School, Exeter, UK; 3 MRC Integrative Epidemiology Unit, Population Health Sciences, University of Bristol Medical School, Bristol, UK; 4 Mathematics and Statistics Research Group, University of the West of England, Bristol, UK; 5 Social, Genetic & Developmental Psychiatry Centre, Institute of Psychiatry, Psychology & Neuroscience, King’s College London, London, UK; 6 ESRC Centre for Society and Mental Health, King’s College London, London, UK; 7 Division of Psychology and Language Sciences, Department of Clinical, Educational and Health Psychology, University College London, London, UK; 8 Department of Child and Adolescent Psychiatry, Institute of Psychiatry, Psychology & Neuroscience, King’s College London, London, UK; 9 National and Specialist CAMHS Clinic for Trauma, Anxiety, and Depression, South London and Maudsley NHS Foundation Trust, London, UK; 10 National Institute for Health and Care Research, Biomedical Research Centre, Bristol, UK

**Keywords:** Adverse childhood experiences, self-harm, depression, ALSPAC, E-Risk, MCS, cohort, developmental timing, accumulation of risk.

## Abstract

**Background:**

Adverse childhood experiences (ACEs) are well-established risk factors for self-harm and depression. However, despite their high comorbidity, there has been little focus on the impact of developmental timing and the duration of exposure to ACEs on co-occurring self-harm and depression.

**Methods:**

Data were utilised from over 22,000 children and adolescents participating in three UK cohorts, followed up longitudinally for 14–18 years: the Avon Longitudinal Study of Parents and Children (ALSPAC), the Millennium Cohort Study (MCS) and the Environmental Risk (E-Risk) Longitudinal Twin Study. Multinomial logistic regression models estimated associations between each ACE type and a four-category outcome: no self-harm or depression, self-harm alone, depression alone and self-harm with co-occurring depression. A structured life course modelling approach was used to examine whether the accumulation (duration) of exposure to each ACE, or a critical period (timing of ACEs) had the strongest effects on self-harm and depression in adolescence.

**Results:**

The majority of ACEs were associated with co-occurring self-harm and depression, with consistent findings across cohorts. The importance of timing and duration of ACEs differed across ACEs and across cohorts. For parental mental health problems, longer duration of exposure was strongly associated with co-occurring self-harm and depression in both ALSPAC (adjusted OR: 1.18, 95% CI: 1.10–1.25) and MCS (1.18, 1.11–1.26) cohorts. For other ACEs in ALSPAC, exposure in middle childhood was most strongly associated with co-occurring self-harm and depression, and ACE occurrence in early childhood and adolescence was more important in the MCS.

**Conclusions:**

Efforts to mitigate the impact of ACEs should start in early life with continued support throughout childhood, to prevent long-term exposure to ACEs contributing to risk of self-harm and depression in adolescence.

## Introduction

Self-harm and depression are both major public health concerns and strong risk factors for suicide (Hawton, Casanas, Haw, & Saunders, 2013; Morgan et al., 2017). The co-occurrence of self-harm and depression is high in Western countries—with prevalence estimates of up to 60% for depression among those who self-harm (Hawton, Saunders, Topiwala, & Haw, 2013). In one population-based cohort study, among adolescents with major depressive disorder, 47% reported self-harm and 19.7% reported a suicide attempt over 12 months, in comparison, among those with no mental disorder, only 4.2% reported self-harm and 0.4% reported a suicide attempt (Lawrence et al., 2015). Similarly, self-harm has been shown to be independently associated with the presence of depression among adolescents (Moran et al., 2012). Examining their co-occurrence is important as it confers a greater risk of suicide—in one study of patients with depression, the presence of self-harm increased the risk of suicide by twofold (Aaltonen, Isometsa, Sund, & Pirkola, 2019). Another study found depression is more strongly associated with self-harm with suicidal intent (Mars et al., 2014). Due to their high co-occurrence and their heightened combined risk, it is important to identify early risk factors to target preventions efforts.

Adverse childhood experiences (ACEs) such as abuse, neglect and household dysfunction are common in the general population and are one of the strongest risk factors for self-harm and depression (Bellis, Hughes, Leckenby, Perkins, & Lowey, 2014; Hughes et al., 2017; Russell et al., 2021). ACEs rarely occur in isolation, experiencing one increases the risk of experiencing additional adversities (Brown, Rienks, McCrae, & Watamura, 2019; Dong et al., 2004). To account for this co-occurrence, the total number of adversities for each individual is conventionally summed to derive a total score—referred to as the ‘ACE score’, which is used as a predictor to investigate associations with different mental health outcomes, including self-harm and depression (Bomysoad & Francis, 2020; Houtepen et al., 2020; Russell et al., 2019). This approach neglects the potentially differing impact of individual adversities, as well as factors such as the duration of their exposure, and the life-course stage at which the adversity occurs, highlighting the importance of examining ACEs separately to better understand implications for prevention efforts (Negriff, 2020).

Life-course epidemiology theories may help understand how the timing and duration of ACE exposure may be associated with later health. Under the critical and sensitive period hypotheses, timing may be important because the exposure could potentially have differing effects if experienced at a vulnerable time period of rapid maturation in the brain (Knudsen, 2004; Kuh, Ben-Shlomo, Lynch, Hallqvist, & Power, 2003). An exposure occurring during a critical period causes irreversible changes with no scope for later modification of the effects on outcomes. However, with sensitive periods, there is a stronger association between an exposure and outcome during a specific period relative to other developmental periods (Kuh et al., 2003; Schaefer, Cheng, & Dunn, 2022). A review of the literature suggested that sensitive periods may differ according to ACE type (Schaefer et al., 2022). For some ACE types, evidence to support the accumulation hypothesis has emerged (Dunn et al., 2018). The accumulation of risk hypothesis suggests cumulative exposure over the life-course increases risk of poor outcomes (i.e. as the duration of exposure increases the risk of the outcome of interest increases) (Kuh et al., 2003). Accumulation of maternal depression has been linked to increased likelihood of depression in offspring, and longer duration of exposure to maltreatment has been linked to increased risk of self-harm (Hu, Taylor, Li, & Glauert, 2017; Lacey et al., 2023). However, previous studies have focused on a limited number of ACEs (Dunn, McLaughlin, Slopen, Rosand, & Smoller, 2013; Lacey et al., 2023). Some have examined shorter periods of exposure (e.g. between birth and 8 years) and short-term outcomes during childhood (Dunn, Soare, et al., 2018). As such, it is currently unclear whether risk of self-harm and depression is affected by the type, timing and duration of ACEs.

To address these gaps, we used data from over 22,000 children followed longitudinally for at least 14 years, and comprehensively assessed for 11 different ACEs, self-harm and depression. This allowed us to investigate (i) the individual association with each ACE and (ii) the relationship between timing and duration of exposure to ACEs at three developmental stages.

## Key definitions

### Adverse childhood experiences

We defined ACEs as negative exposures during childhood, we examined 10 ACEs from the World Health Organization ACE International Questionnaire and additionally, physical neglect in the E-Risk study (World Health Organization, 2020).

### Critical period

A critical period hypothesis suggests an exposure that occurs during a crucial developmental stage, impacts the structure or function of organs, tissues or body systems and cannot be modified. These effects are unaffected by subsequent experiences and contribute to the onset of disease in later life. Therefore, a critical period is a limited time window in which an exposure can have adverse effects on development and subsequent outcomes. However, outside this developmental window, there is no excess risk associated with exposure (Kuh et al., 2003).

### Sensitive period

Similar to critical periods, a sensitive period is a time period when an exposure has a stronger effect on an outcome than it would at other times, outside the time period any excess risk is weaker (Kuh et al., 2003).

### Accumulation of risk

In line with the accumulation of risk hypothesis from life-course epidemiology, we defined accumulation of exposure to each ACE as recurrent exposure to the same ACE across different developmental periods (early childhood, middle childhood and adolescence) (Mishra et al., 2009). This means that we focused on measuring the number of occasions or duration in which the child experienced the same ACE, rather than considering exposure to multiple types of ACEs.

### Co-occurring self-harm and depression

We differentiated between self-harm, depression and co-occurring self-harm and depression—a child was considered to have co-occurring self-harm and depression if they reported self-harm in the past 12 months (or between ages 12 and 18 years in the E-Risk study) and also scored above the established cut-offs for depression on the Short Mood and Feelings Questionnaire (or received a major depressive disorder diagnosis in the E-Risk study). To address the first aim, our categorical outcome was coded as follows—those reporting neither self-harm or depression were coded as 0, those reporting self-harm alone as 1, depression alone as 2 and both self-harm and depression as 3, the categories were mutually exclusive.

## Methods

### Pre-registration

The protocol for this study was pre-registered on the Open Science Framework (https://osf.io/uc5fk).

### Design and data sources

Data were from three UK cohorts.

#### The Avon Longitudinal Study of Parents and Children (ALSPAC)

ALSPAC is a birth cohort based in the South West of England. Pregnant women resident in Avon, United Kingdom, with expected dates of delivery from 1 April 1991 to 31 December 1992 were invited to take part (Boyd et al., 2013; Fraser et al., 2013). The initial number of pregnancies enrolled was 14,541. Of these initial pregnancies, there was a total of 14,676 foetuses, resulting in 14,062 live births and 13,988 children who were alive at 1 year of age (Boyd et al., 2013; Fraser et al., 2013). Data were collected via clinics and questionnaires completed by mothers, their partners and the children themselves (Boyd et al., 2013; Fraser et al., 2013). Study data were collected and managed using REDCap electronic data capture tools hosted at the University of Bristol. REDCap (Research Electronic Data Capture) is a secure, web-based software platform designed to support data capture for research studies (Harris et al., 2009). ALSPAC contains detailed data on family, socioeconomic and demographic factors before birth, as well as exposure to ACEs, and self-harm and depression measured over multiple time points in childhood, adolescence and adulthood. Further cohort details are in Appendix S1.

#### The Millennium Cohort Study (MCS)

MCS is a longitudinal study of children born between 2000 and 2002 in England, Scotland, Wales and Northern Ireland, recruited from a random sample of electoral wards in the UK (Connelly & Platt, 2014). This is a nationally representative sample of over 18,000 participants (18,552 families, 18,818 children at first wave of data collection); those from ethnic minority and socioeconomically disadvantaged areas were purposely over-sampled to ensure a representative sample (Connelly & Platt, 2014). Data on family demographic and socioeconomic characteristics, ACEs and mental health measures were collected at seven intervals from childhood to adolescence (9 months, and ages 3, 5, 7, 11, 14 and 17 years). Data were collected via surveys completed by the main parent, co-resident parent and children.

#### The Environmental Risk (E-Risk) Longitudinal Twin Study

E-Risk is a study of 2,232 same-sex twins born between 1994 and 1995 in England and Wales (Moffitt, 2002). This is a nationally representative sample—E-Risk families’ addresses are currently a near-perfect match to the deciles of the UK’s 2015 Lower-layer Super Output Area Index of Multiple Deprivation (IMD). Comprehensive data on a range of environmental risk factors and outcomes have been collected at multiple time-points from early childhood to adolescence (age 5, 7, 10, 12 and 18 years, with 93% retention), with data from birth records also accessible. Data were collected via home visits, with the primary caregiver and the twin participants during the childhood phases of the study and with twin participants at age 18.

### Measures

**Figure 1:**
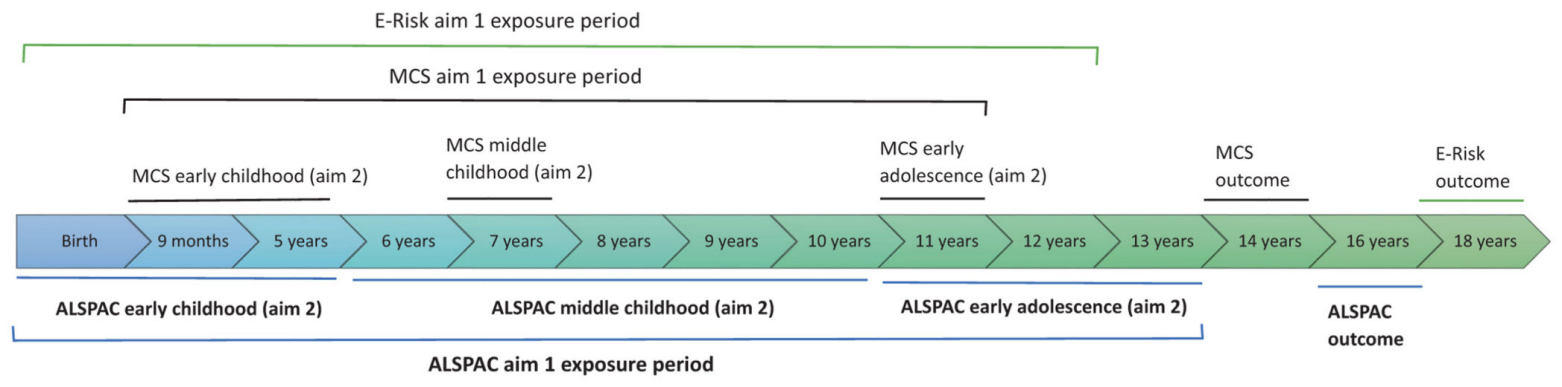
Timeline of exposure and outcome measures for each aim by cohort

Figure 1 shows the time-points for the study measures across the cohorts.

#### Adverse childhood experiences

ACEs data availability and measurement differed across cohorts; study-specific definitions and time-points for assessment of ACEs are presented in Table S1a,b.

#### ALSPAC cohort

Ten ACEs reported prospectively and retrospectively by parents and the children were examined: physical abuse, sexual abuse, emotional abuse, emotional neglect, bullying victimisation (henceforth referred to as ‘bullying’), parents’ violence towards each other (referred to as domestic violence), parental substance use, parental mental health problems, parental conviction and parental separation/divorce. We examined exposure to ACEs from birth to age 13 years, with each ACE dichotomised. The method of deriving the dichotomous variables from the ALSPAC data is described elsewhere (Houtepen, Heron, Suderman, Tilling, & Howe, 2018).

#### MCS cohort

Questions on exposure to six ACEs (physical abuse, bullying, domestic violence, parental mental health problems, parental substance use and parental separation/divorce) were completed by the main parent, the partner and child prospectively. Table S2 details the measures used to derive each dichotomous ACE, and Figure S1 the sample selection flow chart. The focus on exposure to ACEs was from 9 months to 11 years in this study.

#### E-Risk cohort

Face-to-face interviews with the primary caregiver (predominantly the mother) and the cohort members, plus researcher observations during home visits, involved collection of a range of prospective measures between ages 5 and 12 years on 11 ACEs: physical abuse, sexual abuse, emotional abuse and neglect, physical neglect, bullying victimisation, domestic violence, parental substance abuse, parental mental health problems, parental antisocial behaviour, parental separation/divorce (covering the period from birth to age 12 years). Five types of childhood maltreatment were reported retrospectively at age 18 years and included as separate binary variables in sensitivity analysis. A detailed description of the measures is provided in Appendix S2.

#### Developmental timing and duration of exposure

For aim 2, we derived measures of the occurrence of each type of ACE within early childhood (0–5 years), middle childhood (6–10 years) and early adolescence (11–13 years). Exposure measures were available in all potential critical periods (early childhood, mid childhood and adolescence) for six ACEs in ALSPAC and five ACEs in MCS. Exposure to parental mental health problems, domestic violence and parental separation were available in both cohorts. ALSPAC additionally had exposure measures for physical and emotional abuse and parental conviction, and MCS additionally had exposure measures for parental substance use and bullying. E-Risk did not have sufficient temporal resolution for exposure measures to be included in this analysis. See Table S3 for details.

#### Self-harm

Self-harm was defined as any act of intentional self-poisoning or self-injury regardless of suicidal intent (National Institute for Health and Care Excellence (NICE), 2013). We investigated past-year self-harm in ALSPAC and MCS (assessed at age 16 in ALSPAC, and age 14 in the MCS), and self-harm since age 12 in the E-Risk study assessed at age 18. These ages were chosen as both self-harm and depression measures were captured at these time-points. Self-harm was self-reported in all cohorts (see Appendix S3 for question wording).

#### Depression

The short mood and feelings questionnaire (SMFQ) measured the core symptoms of depression in ALSPAC and the MCS. The SMFQ comprises 13 questions measuring recent affective and cognitive symptoms in children and adolescents (Messer et al., 1995). We derived a dichotomous variable (yes/no) for depression to establish co-occurring self-harm and depression, using established cut-offs (scores ≥12) (Eyre et al., 2021). We examined depression at age 16 in ALSPAC and age 14 in MCS. This measure was not available in the E-Risk study. Instead, we included a measure of major depressive disorder within the previous 12 months which was assessed at age 18 by face-to-face interview using the DSM-IV criteria.

#### Covariates

We adjusted for the following covariates: household social class, parity, home ownership status, mother’s educational qualifications and mother’s age at delivery (Joinson, Kounali, & Lewis, 2017; Lodebo, Moller, Larsson, & Engstrom, 2017; Marryat & Frank, 2019; Page et al., 2014). Covariates were based on the best available and most comparable measures within each cohort. See Appendix S4 for further details.

### Statistical analyses

#### Missing data

To maximise sample size and minimise bias due to selective attrition of high-risk families, multiple imputation by chained equations was used to handle missing data in ALSPAC and the MCS (Azur, Stuart, Frangakis, & Leaf, 2011; White, Royston, & Wood, 2011). The detailed multiple imputation strategy for ALSPAC and MCS is presented in Appendix S5.

An available case analysis approach was adopted for the E-Risk study given minimal missing data on key variables, with 93% of the 2,232 twins retained at age 18 years.

##### Data analysis

Analyses were conducted in STATA/MP version 17.0 (StataCorp, USA) and R version 4.1.3.

##### Aim 1

To examine the relationships between each ACE with self-harm and depression, we used unadjusted and multivariable adjusted multinomial logistic regression models in STATA. We estimated the relationship between each ACE and the four-category outcome of neither self-harm or depression, self-harm alone, depression alone, both self-harm and depression. Where multiple imputation was used, results were combined using Rubin’s rules (Rubin, 1987). Sampling weights were applied to the MCS data to take the clustered sampling design into account. We used the cluster command to account for the non-independence of the twin observations in E-Risk analyses. As the effects of some ACEs differ by sex (Schilling, Aseltine Jr., & Gore, 2007), we also conducted exploratory analyses to examine the potential moderating effects of sex by including interaction terms between each ACE and sex, and also conducted subgroup analyses.

##### Aim 2

To examine the relationship between timing and duration of exposure to ACEs and risk of self-harm and depression in ALSPAC and MCS, a structured life-course modelling approach (SLCMA) was used (Mishra et al., 2009; Smith et al., 2015). This approach examines life-course hypotheses for how repeated measures of binary exposures over the life-course are associated with later outcomes (Mishra et al., 2009; Smith et al., 2015, 2016). It tests a set of hypotheses, identifying the best-fitting hypotheses supported by the data. We considered the accumulation and critical period hypotheses a priori as being most relevant for our outcome. The accumulation of risk hypothesis assumes that the log-odds of the outcome increase linearly with each unit change of the exposure, that is, each additional occasion on which the ACE is reported (Kuh et al., 2003). In this analysis, we focused on the recurrence of the same ACE. The critical period hypothesis assumes the outcome is only associated with exposure during specific developmental periods. We considered three potential critical periods: early childhood (0–5 years), middle childhood (6–10 years) and early adolescence (11–13 years).

We applied the SLCMA using least absolute shrinkage and selection operator (lasso) selection for binary logistic regression models in R, separately for each ACE and outcome, to identify the hypothesis or set of hypotheses that show the greatest relative improvement in model fit compared to the null model. Three binary outcome variables were examined separately: self-harm (vs. no self-harm), depression (vs. no depression) and co-occurring self-harm and depression (vs. neither self-harm or depression as well as those reporting self-harm alone or depression alone). This was necessary because SLCMA is not compatible with a categorical outcome of more than two levels. See Table S4 for further details, and Appendix S10 for the SLCMA code.

### Sensitivity analyses

Results were replicated among those who completed >50% of the ACE questions and had complete data on outcomes and confounders (*N* = 2,234). In the MCS, sensitivity analyses were conducted on households that completed all five sweeps of data collection for the exposure period and had complete data on the outcomes and confounders (*N* = 4,649). In the E-Risk study, we conducted a sensitivity analysis using retrospective child reports of exposure to maltreatment obtained via the Childhood Trauma Questionnaire (Bernstein & Fink, 1998) at age 18 (for the period 0–12 years) to examine if associations differed by prospectively or retrospectively reported ACEs (as it has previously been shown that these methods capture largely non-overlapping groups of victimised individuals in this cohort) (Newbury et al., 2018).

## Results

### Characteristics of the cohorts

Table 1 shows the key characteristics of the samples across the three cohorts. The most frequently reported ACE was parental mental health problems, where prevalence ranged from 45.9% by age 13 in ALSPAC, to 53.0% by age 11 in MCS, and 60.4% by age 12 years in E-Risk. The proportion of adolescents in ALSPAC with self-harm alone, depression alone and co-occurring self-harm and depression was 11.1%, 10.4% and 18.0%, respectively. In the MCS, the proportions were 6.5%, 7.3% and 8.1%, while in E-Risk, they were 6.4%, 12.3% and 7.8%. Tables S5 and S6 show the prevalence of ACEs, self-harm and depression and confounders in imputed and complete-case data in ALSPAC and MCS, respectively; Table S7 shows the prevalence in E-Risk. Tables S8–S10 show the prevalence of ACEs and confounders by outcome.

#### Aim 1: Associations between individual ACEs and depression and self-harm in adolescence

**Figure 2:**
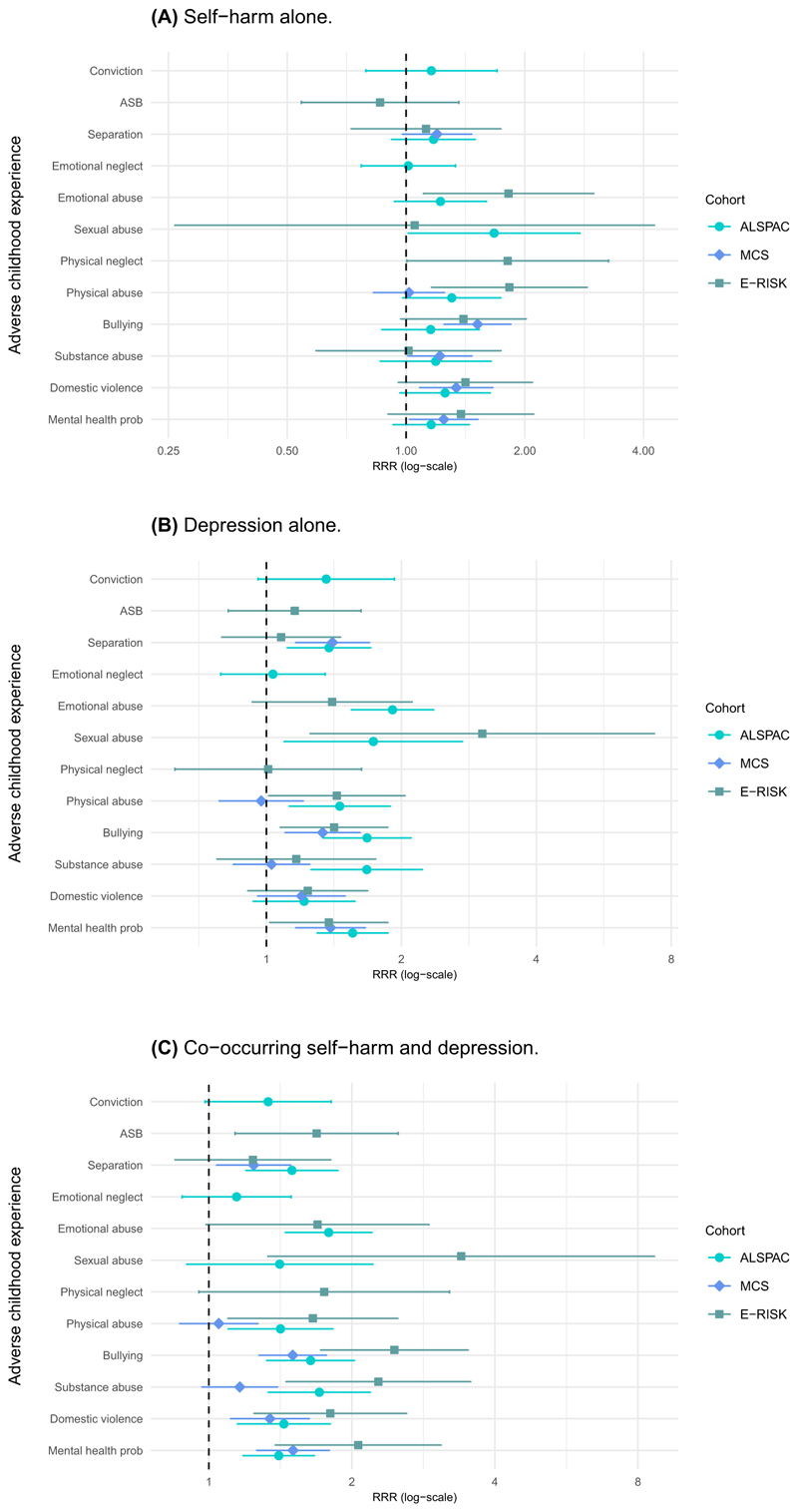
Forest plots showing the association between ACEs and outcomes by cohort. (A) Self-harm alone. (B) Depression alone. (C) Co-occurring self-harm and depression. *Note*: The dots on the forest plots represent the adjusted relative risk ratio (RRR) point estimates and the lines extending either side of them represent the 95% confidence intervals. The Avon Longitudinal Study of Parents and Children (ALSPAC); the Millennium Cohort Study (MCS); the Environmental Risk (E-Risk) Longitudinal Twin Study. ASB, Parental antisocial behaviour

**Figure 3:**
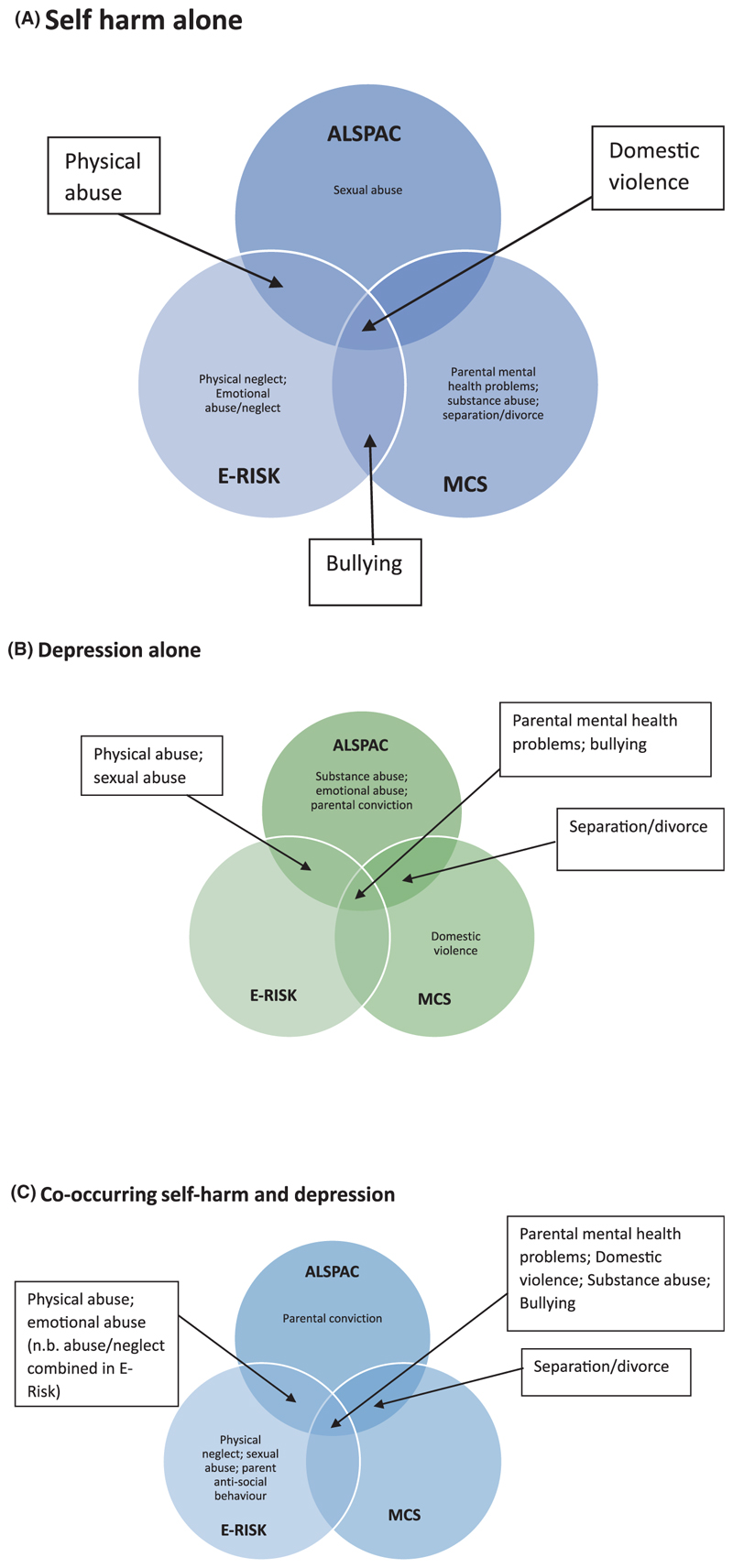
Comparison of the association between ACEs and self-harm alone, depression alone and co-occurring self-harm and depression across the three cohorts. The three Venn diagrams show comparison of the findings for the associations between ACEs and self-harm alone, depression alone and co-occurring self-harm and depression by cohort (The Avon Longitudinal Study of Parents and Children (ALSPAC); The Millennium Cohort Study (MCS); The Environmental Risk (E-Risk) Longitudinal Twin Study). Results are shown where there is evidence of some association according to the 95% confidence intervals for adjusted relative risk ratios

The associations between individual ACEs and co-occurring self-harm and depression, self-harm alone and depression alone are shown in Table 2 (presented as adjusted relative risk ratios [aRRR], with crude RRRs presented in Table S11). Most adversities were associated with an increased risk of co-occurring self-harm and depression across all three cohorts (7/10 ACEs in ALSPAC; 5/6 in MCS; and 9/10 in E-Risk; Figure 2). Associations between most ACEs and self-harm alone or depression alone were also found in at least one of the three cohorts. Figure 3 summarises findings across the three cohorts.

#### Self-harm alone and depression alone

There was less consistency across the cohorts for the association between ACEs and self-harm alone than for the other outcomes (Figure 2). For depression alone, associations were consistent across all three cohorts for parental mental health problems and bullying, and for physical and sexual abuse in ALSPAC and E-Risk, and separation/divorce in ALSPAC and MCS.

#### Co-occurring self-harm and depression

Parental mental health problems, domestic violence, bullying and substance abuse were associated with increased risk of co-occurring self-harm and depression among all three cohorts (Figure 2). In addition, associations with parental separation/divorce, physical abuse and emotional abuse/neglect were found in two cohorts.

#### Interaction by sex

There were few sex differences, and these were not consistent by cohort; see Appendix S6 and Table S12a,b for details.

#### Aim 2: Developmental timing and duration of exposure to ACEs

**Figure 4:**
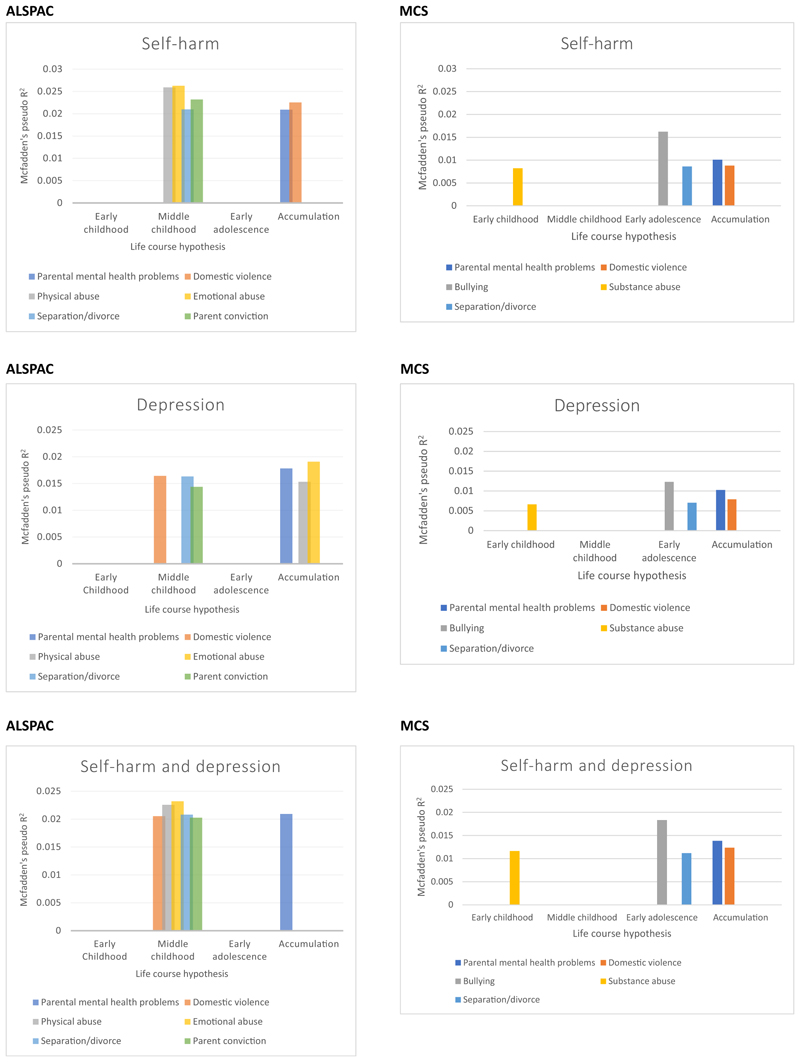
The best-fitting life-course hypothesis selected by the least absolute shrinkage and selection operator (lasso) for the association between each adverse childhood experience and self-harm, depression and co-occurring self-harm and depression (at age 16 years) in ALSPAC and (at age 14 years) MCS, multiply imputed data. Early childhood refers to exposure between birth and 5 years of age, middle childhood refers to exposure between 6 and 10 years of age, early adolescence refers to exposure between 11 and 13 years of age and accumulation refers to cumulative exposure to the same ACE across multiple time-points in early childhood, middle childhood and adolescence

Figures S2a,b shows the elbow plots of the proportion of outcome variation explained by the selected hypotheses for ACE and outcome measure, after adjusting for confounders in ALSPAC and MCS. Figure S2c shows tetrachoric correlations for each ACE over time. As the lasso algorithm first selects the hypothesis with the strongest association with the outcome—the simplest hypothesis that is most supported by the observed data, we restricted our reporting to this due to the large number of models and exposures being examined (Smith et al., 2015). Figure 4 shows the first hypothesis that was best supported by the ALSPAC data, separately for each outcome measure, and Table 3 shows the effect estimates for the selected hypothesis in ALSPAC and MCS. A summary of the results for all outcomes and both cohorts can be found in Table 4. Here, we focus on the outcome of co-occurring self-harm and depression, the primary focus of this paper.

For parental mental health, accumulation of exposure was the best-fitting model in both ALSPAC and MCS, with a strong association between cumulative exposure to parental mental health problems and increased odds of co-occurring self-harm and depression (Table 3). However, for domestic violence, parental separation, physical abuse, emotional abuse and parental conviction, the middle childhood period was selected for each of these ACEs in ALSPAC, with exposure during this period associated with increased odds of co-occurring self-harm and depression. In the MCS, accumulation of exposure was the best model for domestic violence, with cumulative exposure associated with increased odds of co-occurring self-harm and depression. However, for bullying and parental separation, early adolescence as a sensitive period was best supported by the MCS data, with effects estimates showing increased odds of co-occurring self-harm and depression. Early childhood was selected for substance abuse, with exposure during this period associated with increased odds of co-occurring self-harm and depression. These results suggest little consistency in the best-fitting life-course hypothesis across different ACEs and across different cohorts.

The elbow plots (Figures S2a,b) show McFadden’s pseudo-*R*^2^ explained by each model after adding each additional life-course hypothesis. There was a consistent pattern in the ALSPAC data where middle childhood and accumulation of exposure were the most frequently selected hypotheses supported by the data. For physical and emotional abuse, separation/divorce and parental conviction, there is an elbow at two variables on the plots for the outcome co-occurring self-harm and depression, which are middle childhood plus accumulation of exposure. This means being exposed increases the odds at all time-points, but there is a stronger increase if exposed in middle childhood. However, the p-values for adding a second hypothesis showed there was no strong statistical evidence for improvement to the model fit associated with adding another hypothesis (all *p* > .05). Therefore, the first hypothesis selected is most strongly associated with co-occurring self-harm and depression for these ACEs, adding additional hypotheses beyond this point did not considerably improve the pseudo-*R*^2^ values.

In the MCS, for separation/divorce, there was also an elbow at two hypotheses, adolescence plus an early childhood sensitive period; however, there was no strong statistical evidence for improvement to the model fit associated with adding another hypothesis (*p* = .130).

### Sensitivity analyses

ALSPAC complete-case results, E-Risk results using retrospective reports of ACEs, and MCS complete-case results are reported in Appendices - S7-S9 and Tables S13a,b, S14a,b, respectively, and produced broadly similar findings to the main analyses.

## Discussion

This prospective study used data from over 22,000 children and adolescents from three UK cohorts to examine the associations between individual ACEs with self-harm and depression. Most adversities were associated with co-occurring self-harm and depression, with consistent findings across cohorts. The accumulation of parental mental health problems was more strongly associated with increased likelihood of co-occurring self-harm and depression in adolescence relative to exposure in specific developmental periods, in both ALSPAC and MCS. For other ACEs, middle childhood emerged as an important period in ALSPAC, whereas in the MCS, early childhood and adolescence were more important.

ACEs have previously been linked to self-harm and depression in a dose–response manner, with an increasing risk as the number of ACEs increases (Elmore & Crouch, 2020; Russell et al., 2021). Studies have commonly examined this association using ACE scores as predictors, or by dichotomising number of ACEs (e.g. <4 vs. 4+) (Bomysoad & Francis, 2020; Elmore & Crouch, 2020; Russell et al., 2019). However, ACE scores could obscure variations in how different ACEs are associated with outcomes; we found associations for ACEs varied by outcome and also across cohorts. For example, parental mental health problems were associated with self-harm alone in only one cohort whereas associations were seen for depression alone and co-occurring self-harm and depression across all three cohorts. Examining ACEs separately can help identify ACEs that are consistently associated with specific outcomes across different cohorts, as well as those that exhibit differential effects. For instance in the MCS, physical abuse was not associated with any outcome, in contrast to the ALSPAC and E-Risk study findings. Similarly, findings for separation/divorce were not consistent in E-Risk compared to ALSPAC and MCS. Our findings provide evidence to support the concern that summing ACE’s together into a total score can disguise important differences in the effects of different adversities (Lacey & Minnis, 2020).

We found that the accumulation of certain ACEs (i.e. cumulative exposure to the same ACE at multiple time-points in childhood and adolescence) such as parental mental health problems was the best model for predicting co-occurring self-harm and depression in adolescence across ALSPAC and MCS. These findings are consistent with another study in ALSPAC, linking cumulative maternal depression exposure and elevated risk of depression among offspring in early adulthood (Lacey et al., 2023). Although the variance explained by the life-course models in this study appear low, it is in line with previous findings from population-based cohorts that examine temporal patterns of ACEs (Dunn, Soare, et al., 2018; Lacey et al., 2023). The absolute contribution of our explanatory models to the variance in the outcomes of self-harm and depression in adolescent populations is small, this highlights the scale and complexity of untangling the epidemiology of mental health problems.

Middle childhood emerged as an important period for the association between several ACEs and co-occurring self-harm and depression in the ALSPAC cohort. The significance of this period in our study may be explained by the rapid development of emotion regulation skills, and the role of attachment, which shifts from proximity to the availability of the attachment figure during this period (Bosmans & Kerns, 2015; Fields & Prinz, 1997). Disruptions to the development of appropriate emotion regulation strategies due to insecure attachment with caregivers in middle childhood may contribute to the development of maladaptive behaviours such as self-harm to regulate negative emotions, and insecure attachment and emotion dysregulation have been shown to mediate the relationship between ACEs and self-harm and depression (Christoforou & Ferreira, 2022; Cloitre et al., 2019; Ye, Wei, Zhang, Li, & Cao, 2023). Parental mental illness and family disruption in middle childhood have been linked to greater emotion dysregulation in adulthood compared to exposure in other periods (Dunn, Nishimi, Gomez, Powers, & Bradley, 2018). Other studies also provide support for the importance of the middle childhood period; physical abuse in middle childhood compared to early childhood and adolescence has been shown to be associated with depression (Adams, Mrug, & Knight, 2018); there is also some evidence of recency and exposure to physical or sexual abuse during middle childhood being associated with emotional and behavioural problems among females at age 8 years (Dunn, Soare, et al., 2018). Maternal depression has been linked to increasing depressive symptoms among female offspring in early adulthood, although the best-fitting hypothesis for the whole sample was accumulation of exposure (consistent with our study), among females the largest effects were seen in middle childhood, relative to other periods (Lacey et al., 2023). Middle childhood as a sensitive period has also emerged in other SLCMA studies; exposure to ACEs such as sexual or physical abuse or socioeconomic instability during this period has been linked to DNA methylation (Dunn et al., 2019; Liu et al., 2022), and maternal psychopathology has been linked to accelerated epigenetic ageing among females. Although, in this study, accumulation of exposure to parental mental health problems best supported our data, we did not examine sex differences or disaggregate maternal and paternal mental health problems (Marini et al., 2020).

Our findings diverge from earlier literature emphasising early childhood as the most sensitive period, whereby exposure to adversity can disrupt brain development, heightening vulnerability to mental health problems (Fox, Levitt, & Nelson, 2010; McLaughlin et al., 2011). One study using retrospective self-reports of maltreatment found first exposure to maltreatment during early childhood was most strongly associated with depression and suicidal ideation compared to other time points in childhood and adolescence (Dunn et al., 2013). This study examined age of onset of maltreatment, whereas our study did not explore first age of exposure. Differences could also be due to measurement and reporting of ACEs. The earlier childhood measures in ALSPAC were collected through parents’ reports, children reported prospectively from age 8 in middle childhood and retrospectively at age 21–23 about the whole of childhood (split as <11 and ≥11 years). One study comparing prospective informant reports and retrospective self-reports of childhood maltreatment found the strongest associations between these reports and psychiatric problems at age 18 years were found when maltreatment was retrospectively self-reported (Newbury et al., 2018). Adults who recall being maltreated may have a particularly elevated risk for mental health problems. In the MCS, all of the ACEs, except bullying, were reported by the parents, which could explain differences in the results across cohorts.

In MCS, early childhood and adolescence were more important than middle childhood. Substance abuse and bullying were not examined across developmental periods in ALSPAC, and thus, the findings could not be compared. Cohort effects may partly explain variations across cohorts in the effects of some ACEs such as parental separation/divorce. For example, the MCS population experienced the 2008 financial recession and subsequent austerity measures during their childhood and early adolescence, whereas the ALSPAC participants experienced this in later adolescence/early adulthood. Financial difficulties as a result of cuts to family benefits, increases in unemployment and an increase in child poverty between 2010 and 2011 may contribute to the potentially harmful effects of parental separation during adolescence, particularly for single parent households in MCS (Bradshaw & Main, 2017; Cantillon, Chzhen, Handa, & Nolan, 2017). The heightened sensitivity during adolescence may make them more vulnerable to these factors.

The greater inconsistency in findings across cohorts for the outcome self-harm alone may reflect the different functions of self-harm that are not related to depression. Self-harm without depression may include depression symptoms that do not meet the threshold for a clinical diagnosis, and it could also reflect other functions of self-harm that are not associated with psychiatric comorbidity. While psychiatric disorders, particularly depression and anxiety, are more prevalent in people who self-harm, self-harm can occur in the absence of psychiatric comorbidity (Hawton, Saunders, et al., 2013; Nitkowski & Petermann, 2011; Wilkinson, 2013). Perhaps in our cohorts, self-harm without the presence of depressive symptoms may reflect these other functions. In studies of young people that self-harm, attention deficit hyperactivity disorder (ADHD), anxiety disorders, conduct disorder, adjustment disorder and eating disorders are also common (Hawton, Saunders, et al., 2013). This suggests self-harm without depression may co-occur with other mental disorders that were not examined in this study.

### Strengths and limitations

This study has several strengths. We used three large cohorts, including two nationally representative samples, to ensure findings were generalisable to the UK population. In MCS, those from ethnic minority and socioeconomically disadvantaged areas were oversampled to ensure a representative sample of the UK population. In E-Risk, younger mothers were oversampled and older mothers were undersampled, resulting in a sample which almost perfectly represents the socio-economic distribution of the UK population. This addresses the limitations of ALSPAC which, while providing repeated measures on a range of ACEs, is an ethnically homogenous population from an affluent area. Second, all cohorts have rich prospective and/or retrospective measures on a range of ACEs measured repeatedly from birth to adolescence, enabling us to ensure temporality between the exposures and outcomes.

There are some limitations that must be acknowledged. First, ACEs rarely occur in isolation, and experiencing one adversity increases the risk of experiencing more (Hughes et al., 2017; Lacey, Howe, Kelly-Irving, Bartley, & Kelly, 2022). As such, it is unclear whether results for individual ACEs reflect qualitative differences or the effects of other co-occurring ACEs. This study did not address this co-occurrence using common methods such as the score-based approach, whereby the total number of ACEs each individual is exposed to is summed to derive a total score. This approach does not offer insights into which specific ACEs co-occur or whether certain combinations of ACEs have distinct impacts on self-harm and depression, and assumes each ACE is equally important for health outcomes. ACE co-occurrence can also be examined by employing person-centred approaches such as latent class analysis (LCA) to examine how adversities cluster (Lacey et al., 2022); however, model fit in these approaches is often poor and the identified latent classes do not often replicate across studies. These typical approaches to examining co-occurring ACEs, therefore, have severe limitations. While our chosen approach of examining individual ACEs allows us to examine specificity of effects, it must be acknowledged that the limitation of this approach is that we do not identify whether those who have exposure to multiple ACEs are at higher risk of co-occurring self-harm and depression. While cumulative exposure to the same ACE type over time was examined, the effects of multiple ACE types were not addressed. Furthermore, power prohibited interaction analysis in the SLCMA as previous research has found differences by sex in the best-fitting life-course models (Lacey et al., 2023). Second, these results do not reflect causal relationships between ACEs with self-harm and depression as we did not account for all confounding factors, such as unmeasured genetic influences and the effect of other co-occurring ACEs (Baldwin et al., 2023).

Third, there were differences in our findings that could be due to features of ACEs measurement and data availability, or the cohort characteristics. The considerably higher prevalence of separation/divorce in the E-Risk study (48%) compared to ALSPAC (30%) and MCS (28%) could be a feature of the twin cohort. Previous work examining the association between twin births and parental divorce has also found that twins at first birth were associated with higher likelihood of divorce compared to singletons (Jena, Goldman, & Joyce, 2011). Parents of twins experience higher levels of parenting stress, depression and anxiety than singletons, which could place greater strain on the relationship—also explaining the higher prevalence of parental mental health problems and domestic violence in the E-Risk cohort (Olivennes, Golombok, Ramogida, & Rust, 2005; Wenze, Battle, & Tezanos, 2015). The high prevalence of substance misuse in the MCS could be due to differences in measurement, as both ALSPAC and E-Risk had more comprehensive measures that captured harmful substance use; however, the measures in the MCS, particularly for alcohol use, captured high frequency of consumption and not necessarily alcohol use at harmful levels.

Physical abuse was also captured differently in MCS (defined as exposure to smacking) compared to ALSPAC and E-Risk, potentially explaining the lack of associations between physical abuse and outcomes in MCS. Frequency and timing of ACE measurement also varied, with fewer data collection points in MCS compared to ALSPAC, potentially impacting prevalence estimates. Additionally, due to data limitations, not all ACEs could be examined across all cohorts, preventing replication. E-Risk’s twin cohort may differ due to genetic and environmental factors. Findings for parental separation/divorce differed among E-Risk participants compared to the other cohorts, with no evidence for an association found. Twin siblings’ emotional support and unique bond may mitigate the effects of family disruption compared to singletons (Fortuna, Goldner, & Knafo, 2010).

We did not account for genetic confounding, which may contribute to the associations between ACEs with self-harm and depression (Baldwin et al., 2019). One recent study found that elevated risk of mental health problems in children exposed to ACEs was partly due to pre-existing genetic risk (Baldwin et al., 2023). However, for some ACEs—such as childhood maltreatment and parental mental illness —associations with mental health problems persisted independent of genetic confounding. Future research should use genetically informative methods to understand the extent to which genetic confounding contributes to the associations between ACEs with self-harm across development.

## Conclusions

If found to be causal, our findings support the importance of primary interventions to prevent the accumulation of some ACEs, and secondary interventions to mitigate their impact on self-harm and depression in adolescence. Prevention for ACEs has largely focused on the perinatal and early-life period; whereas our findings showing a sensitive period in middle childhood (and elevated risks linked to cumulative exposure) highlight the importance of continuing to address ACEs throughout childhood, particularly during middle childhood (Asmussen, Fischer, Drayton, & McBride, 2020). Given parental mental health problems was the ACE most frequently reported in this study, and its cumulative effect was particularly apparent (consistent across outcomes and across cohorts), preventing parental mental illness and mitigating the impact on children is crucial. It is particularly important since parental mental illness has an effect on child mental health, independent of genetic transmission (Baldwin et al., 2023) and evidence suggesting that vigorous treatment of maternal depression to achieve remission is also associated with a reduction in diagnoses and internalising and externalising symptoms in their children (Weissman et al., 2006). Preventing mental health problems in adolescents is crucial, as many mental health problems emerge during this period (Kim-Cohen et al., 2003). Implementing preventative approaches can reduce ACE prevalence in future generations as parental ACEs are risk factors for ACEs in their children (Narayan, Lieberman, & Masten, 2021).

## Supplementary Material

Supporting Information

## Figures and Tables

**Table 1:** The prevalence of adverse childhood experiences, outcomes and demographic characteristics for each cohort

	ALSPAC^a^ (*n* = 9,511) %	MCS^a^ (*n* = 11,008) %	E-Risk study^b^ (*n* = 2,232) %
Adverse childhood experience^c^			
Parental mental health problems	45.9	53.0	60.4
Domestic violence	24.2	27.3	45.8
Substance abuse	14.5	30.6	16.2
Bullying	15.9	40.4	44.8
Physical abuse	15.1	25.2	20.1
Physical neglect	–	–	9.0
Sexual abuse	3.5	–	1.6
Emotional abuse/neglect	–	–	11.6
Emotional abuse	24.4	–	–
Emotional neglect	18.3	–	–
Separation/divorce	29.9	27.9	48.4
Parental antisocial behaviour	–	–	25.4
Parental conviction	8.8	–	–
Outcome^d^			
Neither self-harm or depression	60.5	78.1	73.5
Self-harm alone	11.1	6.5	6.4
Depression alone	10.4	7.3	12.3
Co-occurring self-harm and depression	18.0	8.1	7.8
Sex			
Male	49.2	49.7	48.9
Female	50.8	50.3	51.1
Ethnicity			
White	93.9	82.0	90.4
Non-White	6.1	18.0	9.6

a Pooled proportions from multiply imputed data.

b Denominator varies according to missing data for each variable.

c Between birth and 13 years in The Avon Longitudinal Study of Parents and Children (ALSPAC); between 9 months and 11 years in the Millennium Cohort Study (MCS); between birth and 12 years in The Environmental Risk (E-Risk) Longitudinal Twin Study.

d By age 16 in ALSPAC; by age 14 in the MCS; self-harm between ages 12 and 18, and depression within the past 12 months at age 18 in E-Risk.

**Table 2:** Adjusted relative risk ratios for the association between adverse childhood experiences and self-harm and depression among each cohort

Adverse childhood experience	ALSPAC				MCS				E-Risk		
	Self-harm alone^a^ (*n* = 993) RRR (95% CI)	Depression alone^a^ (*n* = 973) RRR (95% CI)	Co-occurring self-harm and depression^a^ (*n* = 1,735) RRR (95% CI)		Self-harm alone (*n =* 714) RRR (95% CI)	Depression alone (*n* = 801) RRR (95% CI)	Co-occurring self-harm and depression (*n* = 893) RRR (95% CI)		Self-harm alone (*n* = 132) RRR (95% CI)	Depression alone (*n* = 253) RRR (95% CI)	Co-occurring self-harm and depression (*n* = 161) RRR (95% CI)
Parental mental health problems	1.16 (0.92 –1.45)	1.56 (1.30 –1.87)	1.40 (1.18–1.67)		1.25 (1.02 –1.52)	1.39 (1.16 –1.66)	1.50 (1.26–1.79)		1.38 (0.90 –2.11)	1.38 (1.02 –1.87)	2.06 (1.38–3.08)
Domestic violence	1.26 (0.96 –1.64)	1.21 (0.93 –1.58)	1.44 (1.15–1.81)		1.34 (1.08 –1.66)	1.20 (0.96 –1.50)	1.34 (1.11–1.63)		1.41 (0.96 –2.09)	1.24 (0.91 –1.68)	1.80 (1.24–2.61)
Substance abuse	1.19 (0.86 –1.65)	1.67 (1.25 –2.24)	1.71 (1.33–2.19)		1.22 (1.01 –1.47)	1.03 (0.84 –1.25)	1.16 (0.97–1.40)		1.01 (0.59 –1.74)	1.17 (0.78 –1.75)	2.27 (1.45–3.56)
Bullying	1.15 (0.87 –1.54)	1.68 (1.34 –2.11)	1.64 (1.32–2.03)		1.52 (1.25 –1.85)	1.33 (1.10 –1.62)	1.50 (1.27–1.77)		1.40 (0.97 –2.02)	1.42 (1.07 –1.87)	2.46 (1.72–3.51)
Physical abuse	1.31 (0.98 –1.75)	1.46 (1.12 –1.89)	1.42 (1.09–1.83)		1.02 (0.83 –1.25)	0.97 (0.78 –1.21)	1.05 (0.87–1.27)		1.83 (1.16 –2.88)	1.44 (1.01 –2.04)	1.66 (1.10–2.50)
Physical neglect	–	–	–		–	–	–		1.81 (1.00 –3.26)	1.01 (0.63 –1.63)	1.75 (0.95–3.21)
Sexual abuse	1.67 (1.01 –2.77)	1.73 (1.09 –2.74)	1.41 (0.89–2.22)		–	–	–		1.05 (0.26 –f.26)	3.03 (1.25 –7.34)	3.40 (1.33–8.67)
Emotional abuse^b^	1.22 (0.93 –1.60)	1.91 (1.55 –2.36)	1.79 (1.45–2.21)		–	–	–		1.82 (1.11 –2.99)	1.40 (0.93 –2.11)	1.69 (0.99–2.91)
Emotional neglect	1.01 (0.77 –1.34)	1.03 (0.79 –1.35)	1.14 (0.88–1.50)		–	–	–		–	–	–
Separation/divorce	1.17 (0.92 –1.50)	1.38 (1.11 –1.71)	1.50 (1.19–1.88)		1.20 (0.98 –1.47)	1.40 (1.16 –1.70)	1.24 (1.04–1.49)		1.12 (0.73 –1.74)	1.08 (0.79 –1.46)	1.24 (0.85–1.81)
Parental conviction	1.16 (0.79 –1.71)	1.36 (0.95 –1.94)	1.33 (0.98–1.82)		–	–	–		–	–	–
Parental antisocial behaviour									0.86 (0.54 –1.36)	1.16 (0.82 –1.63)	1.69 (1.14–2.50)

The Avon Longitudinal Study of Parents and Children (ALSPAC): Adjusted for household social class, parity, home ownership status, mother’s educational qualifications, mother’s age at delivery. The Millennium Cohort Study (MCS): Adjusted for child and mother’s ethnicity, mother’s age at delivery, mother’s education, household income, family housing tenure. The Environmental Risk (E-Risk) Longitudinal Twin Study: Adjusted for child’s sex, household social class, mother’s age at delivery, mother’s qualifications and the non-independence of twin observations. RRR, relative risk ratio; 95% CI, 95% Confidence Interval.

a Reference group: neither self-harm or depression (ALSPAC *n* = 5,810 (from *m* = 1); MCS *n* = 8,600; E-Risk *n* = 1,515).

b And/or emotional neglect in the E-Risk study.

**Table 3:** Adjusted odds ratios and 95% confidence intervals for the association between the first life-course hypothesis selected by the least absolute shrinkage and selection operator (lasso) and each outcome measure among the ALSPAC and MCS cohorts, multiply imputed data

Adverse childhood experience	Self-harm				Depression				Co-occurring self-harm and depression		
	Hypothesis	OR (95% CI)	*p*-Value		Hypothesis	OR (95% CI)	*p*-Value		Hypothesis	OR (95% CI)	*p*-Value
ALSPAC											
Parental mental health problems	Accumulation	1.14 (1.07–1.20)	<.001		Accumulation	1.23 (1.17–1.30)	<.01		Accumulation	1.18 (1.10–1.25)	<.001
Domestic violence	Accumulation	1.28 (1.18–1.39)	<.001		Middle childhood	1.37 (1.22–1.54)	<.001		Middle childhood	1.41 (1.23–1.61)	<.001
Physical abuse	Middle childhood	2.45 (1.97–3.04)	<.001		Accumulation	1.54 (1.35–1.75)	<.001		Middle childhood	2.24 (1.78–2.82)	<.001
Emotional abuse	Middle childhood	1.97 (1.69–2.29)	<.001		Accumulation	1.55 (1.42–1.68)	<.001		Middle childhood	2.05 (1.73–2.42)	<.001
Separation/divorce	Middle childhood	1.43 (1.26–1.62)	<.001		Middle childhood	1.59 (1.40–1.80)	<.001		Middle childhood	1.54 (1.33–1.78)	<.001
Parental conviction	Middle childhood	2.26 (1.77–2.88)	<.001		Middle childhood	1.98 (1.55–2.52)	<.001		Middle childhood	2.18 (1.68–2.83)	<.001
MCS											
Parental mental health problems	Accumulation	1.13 (1.07–1.19)	<.001		Accumulation	1.19 (1.13–1.25)	<.001		Accumulation	1.18 (1.11–1.26)	<.001
Domestic violence	Accumulation	1.15 (1.08–1.23)	<.001		Accumulation	1.14 (1.07–1.21)	<.001		Accumulation	1.16 (1.07–1.25)	.001
Bullying	Early adolescence	1.81 (1.60–2.05)	<.001		Early adolescence	1.77 (1.57–2.00)	<.001		Early adolescence	1.83 (1.57–2.15)	<.001
Substance abuse	Early childhood	1.21 (1.07–1.37)	.008		Early childhood	1.09 (0.96–1.23)	.759		Early childhood	1.25 (1.07–1.46)	.020
Separation/divorce	Early adolescence	1.28 (1.12–1.47)	.001		Early adolescence	1.28 (1.16–1.46)	.002		Early adolescence	1.29 (1.08–1.53)	.018

Bonferroni correction applied to *p*-values. 95% CI, 95% Confidence interval; ALSPAC, The Avon Longitudinal Study of Parents and Children; MCS, The Millennium Cohort Study; OR, Odds ratio.

**Table 4:** Summary of ALSPAC and MCS findings on the best-fitting hypothesis selected by the least absolute shrinkage and selection operator (lasso) on the timing and duration of exposure to ACEs

	Self-harm			Depression			Co-occurring self-harm and depression			Direction of effect
	ALSPAC	MCS		ALSPAC	MCS		ALSPAC	MCS		
Parental mental health problems	Accumulation of exposure	Accumulation of exposure		Accumulation of exposure	Accumulation of exposure		Accumulation of exposure	Accumulation of exposure		Positive
Domestic violence	Accumulation of exposure	Accumulation of exposure		Middle childhood	Accumulation of exposure		Middle childhood	Accumulation of exposure		Positive
Separation/divorce	Middle childhood	Early adolescence		Middle childhood	Early adolescence		Middle childhood	Early adolescence		Positive
Physical abuse	Middle childhood	–		Accumulation of exposure	–		Middle childhood	–		Positive
Emotional abuse	Middle childhood	–		Accumulation of exposure	–		Middle childhood	–		Positive
Parental conviction	Middle childhood	–		Middle childhood	–		Middle childhood	–		Positive
Substance abuse	–	Early childhood		—	Early childhood		—	Early childhood		Positive
Bullying	–	Early adolescence		—	Early adolescence		—	Early adolescence		Positive

Early childhood refers to exposure between birth and 5 years of age, middle childhood refers to exposure between 6 and 10 years of age, early adolescence refers to exposure between 11 and 13 years of age and accumulation of exposure refers to cumulative exposure to the same ACE across multiple time-points in early childhood, middle childhood and early adolescence. Direction of effect: A positive direction of effect refers to increased odds of each outcome in relation to the best-fitting hypothesis selected by the lasso. For example, accumulation of exposure for the ACE parental mental health problems means that accumulation of parental mental health problems is associated with increased odds of self-harm, depression and co-occurring self-harm and depression in both ALSPAC and MCS, or exposure to domestic violence in middle childhood in ALSPAC is associated with increased odds of depression, and co-occurring self-harm and depression.

## Data Availability

Access to ALSPAC and E-Risk is not publicly available, access to ALSPAC can be requested via the study website (http://www.bristol.ac.uk/alspac/researchers/access/) and free managed access to E-Risk via a study sponsor (https://eriskstudy.com/data-access/). Access to the MCS data set is available via the UK Data Service (https://beta.ukdataservice.ac.uk/datacatalogue/series/series?id=2000031).

## Key points

- Childhood adversities are linked to increased risk of self-harm and depression in a dose–response manner. However, less is known about the impact of individual ACEs on co-occurring self-harm and depression.
- We examined associations between individual ACEs and co-occurring self-harm and depression, and tested whether these associations varied according to the developmental timing and duration of ACE occurrence.
- Across three prospective longitudinal UK cohorts, we found most ACEs were associated with increased risk of co-occurring self-harm and depression.
- There was considerable variation in the importance of timing and duration of ACEs for co-occurring self-harm and depression across ACEs and across cohorts. We found a detrimental impact of the accumulation of parental mental health problems over time in two cohorts. Additionally, middle childhood emerged as an important period for the association between most ACEs and co-occurring self-harm and depression in ALSPAC. Whereas in the MCS early childhood and adolescence appeared to be more important.
- Prevention efforts should focus on early intervention, with continued support throughout childhood, particularly middle childhood, to prevent persistent exposure to ACEs.
