# Supplementary material for: The relationship between type, timing and duration of exposure to adverse childhood experiences and adolescent self-harm and depression: findings from three UK prospective population-based cohorts
Source: J Child Psychol Psychiatry. Author manuscript; Available in PMC 2024 Nov 25. (PMC7616850; doi:10.1111/jcpp.13986)
Supplement: Supporting Information [file EMS199949-supplement-Supporting_Information.docx]

**Supporting Information**

The relationship between type, timing and duration of exposure to adverse childhood experiences and adolescent self-harm and depression: Findings from three UK prospective population-based cohorts

**Contents**

| Appendix S1 | The Avon Longitudinal Study of Parents and Children (ALSPAC) study population |
| --- | --- |
| Table S1a | Study definitions of adverse childhood experiences (ACEs) |
| Table S1b | Time point for the assessment of ACEs |
| Table S2 | Derivation of ACEs in the Millennium Cohort Study (MCS) |
| Figure S1 | MCS sample selection flowchart |
| Appendix S2 | Measures of ACEs in the Environmental Risk (E-Risk) Longitudinal Twin Study |
| Table S3 | Data availability by developmental period, for ACEs in ALSPAC and MCS |
| Appendix S3 | Measures of self-harm in each cohort |
| Appendix S4 | Covariates |
| Appendix S5 | Multiple imputation strategy for ALSPAC and MCS |
| Table S4 | Description of the life course models tested using the Structured Life Course Modelling Approach (SLCMA) |
| Table S5 | Prevalence of ACEs, outcomes, and confounders in multiply imputed and complete-case data in ALSPAC |
| Table S6 | Prevalence of ACEs, outcomes and confounders in multiply imputed and complete-case data in the MCS |
| Table S7 | Prevalence of ACEs, outcomes, and confounders in the E-Risk Study |
| Table S8 | Prevalence of ACEs and confounders by outcome – pooled proportions from multiply imputed data, ALSPAC |
| Table S9 | Prevalence of ACEs and confounders by outcome – pooled proportions from multiply imputed data, MCS |
| Table S10 | Prevalence of ACEs and confounders by outcome in the E-Risk study |
| Table S11 | Crude relative risk ratios for the association between ACEs and outcomes among each cohort |
| Appendix S6 | Interactions by sex |
| Table S12a | Stratified by sex, adjusted relative risk ratios for the association between ACEs and outcomes among males |
| Table S12b | Stratified by sex, adjusted relative risk ratios for the association between ACEs and outcomes among females |
| Figure S2a | Elbow plots: ALSPAC multiply imputed data |
| Figure S2b | Elbow plots: MCS multiply imputed data |
| Figure S2c | Tetrachoric correlations for each ACE over time in ALSPAC and MCS |
| Appendix S7 | ALSPAC complete-case sensitivity analyses |
| Table S13a | Crude and adjusted relative risk ratios for the association between ACEs and outcomes in ALSPAC complete-cases |
| Table S13b | Effect estimates for the association between the first life course hypothesis selected by the Least Absolute Shrinkage and Selection Operator (lasso) and each outcome measure among the ALSPAC complete-cases |
| Appendix S8 | The association between retrospectively reported ACEs and outcomes in the E-Risk study |
| Appendix S9 | MCS complete-case sensitivity analyses |
| Table S14a | Crude and adjusted relative risk ratios for the association between ACEs and outcomes in MCS complete-cases |
| Table S14b | Effect estimates for the association between the first life course hypothesis selected by the Least Absolute Shrinkage and Selection Operator (lasso) and each outcome measure among the MCS complete-cases |
| Appendix S10 | SLCMA study code |

Appendix S1: ALSPAC study population

Pregnant women resident in Avon, UK with expected dates of delivery between 1st April 1991 and 31st December 1992 were invited to take part in the study though media campaigns and outreach through antenatal and maternity services (Boyd et al., 2013; Fraser et al., 2013). 20,248 pregnancies were identified as being eligible and the initial number of pregnancies enrolled was 14,541. Of the initial pregnancies, there was a total of 14,676 foetuses, resulting in 14,062 live births and 13,988 children who were alive at 1 year of age. When the oldest children were approximately 7 years of age, an attempt was made to bolster the initial sample with eligible cases who had failed to join the study originally. As a result, when considering variables collected from the age of seven onwards (and potentially abstracted from obstetric notes) there are data available for more than the 14,541 pregnancies mentioned above: The number of new pregnancies not in the initial sample (known as Phase I enrolment) that are currently represented in the released data and reflecting enrolment status at the age of 24 is 906, resulting in an additional 913 children being enrolled (456, 262 and 195 recruited during Phases II, III and IV respectively). The phases of enrolment are described in more detail in the cohort profile paper and its update (Boyd et al., 2013; Fraser et al., 2013). The total sample size for analyses using any data collected after the age of seven is therefore 15,447 pregnancies, resulting in 15,658 foetuses. Of these 14,901 children were alive at 1 year of age. Of the original 14,541 initial pregnancies, 338 were from a woman who had already enrolled with a previous pregnancy, meaning 14,203 unique mothers were initially enrolled in the study. As a result of the additional phases of recruitment, a further 630 women who did not enrol originally have provided data since their child was 7 years of age. This provides a total of 14,833 unique women (G0 mothers) enrolled in ALSPAC as of September 2021. G0 partners were invited to complete questionnaires by the mothers at the start of the study and they were not formally enrolled at that time. 12,113 G0 partners have been in contact with the study by providing data and/or formally enrolling when this started in 2010. 3,807 G0 partners are currently enrolled (Northstone et al., 2023).

The study website contains details of all the data that is available through a fully searchable data dictionary and variable search tool (<http://www.bristol.ac.uk/alspac/researchers/our-data/>).

Table S1a: Study definitions of each type of adverse childhood experience and informant

|  | ALSPAC | | MCS | | E-Risk Study | |
| --- | --- | --- | --- | --- | --- | --- |
|  | Definition | Informant | Definition | Informant | Definition | Informant |
| Physical abuse | Partner/mother was physically cruel to child.  Adult in family pushed, grabbed, shoved/smacked to discipline child. People in child's family hit them so hard that it left them with bruises or marks. | Mother, partner, child | Frequency of which respondent smacks child when naughty. | Mother, partner | Child had been intentionally (physically) harmed by an adult. This was ascertained prospectively from interviews with parents using the Multisite Child Development Project interview, recorded debriefings with research workers who had witnessed physical abuse at any of the successive home visits, and information from clinicians whenever the study team made a child-protection referral. Retrospective ascertainment using the Childhood Trauma Questionnaire completed by study members at age 18. | Mother, child, researcher’s home environment observations |
| Sexual abuse | When growing up someone molested child (sexually).  Touched in a sexual way by adult or older child, or was forced to touch adult or older child in a sexual way. Adult or older child forced, or attempted to force, child into any sexual activity by threatening or holding child down or hurting child in some way. | Mother, child | -- | -- | Child had been sexually abused by an adult. This was ascertained prospectively from interviews with parents using the Multisite Child Development Project interview, recorded debriefings with research workers who had witnessed sexual touching/assault at any of the successive home visits, and information from clinicians whenever the study team made a child-protection referral. Retrospective ascertainment using the Childhood Trauma Questionnaire completed by study members at age 18. | Researcher’s home observations, mother, child |
| Emotional abuse | Partner/mother emotionally cruel to child. Adult in family shouted/ said hurtful or insulting things. | Mother, partner, child | -- | -- | Emotional abuse and neglect:  Information on whether emotional abuse had taken place was coded from E-Risk research workers’ narratives of home visits and information from clinicians whenever the study team made a child-protection referral. Observations of parents’ behaviour during home visits: parents screaming and swearing at the children, repeatedly speaking abusively to the children in front of the research workers. Researchers observed the family environment for evidence of emotional neglect using the Home Observation for Measurement of the Environment (HOME). Retrospective ascertainment using the Childhood Trauma Questionnaire completed by study members at age 18. | Researcher’s home environment observations |
| Physical neglect | -- | -- | -- | -- | Researchers observed the family environment for evidence of physical neglect using the Home Observation for Measurement of the Environment (HOME). Any sign the caregiver was not providing a safe, sanitary, or healthy environment for the child. This included the child not having proper clothing or food, as well as grossly unsanitary home environments. Retrospective ascertainment using the Childhood Trauma Questionnaire completed by study members at age 18. | Researcher’s home environment observations |
| Emotional neglect | Carer knows who friends are.  Carer asks/starts conversation about free time/ what happened at school.  Carer takes time to listen when teenager talks about what happened in free time.  Discuss problems with anyone in their family.  Parent/carer talked about child’s experiences at school/ friends/ things that are troubling. Child feels left out of things.  Understood by parents.  When growing up there was someone to take respondent to the doctor if needed.  Someone in family made child feel important or special.  Carer knows what child does with other children. | Child | -- | -- | (See emotional abuse.) |  |
| Bullying | Personal belongings stolen, threatened/blackmailed, hit/beaten up.  Do something didn't want to, told lies about child.  Friends tried to get teenager to do things didn’t want to / told lies about teenager.  Young person has been directly/relationally bullied. Child has been bullied.  Upset by name calling/exclusion from groups or bullying.  Someone threatened/blackmailed teenager. | Child | Cohort member picked on or bullied by other children/  Cohort member bullied at school/ How often do other children bully cohort member | Mother, partner, child, teacher | Bullying by peers: When another child says mean and hurtful things, makes fun, or calls a person mean and hurtful names; completely ignores or excludes someone from their group of friends or leaves them out on purpose; hits, kicks, or shoves a person, or locks them in a room; tells lies or spreads rumors about them; and other hurtful things. Defined as bullying when these things happen often, and when it is difficult to make it stop. Not bullying when it is done in a friendly or playful way. | Mother, child |
| Domestic violence | Physically cruel.  Aware of and affected by one ‘parent’ slapping, kicking, hitting or otherwise physically hurting the other. Kicked, bitten or hit each other, twisted arm, throw body, beaten each other up, choke or strangle each other.  Threatened or used knife or other weapon on each other. | Mother, partner | Partner ever used force in relationship (includes grabbing, pushing, shaking, hitting, kicking etc). | Mother, partner | Mothers reported about perpetration of and victimisation involving 12 forms of physical violence (e.g., slapping, hitting, kicking, and strangling) from the Conflict Tactics Scale. Reports of multiple instances of either perpetration or victimisation constituted physical domestic violence. | Mother |
| Substance use | Smoked cannabis.  Hard drug use or addiction (crack, heroin, amphetamine, opiate, cocaine, methadone, meth).  Alcoholism/drink problem. Alcohol Use Disorders Identification Test (AUDIT). | Mother, partner | Regular use of recreational drugs in the past 12 months (e.g. cannabis, cocaine, ecstasy). Usual frequency of alcohol consumption. | Mother, partner | Mother or father has a history of problems with alcohol or drugs | Mother |
| Parental mental health problems | Parent hurt themselves on purpose or attempted suicide.  Taken medication for depression or anxiety.  Edinburgh Postnatal Depression Scale (EPDS). Diagnosis of schizophrenia, bulimia, anorexia nervosa.  Hospital admission for psychiatric or mental health problems. | Mother, partner | Kessler 6 (K6) scale, Rutter Malaise Inventory (RMI) | Mother, partner | Mother or father has a history of any mental health problem, hospitalisation for a psychiatric disorder, or attempted or completed suicide | Mother |
| Parental conviction | Court conviction/convicted of an offense. | Mother, partner | -- | -- | Mother’s and father’s anti-social behaviour over their life-time including intrusive, aggressive and delinquent behaviours, as well as symptoms of DSM-IV conduct disorder and anti-social personality disorder. | Mother |
| Parental separation | Separated/divorced. | Mother, partner | Legally separated/divorced. | Mother, partner | Biological mother/father was absent from the household at some-point during childhood | Mother |
| The Avon Longitudinal Study of Parents and Children (ALSPAC); The Millennium Cohort Study (MCS); The Environmental Risk (E-Risk) Longitudinal Twin Study. | | | | | | |

Table S1b: Time-point (age of cohort child) and data collection approach (prospective vs retrospective) for the assessment of each adverse childhood experience

|  | ALSPAC | | MCS | | E-Risk | |
| --- | --- | --- | --- | --- | --- | --- |
|  | Retrospective vs prospective measure | Ages measure assessed | Retrospective vs prospective measure | Ages measure assessed | Retrospective vs prospective measure | Ages measure assessed |
| Physical abuse | Both | 8 months, 1, 2, 3, 5, 6, 9, 11, 22 years | Prospective | 3, 5, 7 years | Prospective  + Retrospective | 5, 7, 10, 12 years  18 years |
| Sexual abuse | Both | 1, 2, 3, 5, 6, 7, 9, 22 years | - | - | Prospective  + Retrospective | 5, 7, 10, 12 years  18 years |
| Emotional abuse | Both | 8 weeks, 8 months, 1, 2, 3, 5, 6, 9, 11, 22 years | - | - | Prospective  + Retrospective | 5, 7, 10, 12 years  18 years |
| Physical neglect | - | - | - | - | Prospective  + Retrospective | 5, 7, 10, 12 years  18 years |
| Emotional neglect | Both | 8, 9, 12, 22 years | - | - | Prospective  + Retrospective | 5, 7, 10, 12 years  18 years |
| Bullying | Prospective | 8, 8.5, 10, 12 years | Prospective | 3, 5, 7, 11 years | Prospective | 5, 7, 10, 12 years |
| Domestic violence | Both | 8 months, 1, 2, 3, 5, 6, 8, 9, 11 years | Prospective | 9 months, 3, 5, 7, 11 years | Prospective | 5, 7, 10 years |
| Substance use | Prospective | 8 weeks, 8 months, 1, 2, 3, 5, 6, 8, 9, 11, 12 years | Prospective | 9 months, 3, 5, 7, 11 years | Prospective | 12 years |
| Parental mental health problems | Both | 8 weeks, 8 months, 1, 2, 3, 5, 6, 8, 9, 10, 11, 12 years | Prospective | 9 months, 3, 5, 7, 11 years | Prospective | 12 years |
| Parental conviction (or parental antisocial behaviour in E-Risk) | Both | 8 weeks, 8 months, 1, 2, 3, 5, 6, 9, 11, 12 years | - | - | Prospective | 5 years |
| Parental separation | Both | 8 months, 1, 2, 3, 5, 6, 9, 11, 12 years | Prospective | 9 months, 3, 5, 7, 11 years | Prospective | 5, 7, 10 years |
| The Avon Longitudinal Study of Parents and Children (ALSPAC); The Millennium Cohort Study (MCS); The Environmental Risk (E-Risk) Longitudinal Twin Study. | | | | | | |

Table S2: Millennium Cohort Study (MCS) derivation of dichotomous adverse childhood experiences variables

A modified version of the Rutter Malaise Inventory (RMI) and Kessler 6 (K6) scale were used to assess parental mental health at different time points, a cut-off point of ≥4 was used for the RMI and ≥7 for the K6 scale to estimate prevalence of mental illness (Centre for Longitudinal Studies, 2015; Kessler et al., 2002; Mensah & Kiernan, 2010; Moore, Jayaweera, Redshaw, & Quigley, 2019). A dichotomous variable (yes/no) for each adverse experience was derived using questions indicating exposure to different types of adverse childhood experiences between 9 months and 11 years. The caregiver/partner sample was restricted to questionnaires completed by parents (biological mother/father, adoptive parents, step-parent/partner of parent).

| Adverse childhood experience | Questionnaire | Variable type/response categories | Coding |
| --- | --- | --- | --- |
| Parental mental health problems | Rutter Malaise Inventory:  1. Do you feel tired most of the time?  2. Do you often feel miserable or depressed?  3. Do you often get worried about things?  4. Do you often get into a violent rage?  5. Do you often suddenly become scared for no good reason?  6. Are you easily upset or irritated?  7. Are you constantly keyed up and jittery?  8. Does every little thing get on your nerves and wear you out?  9. Does your heart often race like mad? | Continuous variable (Total score 0-9)  Response –  Yes=1  No=0 | Score <4 (No=0)  Score ≥4 (Yes=1) |
|  | Kessler 6 (K6) scale:  1. During the last 30 days, about how often did you feel so depressed that nothing could cheer you up?  2. During the last 30 days, about how often did you feel hopeless?  3. During the last 30 days, about how often did you feel restless or fidgety?  4. During the last 30 days, about how often did you feel that everything was an effort?  5. During the last 30 days, about how often did you feel worthless?  6. During the last 30 days, about how often did you feel nervous? | Continuous variable: Likert scale (Total score 0-24)  Response –  None of the time=0  A little of the time=1  Some of the time=2  Most of the time=3  All of the time=4 | Score <7 (No=0)  Score ≥7 (Yes=1)  Both the RMI and K6 scale were used to create a dichotomous parental mental health problems variable:  Presence of parental mental health problems coded as ‘Yes=1’ if any parent in a household scored >=4 on the RMI and/or >=7 on the K6 scale.  Scores below these cut-off points on both scales for both parents were coded as ‘No=0’ – indicating the absence of mental health problems. |
| Domestic violence | Partner ever used force in relationship (includes grabbing, pushing, shaking, hitting, kicking etc) | Binary –  Yes  No | No=0  Yes=1 |
| Separation/divorce | Current legal marital status | Categorical –  Divorced  Legally separated  Married: 1^st^ or 2^nd^/later marriage  Single: never married  Widowed  Civil partner | Divorced; Legally separated (Yes=1)  Married: 1^st^ or 2^nd^/ later marriage; Single never married; Widowed; Civil partner (No=0) |
| Substance abuse | Frequency of alcohol consumption | Categorical –  Everyday  5-6 times a week  3-4 times per week  1-2 times per week  1-2 times per month  Never | Everyday; 5-6 times per week (Yes=1)  3-4 per week; 1-2 per week; 1-2 per month; never (No=0) |
|  | Used recreational drugs (ever)/  Used recreational drugs in the past 12 months | Categorical -  Occasionally  Regularly  Never | Regularly (Yes=1),  Occasionally/ Never (No=0)  A dichotomous variable combining frequency of alcohol consumption and recreational drug use created: any parent within a household that consumed alcohol everyday or 5-6 times a week and/or had also used drugs regularly were coded as ‘Yes=1’, which indicates substance use.  A ‘No=0’ on both frequency of alcohol consumption and recreational drug use for all parents in a household indicates no problems with substance use. |
| Physical abuse | How often smacks child when naughty | Categorical –  Never  Rarely  Sometimes (about once a month)  Often (about once a week or more)  Daily  Can’t say | Daily; Often (Yes=1)  Sometimes; Rarely; Never (No=0) |
| Bullying | Cohort member picked on or bullied by other children | Categorical –  Certainly true  Somewhat true  Not true | Certainly true; Many times; Several times; All of the time; Most days; About once a week (Yes=1)  Not true; Somewhat true; Never; Once or twice; Some of the time; About once a month; Every few months; Less often; Can’t say (No=0) |
|  | How often do other children bully you | Categorical –  All of the time  Some of the time  Never |  |
|  | How often do other children hurt you or pick on you on purpose | Categorical –  Most days  About once a week  About once a month  Every few months  Less often  Never |  |
|  | Cohort member bullied at school | Categorical –  Many times  Several times  Once or twice  Never |  |

Figure S1: The Millennium Cohort Study (MCS) study sample selection flowchart

Total sample at MCS 3 (study exposure period)

15,246 families, 15,460 cohort children

Total sample at MCS 1 (study exposure period)

18,552 families, 18,818 cohort children

Additional eligible families recruited at MCS 2

692

Total sample at MCS 2 (study exposure period)

15,590 families, 15,808 cohort children

Total sample at MCS 4 (study exposure period)

13,857 families, 14,043 cohort children

Total sample at MCS 6 (outcome period)

11,872 cohort children

Total sample at MCS 5 (study exposure period)

13,287 families, 13,469 cohort children

The total number of participants excluded n=864 did not meet the following inclusion criteria:

Completion of both self-harm and the Short Mood and Feelings Questionnaire items, in cases where multiple children were eligible, only the first child was included (including the first of twins), inclusion required that a biological or adoptive parent completed at least one sweep of the study between MCS 1 and MCS 5.

Final study sample

11,008 cohort children

Excluded cohort children that did not take part in MCS 6 sweep

1,597

Appendix S2: Measures of adverse childhood experiences in the E-Risk study.

Prospective measures of ten different types of adverse childhood experiences were taken via four home visits at ages 5, 7, 10, and 12 years. Structured notes from interviews with parents using the Multisite Child Development Project interview, recorded debriefings with research workers who had coded any indication of abuse and neglect at any of the successive home visits (including using the Home Observation for Measurement of the Environment), and information from clinicians whenever the study team made a child-protection referral were combined into dossiers to ascertain exposure to childhood maltreatment, the Conflict Tactics Scale assessed household partner violence, the Young Adult Behaviour Checklist assessed parental antisocial behaviour, and the Family History Screen assessed household substance abuse and family history of mental illness (Bradley & Caldwell, 1977; Lansford et al., 2002; Straus, 1979; Weissman et al., 2000). Bullying victimisation was assessed through semi-structured interviews with mothers and separate interviews with the twins. A dichotomous variable was derived to indicate exposure to each type of adverse childhood experience. Any instance of minor or severe physical, emotional, sexual abuse, physical or emotional neglect; occasional or frequent instances of bullying victimisation; single and repeat instances of domestic violence; whether the mother or father of the twins ever had problems with drugs or alcohol; whether the mother or father ever had a mental health problem, were hospitalised for any psychiatric disorder, or attempted suicide, were coded as being exposed. Parental antisocial behaviour included the top 25% of the variety of antisocial behaviours of the mother or father, and parental separation/divorce if any biological parent was absent from the household at any point.

In addition to the prospective measures describe above, the study retrospectively assessed five categories of childhood maltreatment (emotional, physical, and sexual abuse, and emotional and physical neglect) using the 28-item Childhood Trauma Questionnaire (CTQ) at the age-18 follow-up interviews with the twins (Danese et al., 2017). The validity of the CTQ has been demonstrated in both clinical and community samples, the CTQ subscales have test-retest reliability coefficients ranging from. 79 to. 86, and internal consistency coefficients ranging from. 66 to. 92 (Bernstein et al., 1994; Bernstein et al., 2003; Scher, Stein, Asmundson, McCreary, & Forde, 2001). Each subscale is represented by five questions with a score range from 5 to 25; scores fall into four categories: none to low trauma exposure, low to moderate trauma exposure, moderate to severe trauma exposure and severe to extreme trauma exposure for each scale. Responses are measured on a 5-point Likert scale (1 = never true, 2 = rarely true, 3 = sometimes true, 4 = often true, 5 = very often true). Based on previous score recommendations, the study considered a specific category of maltreatment present if the child had a moderate to severe score (Danese et al., 2017).

These retrospective reports were not used by the E-Risk study to derive the core ACE measures describe above, instead, were used to create separate variables related to the five types of abuse for use in the sensitivity analyses.

Table S3: Data availability by time-point, for each adverse childhood experience in ALSPAC and MCS.

To examine the impact of timing and duration of exposure, analyses were restricted to ALSPAC and MCS cohorts as both had sufficient data on ACEs in early (0 to 5 years) and mid childhood (6 to 10 years), and adolescence (11 to 13 years) to enable comparison across the three developmental stages. These age bands were chosen based on key stages for physical and developmental changes, as well as the ages for preschool and primary and secondary education in the UK. For the MCS the age bands selected were as close as possible to those in ALSPAC to enable replication. MCS: early childhood (9 months-5 years), middle childhood (7 years), and adolescence (11 years). Data were available for the following ACEs (see below) for all three developmental stages: ALSPAC: physical abuse, emotional abuse, separation/divorce, parental mental health problems, domestic violence, parental conviction. MCS: parental mental health problems, substance abuse, domestic violence, separation/divorce, bullying.

|  | ALSPAC | | | MCS | | |
| --- | --- | --- | --- | --- | --- | --- |
| Adverse childhood experience | Early childhood (0-5 years) | Middle childhood (6-10 years) | Adolescence (11-13 years) | Early childhood (9 months-5 years) | Middle childhood (7 years) | Adolescence (11 years) |
| Physical abuse | **✓** | **✓** | **✓** | **✓** | **✓** | -- |
| Sexual abuse | **✓** | **✓** | -- | -- | -- | -- |
| Emotional abuse | **✓** | **✓** | **✓** | **--** | -- | -- |
| Emotional neglect | -- | **✓** | **✓** | -- | -- | -- |
| Bullying | -- | **✓** | **✓** | **✓** | **✓** | **✓** |
| Domestic violence | **✓** | **✓** | **✓** | **✓** | **✓** | **✓** |
| Substance abuse | **✓** | **✓** | **✓** | **✓** | **✓** | **✓** |
| Parental mental health problems | **✓** | **✓** | **✓** | **✓** | **✓** | **✓** |
| Parental conviction | **✓** | **✓** | **✓** | -- | -- | -- |
| Separation/divorce | **✓** | **✓** | **✓** | **✓** | **✓** | **✓** |
| The Avon Longitudinal Study of Parents and Children (ALSPAC); The Millennium Cohort Study (MCS) | | | | | | |

Appendix S3: Measures of self-harm among each cohort.

Self-harm in ALSPAC was assessed using the following questions from the ‘Life of a 16+ Teenager’ postal questionnaire: for lifetime self-harm up to age 16 “Have you ever hurt yourself in any way (e.g., by taking an overdose of pills, or by cutting yourself)?” and past year self-harm “How many times have you done this in the last year?”, followed by “When was the last time you hurt yourself on purpose?”. In the MCS past year self-harm was assessed with the following question from sweep 6 (MCS 6) of the study, when the children we aged 14 years: “In the past year have you hurt yourself on purpose in any way?”. In the E-Risk study the twins were interviewed at age 18 and were asked the following questions: “Since you were 12 years old, when we last saw you: Have you ever tried to kill yourself? Attempted suicide? Have you ever tried to hurt yourself, to cope with stress or emotional pain?”.

Appendix S4: Covariates

ALSPAC:

- Household social class at 18 weeks gestation – highest of mother and partners social class according to the Registrar General’s Social Classes: (professional/managerial and technical/skilled nonmanual/partly skilled/unskilled),
- parity at 18 weeks gestation (continuous),
- mother’s home ownership status during pregnancy (owned or mortgaged/rented/other),
- mother’s highest educational qualification during pregnancy (CSE/vocational/O level/A level/degree level),
- mother’s age at delivery (under 20/20-29/30 plus).

MCS:

- Household income at MCS 1 (child aged 9 months) – Equivalized (taking into account the number and age of adults and dependents in the household) family income obtained by dividing total net weekly household income by the number of household members according their assigned weight (1 for first adult, 0.5 for each remaining adult, and 0.3 for each child under 14 years) on the Organisation for Economic Co-operation and Development (OECD)’s equivalised income scale (lowest quintile/second quintile/third quintile/fourth quintile/highest quintile),
- mother’s ethnicity (White/Mixed/Indian/Bangladeshi, Pakistani/Black or Black British/Other),
- child’s ethnicity (White/Mixed/Indian/Bangladeshi, Pakistani/Black or Black British/Other),
- family housing tenure (owned or mortgaged/rented/other),
- mother’s age at delivery (under 20/20-29/30-39/40 plus),
- mother’s highest educational qualification at MCS 1 (NVQ level 1/NVQ level 2/NVQ level 3/NVQ level 4/NVQ level 5/overseas qualifications only/none of these).

E-Risk:

- family socioeconomic position when children were aged 5 years – composite measure based on highest parental income, education, and occupation (low/middle/high),
- mother’s age at delivery (under 20/20-29/30 plus),
- mother’s highest educational qualification at age 5 years (CSE/AS or A level/HNC or HND/degree level/none),
- child’s biological sex at birth (male/female).

Appendix S5: Multiple imputation strategy for ALSPAC and MCS.

In the ALSPAC data there were 385 variables identified that measured the ten types of childhood adversity from birth to 13 years, there was also a high proportion of missing data among these variables due to selective attrition of high risk families that are more likely to experience adverse childhood experiences and are also from lower social classes (Houtepen, Heron, Suderman, Tilling, & Howe, 2018). For the outcomes, postal questionnaires at age 16 were sent to 9,937 (70%) of the original live born children in the study, 5,108 (51%) of these children completed and returned the questionnaires, which is 36% of the 14,062 live born children. Of the completed questionnaires, there were 4,860 adolescents that completed questions on both the outcomes of interest. Therefore a complete-case analytic approach would result in biased estimates and low statistical power to detect significant effects (n= 2,234 for a complete-case analysis). A binary (yes/no) variable was derived for individuals that had completed at least 50% of questions relating to each adverse childhood experience between 0-13 years (exposure period). This was recorded as missing if over 50% of the questions for each adversity had missing data; these values were imputed using multiple imputation by chained equations (MICE) based on the assumption that data were missing-at-random (Azur, Stuart, Frangakis, & Leaf, 2011; Houtepen et al., 2018; White, Royston, & Wood, 2011). Missing values for self-harm and depression, and confounders were also imputed up to the total number of postal questionnaires sent to adolescents at age 16, and who also had data on over 50% of the questions for each adverse childhood experience. Missing values were imputed separately by sex to preserve interactions. Multiple imputation is one way of handling missing data, the procedure ‘imputes’ or fills in missing values multiple times based on observed data, creating multiple complete datasets through an iterative process, the parameter estimates are then pooled using Rubin’s rules (Azur et al., 2011). MICE operates under the assumption that data are missing-at-random – that the probability of a missing value depends on observed data and are not related to the missing data (Azur et al., 2011). In this study a total of 50 imputed datasets were generated, the imputation model included all the variables in the analytic model including the exposures, outcomes, covariates, and two types of auxiliary variables that make the missing-at-random assumption more plausible. Auxiliary variables associated with missingness and adverse childhood experiences included for example sociodemographic characteristics, measures of adversity before the birth of the study child, or when the study child was between 18 to 21 years old. Variables related to the outcomes such as earlier and later measures of depression and self-harm, emotional and behavioural problems, substance abuse, and child IQ were also included in the imputation model.

In the MCS, of the initial 18,552 families recruited in the study at the first sweep, 8,896 (48%) participated in all of the first 5 sweeps (exposure period for this study), and the study child in sweep 6 at age 14 years. Of those, 4,649 (52%) had complete data for exposure, confounder and outcome variables (complete-cases). Those with missing data were more likely to have lower household income and were less likely to own/mortgage their home, mothers were younger in age at the birth of the study child, and they were also less likely to have higher qualifications. Therefore, a complete-case analysis would produce biased estimates. A dichotomous variable was derived for each type of adverse childhood experience combining data from the primary caregiver, the partner, and child at each time point of data collection, missing values were then imputed up the number of children that had data on both self-harm and depression at age 14 (n=11,008), missing data among confounders were also imputed. Fifty imputed datasets were generated, the imputation model included all the variables in the analytic model including the outcomes, exposures, study design features (stratification characteristics - disadvantaged and minority ethnic stratum within country) and additional auxiliary variables that predict missingness and make the missing-at-random assumption plausible.

Table S4: Description of the life-course models tested using the Structured Lifecourse Modelling Approach (SLCMA)

The lasso selects the hypothesis (represented by an exposure) with the strongest association with the outcome first, corresponding to explaining the greatest proportion of variance in the outcome (see Table below). Additional encoded variables were added in order of strength of association with outcome variation. Elbow plots of the proportion of outcome variation explained by the lasso fit (McFadden’s pseudo R^2^) against the number of variables selected during the lasso procedure were produced. The elbow, represented by a point at which adding additional variables does not substantively increase the pseudo R^2^ values, is the point used to choose the best-fitting life-course hypothesis. Due to the large number of lasso models considered, we restricted our reporting to the model indicated by the first variable selected by lasso (i.e. the simplest hypothesis explaining the greatest variation in the outcome). Elbow plots showing additional hypotheses selected by lasso are presented in Figures S2a and S2b. Once the best-fitting hypothesis for the data was selected, odds ratios, p-values and confidence intervals were estimated to interpret the results of the hypotheses in relation to the outcomes, using Bonferroni correction to p-values to account for having selected the best-fitting hypothesis. The SLCMA requires the exposure to the same ACE to be measured during all three potential critical periods.

| Hypothesis | Estimate | Encoding variables |
| --- | --- | --- |
| Critical period | Proportion of outcome variation explained by the lasso fit (McFadden’s pseudo R^2^) by the period during which the ACE had a strong association with the outcome (self-harm, depression, co-occurring self-harm and depression).  Critical period 1: Early childhood (between birth and 5 years)  Critical period 2: Middle childhood (between 6 and 10 years)  Critical period 3: Early adolescence (between 11 and 13 years) | Pseudo R^2^ explained by three binary variables for each ACE:  Parental mental health problems –  Pmh_1 = exposed vs unexposed  Pmh_2 = exposed vs unexposed  Phm_3 = exposed vs unexposed  Domestic violence –  Dv_1 = exposed vs unexposed  Dv_2 = exposed vs unexposed  Dv_3 = exposed vs unexposed  Physical abuse –  Pa_1 = exposed vs unexposed  Pa_2 = exposed vs unexposed  Pa_3 = exposed vs unexposed  Emotional abuse –  Ea_1 = exposed vs unexposed  Ea_2 = exposed vs unexposed  Ea_3 = exposed vs unexposed  Bullying –  Bullied_1 = exposed vs unexposed  Bullied_2 = exposed vs unexposed  Bullied_3 = exposed vs unexposed  Substance abuse –  Suba_1 = exposed vs unexposed  Suba_2 = exposed vs unexposed  Suba_3 = exposed vs unexposed  Separation/divorce –  Div_1 = exposed vs unexposed  Div_2 = exposed vs unexposed  Div_3 = exposed vs unexposed  Parental conviction –  Pc_1 = exposed vs unexposed  Pc_2 = exposed vs unexposed  Pc_3 = exposed vs unexposed |
| Accumulation of risk | Proportion of outcome variation explained by the lasso fit (McFadden’s pseudo R^2^) by the number of times a person is exposed to each ACE in early childhood, middle childhood, and early adolescence. | Pseudo R^2^ explained by one binary variable for each ACE:  Parental mental health problems –  Pmh_accum = Pmh_1 + Pmh_2 + Pmh_3  Domestic violence –  Dv_accum = Dv_1 + Dv_2 + Dv_3  Physical abuse –  Pa_accum = Pa_1 + Pa_2 + Pa_3  Emotional abuse –  Ea_accum = Ea_1 + Ea_2 + Ea_3  Bullying –  Bullied_accum = Bullied_1 + Bullied_2 + Bullied_3  Substance abuse –  Suba_accum = Suba_1 + Suba_2 + Suba_3  Separation/divorce –  Div_accum = Div_1 + Div_2 + Div_3  Parental conviction –  Pc_accum = Pc_1 + Pc_2 + Pc_3 |

Table S5: Prevalence of adverse childhood experiences, self-harm and depression, and confounders in multiply imputed and complete-case data in ALSPAC

|  | Pooled proportions from multiply imputed data (n=9,511) | Complete-case proportions (n=2,234) |
| --- | --- | --- |
| *Adverse childhood experience (birth-13 years)* | | |
| Parental mental health problems | 45.9 | 38.0 |
| Domestic violence | 24.2 | 15.5 |
| Substance abuse | 14.5 | 7.6 |
| Bullying | 15.9 | 13.7 |
| Physical abuse | 15.1 | 11.7 |
| Sexual abuse | 3.5 | 2.8 |
| Emotional abuse | 24.4 | 16.6 |
| Emotional neglect | 18.3 | 15.1 |
| Separation/divorce | 29.9 | 16.0 |
| Parental conviction | 8.8 | 6.4 |
| *Outcomes* | | |
| Neither self-harm or depression | 60.5 | 80.6 |
| Self-harm alone | 11.1 | 6.1 |
| Depression alone | 10.4 | 9.6 |
| Co-occurring self-harm and depression | 18.0 | 3.7 |
| *Confounders* | | |
| *Sex* | | |
| Male | 49.2 | 43.2 |
| Female | 50.8 | 56.8 |
| *Mother’s age at delivery* | | |
| Under 20 years | 2.6 | 0.4 |
| 20 to 29 years | 55.7 | 45.8 |
| 30 plus years | 41.7 | 53.8 |
| *Family housing tenure* |  |  |
| Owned/mortgaged | 76.3 | 90.2 |
| Rented | 20.0 | 7.8 |
| Other | 3.8 | 2.0 |
| *Mother’s qualifications* |  |  |
| CSE | 16.4 | 7.5 |
| Vocational | 9.8 | 5.8 |
| O level | 34.0 | 32.3 |
| A level | 24.6 | 31.0 |
| Degree level | 15.2 | 23.4 |
| *Household social class* |  |  |
| Professional | 11.1 | 16.4 |
| Managerial and technical | 37.6 | 47.8 |
| Skilled non-manual | 29.1 | 25.9 |
| Skilled manual | 11.9 | 6.9 |
| Partly skilled/unskilled | 10.3 | 3.0 |
| Parity (mean) | 0.82 | 0.83 |

Table S6: Prevalence of adverse childhood experiences, self-harm and depression, and confounders in multiply imputed and complete-case data in the MCS

|  | Pooled proportions from multiply imputed data (n= 11,008) | Complete-case proportions (n=4,649) |
| --- | --- | --- |
| *Adverse childhood experience (9 months-11 years)* | | |
| Parental mental health problems | 53.0 | 41.5 |
| Domestic violence | 27.3 | 21.6 |
| Substance abuse | 30.6 | 36.8 |
| Bullying | 40.4 | 31.4 |
| Physical abuse | 25.2 | 22.0 |
| Separation/divorce | 27.9 | 13.5 |
| *Outcomes* | | |
| Neither self-harm or depression | 78.1 | 79.0 |
| Self-harm alone | 6.5 | 6.6 |
| Depression alone | 7.3 | 6.5 |
| Co-occurring self-harm and depression | 8.1 | 7.9 |
| *Confounders* | | |
| *Mother’s ethnicity* | | |
| White | 82.0 | 93.6 |
| Mixed | 0.3 | 0.4 |
| Indian | 0.3 | 1.9 |
| Pakistani/Bangladeshi | 7.5 | 2.0 |
| Black/Black British | 3.3 | 1.0 |
| Other ethnic group | 1.5 | 1.0 |
| *Mother’s age at delivery* | | |
| Under 20 years | 6.7 | 1.4 |
| 20 to 29 years | 44.4 | 37.5 |
| 30 to 39 years | 46.4 | 58.2 |
| 40 plus years | 2.4 | 2.8 |
| *Mother’s qualifications* | | |
| NVQ level 1 | 9.8 | 7.1 |
| NVQ level 2 | 32.5 | 31.2 |
| NVQ level 3 | 10.0 | 12.3 |
| NVQ level 4 | 25.0 | 36.2 |
| NVQ level 5 | 4.1 | 6.0 |
| Overseas qualifications only | 3.0 | 1.4 |
| None of these | 15.7 | 5.6 |
| *Family housing tenure* | | |
| Owned/mortgaged | 64.1 | 85.1 |
| Rented | 30.1 | 12.3 |
| Other | 5.8 | 2.6 |
| *Household income* | | |
| Quintile 1 (lowest) | 20.8 | 4.7 |
| Quintile 2 | 21.1 | 14.8 |
| Quintile 3 | 19.2 | 22.2 |
| Quintile 4 | 19.9 | 28.2 |
| Quintile 5 (highest) | 18.9 | 30.1 |
| *Child’s sex* | | |
| Male | 49.7 | 48.7 |
| Female | 50.3 | 51.3 |
| *Child’s ethnicity* | | |
| White | 82.0 | 92.8 |
| Mixed | 2.8 | 1.8 |
| Indian | 2.8 | 1.8 |
| Pakistani/Bangladeshi | 7.5 | 2.0 |
| Black/Black British | 3.3 | 0.9 |
| Other ethnic group | 1.5 | 0.6 |

Table S7: Prevalence of adverse childhood experiences, self-harm and depression, and confounders in the E-Risk Study

|  | Prevalence % |
| --- | --- |
| *Adverse childhood experience (birth-12 years)* | |
| Parental mental health problems | 60.4 |
| Domestic violence | 45.8 |
| Substance abuse | 16.2 |
| Parental antisocial behaviour | 25.4 |
| Separation/Divorce | 48.4 |
| Physical abuse | 20.1 |
| Physical neglect | 8.9 |
| Emotional abuse and neglect | 11.6 |
| Sexual abuse | 1.6 |
| Bullying victimisation | 44.8 |
| *Outcomes* | |
| Neither self-harm nor depression | 73.5 |
| Self-harm alone | 6.4 |
| Depression alone | 12.3 |
| Co-occurring self-harm and depression | 7.8 |
| *Confounders* | |
| Male | 48.9 |
| Female | 51.1 |
| *Mother’s age at delivery* | |
| Under 20 | 6.9 |
| 20-29 | 48.8 |
| 30 plus | 44.3 |
| *Mother’s qualifications* | |
| CSE | 53.0 |
| AS/A Levels | 7.3 |
| HNC/HND | 8.6 |
| Degree level | 12.7 |
| None | 18.3 |
| *Household social class* | |
| Low | 33.4 |
| Medium | 33.1 |
| High | 33.4 |

Table S8: Prevalence of adverse childhood experiences and confounders by outcome – pooled proportions from multiply imputed data, ALSPAC

|  | Neither self-harm nor depression % (95% CI) | Self-harm alone % (95% CI) | Depression alone % (95% CI) | Co-occurring self-harm and depression % (95% CI) |
| --- | --- | --- | --- | --- |
| *Adverse childhood Experience* | | | | |
| Parental mental health problems | 42.2 (40.7-43.6) | 47.8 (42.5-53.2) | 54.1 (49.9-58.3) | 52.3 (49.0-56.6) |
| Domestic violence | 21.4 (19.8-23.0) | 27.8 (22.7-32.8) | 25.8 (21.2-30.4) | 30.6 (26.7-34.5) |
| Substance abuse | 11.7 (10.6-12.9) | 15.7 (11.6-19.8) | 18.9 (15.0-22.9) | 20.6 (17.3-23.9) |
| Bullying | 13.4 (12.4-14.5) | 15.9 (12.3-19.5) | 21.1 (17.7-24.5) | 21.2 (18.2-24.2) |
| Physical abuse | 13.2 (11.9-14.4) | 17.5 (13.6-21.4) | 18.4 (14.8-21.9) | 18.5 (15.2-21.9) |
| Sexual abuse | 2.7 (2.1-3.3) | 5.3 (3.1-7.4) | 5.0 (3.1-6.9) | 4.5 (3.0-6.1) |
| Emotional abuse | 20.3 (18.9-21.7) | 25.2 (20.4-30.1) | 33.0 (28.8-37.3) | 32.6 (28.6-36.5) |
| Emotional neglect | 17.4 (15.9-18.9) | 18.7 (15.1-22.3) | 18.6 (14.8-22.4) | 20.9 (17.2-24.6) |
| Separation/divorce | 26.0 (24.5-27.6) | 33.1 (28.1-38.1) | 34.3 (30.0-38.6) | 38.5 (34.2-42.8) |
| Parental conviction | 7.7 (6.7-8.6) | 9.9 (6.7-13.3) | 10.6 (7.6-13.7) | 11.1 (8.6-13.7) |
| *Sex of child* | | | | |
| Male | 45.0 (43.5-46.5) | 55.6 (47.5-63.7) | 29.8 (25.9-33.7) | 70.3 (66.7-73.9) |
| Female | 55.0 (53.5-56.5) | 44.4 (36.3-52.5) | 70.2 (66.3-74.1) | 29.7 (26.1-33.3) |
| *Ethnicity of child* | | | | |
| White | 94.3 (93.5-95.1) | 93.4 (90.7-96.1) | 92.1 (89.6-94.7) | 93.8 (91.7-95.8) |
| Non-white | 5.7 (4.9-6.5) | 6.6 (3.9-9.3) | 7.9 (5.3-10.4) | 6.2 (4.2-8.3) |
| *Mother’s age at delivery* | | | | |
| Under 20 years | 1.9 (1.5-2.3) | 3.8 (2.3-5.4) | 3.5 (1.9-5.1) | 4.0 (2.7-5.3) |
| 20 to 29 years | 53.4 (52.0-54.8) | 59.2 (55.2-63.2) | 55.1 (51.3-58.9) | 61.5 (58.8-64.3) |
| 30 plus years | 44.7 (43.3-46.1) | 37.0 (33.0-41.0) | 41.4 (37.5-45.3) | 34.5 (31.1-37.3) |
| *Family housing tenure* | | | | |
| Owned/mortgaged | 79.5 (78.2-80.8) | 68.3 (63.1-73.5) | 75.8 (71.1-79.9) | 70.6 (66.8-74.3) |
| Rented | 17.0 (15.6-18.3) | 27.1 (21.9-32.3) | 20.5 (16.6-24.3) | 25.4 (21.7-29.2) |
| Other | 3.5 (2.9-4.1) | 4.6 (2.7-6.6) | 3.7 (2.1-5.3) | 4.0 (2.5-5.5) |
| *Mother’s qualifications* | | | | |
| CSE | 13.7 (12.6-14.9) | 19.5 (14.9-24.1) | 19.2 (15.7-22.7) | 21.7 (18.7-24.6) |
| Vocational | 9.0 (8.0-10.0) | 10.1 (7.1-13.2) | 11.4 (8.6-14.2) | 11.4 (8.7-14.2) |
| O level | 32.7 (31.3-34.2) | 32.0 (28.1-36.0) | 36.0 (32.3-39.7) | 38.1 (34.6-41.6) |
| A level | 27.0 (25.7-28.3) | 23.1 (18.9-27.3) | 22.1 (18.7-25.4) | 18.9 (16.3-21.5) |
| Degree level | 17.5 (16.4-18.6) | 15.2 (12.2-18.2) | 11.4 (8.9-13.8) | 9.9 (7.9-12.0) |
| *Household social class* | | | | |
| Professional | 12.9 (11.9-13.9) | 9.1 (6.6-11.5) | 9.4 (7.2-11.6) | 7.4 (5.7-9.1) |
| Managerial and technical | 40.1 (38.6-41.6) | 31.4 (26.9-35.9) | 37.9 (34.0-41.8) | 32.7 (29.5-35.9) |
| Skilled non-manual | 28.1 (26.7-29.4) | 33.3 (29.1-37.5) | 28.1 (24.4-31.8) | 30.8 (27.6-34.0) |
| Skilled manual | 10.4 (9.4-11.4) | 14.7 (10.9-18.6) | 11.9 (9.1-14.7) | 15.3 (12.5-18.0) |
| Partly skilled/unskilled | 8.6 (7.6-9.5) | 11.5 (7.4-15.6) | 12.7 (9.6-15.8) | 13.8 (11.3-16.4) |
| Parity (mean) | 0.76 (0.73-0.79) | 0.90 (0.79-1.01) | 0.84 (0.76-0.92) | 0.95 (0.87-1.02) |

Table S9: Prevalence of adverse childhood experiences and confounders by outcome – pooled proportions from multiply imputed data, MCS

|  | Neither self-harm or depression n= 8,600  % (95% CI) | Self-harm alone n= 714  % (95% CI) | Depression alone n= 801  % (95% CI) | Co-occurring self-harm and depression n= 893  % (95% CI) |
| --- | --- | --- | --- | --- |
| *Adverse childhood Experience* | | | | |
| Parental mental health problems | 51.3 (50.2-52.4) | 55.3 (51.6-59.1) | 60.1 (56.6-63.7) | 60.5 (57.2-63.8) |
| Domestic violence | 26.0 (24.8-27.1) | 31.6 (27.6-35.6) | 30.9 (27.1-34.7) | 33.7 (30.0-37.4) |
| Substance abuse | 30.1 (29.1-31.1) | 33.5 (29.9-37.1) | 29.0 (25.7-32.3) | 34.6 (31.4-37.8) |
| Bullying | 38.3 (37.0-39.7) | 46.2 (42.4-50.1) | 48.5 (44.8-52.2) | 48.6 (45.1-52.1) |
| Physical abuse | 25.2 (24.1-26.3) | 25.2 (21.7-28.7) | 25.8 (22.4-29.1) | 24.8 (21.9-27.8) |
| Separation/divorce | 26.7 (25.8-27.7) | 32.3 (28.8-35.8) | 32.2 (28.9-35.5) | 31.9 (28.7-35.0) |
| *Sex of child* | | | | |
| Male | 55.4 (54.5-56.5) | 35.2 (31.7-38.7) | 34.6 (31.3-37.9) | 19.5 (16.9-22.1) |
| Female | 44.6 (43.5-45.6) | 64.8 (61.3-68.3) | 65.4 (62.1-68.7) | 80.5 (77.9-83.1) |
| *Ethnicity of child* | | | | |
| White | 81.0 (80.2-81.8) | 86.1 (83.6-88.7) | 81.6 (79.0-84.3) | 89.1 (87.1-91.2) |
| Mixed | 2.7 (2.3-3.0) | 3.4 (2.0-4.7) | 3.1 (1.9-4.3) | 3.5 (2.3-4.7) |
| Indian | 2.9 (2.5-3.2) | 1.7 (0.7-2.6) | 3.5 (2.2-4.8) | 1.8 (0.9-2.7) |
| Pakistani/Bangladeshi | 8.1 (7.6-8.7) | 5.2 (3.6-6.8) | 8.2 (6.3-10.1) | 3.2 (2.1-4.4) |
| Black/Black British | 3.7 (3.3-4.0) | 2.7 (1.5-3.8) | 2.2 (1.2-3.3) | 1.2 (0.5-2.0) |
| Other ethnic group | 1.7 (1.4-1.9) | 1.0 (0.3-1.7) | 1.2 (0.5-2.0) | 1.1 (0.4-1.8) |
| *Household income** | | | | |
| Quintile 1 (lowest) | 20.4 (19.5-21.2) | 22.2 (19.1-25.3) | 21.4 (18.5-24.3) | 23.4 (20.6-26.3) |
| Quintile 2 | 20.6 (19.7-21.4) | 21.3 (18.2-24.3) | 25.0 (22.0-28.1) | 23.0 (20.1-25.8) |
| Quintile 3 | 19.3 (18.5-20.1) | 18.5 (15.6-21.4) | 19.9 (17.0-22.7) | 18.2 (15.6-20.8) |
| Quintile 4 | 20.3 (19.4-21.2) | 17.4 (14.6-20.2) | 18.7 (16.0-21.4) | 19.2 (16.6-21.9) |
| Quintile 5 (highest) | 19.5 (18.6-20.3) | 20.6 (17.7-23.6) | 15.0 (13.7-18.6) | 16.1 (13.7-18.6) |
| *Mother’s age at delivery* | | | | |
| Under 20 years | 6.4 (5.9-6.9) | 7.0 (5.1-8.9) | 9.0 (7.0-11.0) | 7.7 (6.0-9.5) |
| 20 to 29 years | 44.2 (43.1-45.2) | 43.7 (40.0-47.4) | 46.7 (43.2-50.2) | 45.6 (42.3-48.9) |
| 30 to 39 years | 47.0 (45.9-48.1) | 46.4 (42.6-50.1) | 42.1 (38.6-45.5) | 44.1 (40.8-47.4) |
| 40 plus years | 2.4 (2.1-2.7) | 2.9 (1.7-4.2) | 2.2 (1.2-3.2) | 2.6 (1.5-3.6) |
| *Mother’s ethnicity* | | | | |
| White | 82.4 (81.5-83.2) | 88.1 (85.7-90.5) | 83.4 (80.8-86.0) | 90.1 (88.1-92.0) |
| Mixed | 0.9 (0.7-1.1) | 0.7 (0.09-1.3) | 0.9 (0.2-1.5) | 1.5 (0.7-2.3) |
| Indian | 3.0 (2.6-3.3) | 2.1 (1.0-3.2) | 3.6 (2.3-4.9) | 1.5 (0.7-2.3) |
| Pakistani/Bangladeshi | 8.0 (7.5-8.6) | 5.0 (3.4-6.6) | 8.1 (6.2-10.0) | 3.7 (2.5-4.9) |
| Black/Black British | 3.7 (3.3-4.1) | 2.9 (1.7-4.2) | 2.4 (1.3-3.4) | 1.5 (0.7-2.3) |
| Other ethnic group | 2.0 (1.7-2.3) | 1.1 (0.3-1.9) | 1.6 (0.8-2.5) | 1.8 (0.9-2.7) |
| *Family housing tenure** | | | | |
| Owned/mortgaged | 65.2 (64.2-66.2) | 63.7 (60.1-67.2) | 59.2 (55.7-62.6) | 58.0 (54.8-61.3) |
| Rented | 28.9 (27.9-29.9) | 31.2 (27.8-34.7) | 34.5 (31.1-37.8) | 36.8 (33.6-40.0) |
| Other | 5.9 (5.4-6.4) | 5.1 (3.5-6.8) | 6.4 (4.6-8.1) | 5.2 (3.6-6.7) |
| *Mother’s qualifications** | | | | |
| NVQ level 1 | 9.5 (8.9-10.2) | 9.8 (7.6-12.0) | 10.1 (8.0-12.3) | 11.7 (9.5-13.8) |
| NVQ level 2 | 32.2 (31.2-33.2) | 34.7 (31.1-38.2) | 31.0 (27.7-34.3) | 34.4 (31.2-37.6) |
| NVQ level 3 | 9.9 (9.3-10.6) | 10.5 (8.3-12.8) | 10.5 (8.3-12.6) | 9.6 (7.6-11.6) |
| NVQ level 4 | 25.4 (24.5-26.4) | 25.2 (22.0-28.4) | 22.9 (19.9-25.8) | 23.0 (20.2-25.8) |
| NVQ level 5 | 4.2 (3.8-4.6) | 3.3 (2.0-4.6) | 4.1 (2.7-5.5) | 4.2 (2.9-5.6) |
| Overseas qualifications only | 3.0 (2.6-3.4) | 3.2 (1.9-4.5) | 3.2 (1.9-4.4) | 2.4 (1.3-3.4) |
| None of these | 15.7 (14.9-16.5) | 13.2 (10.7-15.8) | 18.3 (15.5-21.0) | 14.7 (12.3-17.1) |
| *Reported at sweep 1, child aged 9 months | | | | |

Table S10: Prevalence of adverse childhood experiences and confounders by outcome in the E-Risk study

|  | Neither self-harm nor major depressive disorder N=1,515  n (%) | Self-harm alone  N=132 n (%) | Major depressive disorder alone N=253  n (%) | Co-occurring self-harm and major depressive disorder  N=161  n (%) |
| --- | --- | --- | --- | --- |
| *Adverse childhood experience* | | | | |
| Parental mental health problems | 850 (57.6) | 84 (66.1) | 162 (65.6) | 116 (73.9) |
| Domestic violence | 643 (42.4) | 72 (54.6) | 125 (49.4) | 95 (59.0) |
| Substance abuse | 204 (14.3) | 20 (16.7) | 42 (17.5) | 43 (29.3) |
| Parental antisocial behaviour | 351 (23.3) | 34 (25.8) | 72 (28.5) | 60 (37.3) |
| Separation/Divorce | 683 (46.4) | 68 (53.5) | 124 (50.8) | 86 (54.8) |
| Physical abuse | 273 (18.0) | 38 (28.8) | 60 (23.7) | 43 (26.7) |
| Physical neglect | 119 (7.9) | 20 (15.2) | 22 (8.7) | 23 (14.3) |
| Emotional abuse and neglect | 152 (10.0) | 24 (18.2) | 36 (14.2) | 27 (16.8) |
| Sexual abuse | 14 (0.9) | - | 9 (3.6) | 7 (4.4) |
| Bullying victimisation | 626 (41.4) | 66 (50.4) | 127 (50.2) | 102 (63.4) |
| *Sex of twins* | | | | |
| Males | 772 (51.0) | 45 (34.1) | 103 (40.7) | 57 (35.4) |
| Females | 743 (49.0) | 87 (65.9) | 150 (59.3) | 104 (64.6) |
| *Mother’s age at delivery* | | | | |
| Under 20 | 92 (6.1) | 19 (14.2) | 19 (7.5) | 13 (8.1) |
| 20 to 29 | 723 (47.7) | 62 (47.0) | 132 (52.2) | 88 (54.7) |
| 30 plus | 700 (46.2) | 51 (38.6) | 102 (40.3) | 60 (37.3) |
| *Mother’s qualifications* | | | | |
| CSE (1-5) GCSE (A-G) O Level (A-C) | 795 (52.5) | 67 (50.8) | 146 (57.7) | 84 (52.2) |
| A/AS Levels | 126 (2.3) | 7 (5.3) | 10 (4.0) | 8 (5.0) |
| HNC/HND | 124 (8.2) | 14 (10.6) | 19 (7.5) | 21 (13.0) |
| Degree Level | 218 (14.4) | 9 (6.8) | 23 (9.1) | 12 (7.5) |
| None | 252 (16.6) | 35 (26.5) | 55 (21.7) | 36 (22.4) |
| *Social class* | | | | |
| Low | 470 (31.0) | 56 (42.4) | 96 (37.9) | 67 (41.6) |
| Middle | 501 (33.1) | 45 (34.1) | 84 (33.2) | 53 (32.9) |
| High | 544 (35.9) | 31 (23.5) | 73 (28.9) | 41 (25.5) |
| *Counts less than <5 are not displayed. | | | | |

Table S11: Crude relative risk ratios for the association between adverse childhood experiences and self-harm and depression among each

cohort

|  | **ALSPAC** | | | **MCS** | | | **E-Risk** | | |
| --- | --- | --- | --- | --- | --- | --- | --- | --- | --- |
| Adverse childhood experience | Self-harm alone*  (n= 993) | Depression alone*  (n= 973) | Co-occurring self-harm and depression*  (n= 1,735) | Self-harm alone (n= 714) | Depression alone (n= 801) | Co-occurring self-harm and depression (n= 893) | Self-harm alone (n=132) | Depression alone (n=253) | Co-occurring self-harm and depression (n=161) |
|  | RRR (95% CI) | RRR (95% CI) | RRR (95% CI) | RRR (95% CI) | RRR (95% CI) | RRR (95% CI) | RRR (95% CI) | RRR (95% CI) | RRR (95% CI) |
| Parental mental health problems | 1.26 (1.01-1.57) | 1.62 (1.35-1.93) | 1.53 (1.29-1.82) | 1.25 (1.04-1.51) | 1.46 (1.22-1.74) | 1.60 (1.36-1.90) | 1.44 (0.95-2.18) | 1.40 (1.04-1.89) | 2.08 (1.41-3.08) |
| Domestic violence | 1.41 (1.08-1.84) | 1.27 (0.99-1.65) | 1.62 (1.30-2.02) | 1.37 (1.11-1.69) | 1.24 (0.99-1.55) | 1.43 (1.18-1.73) | 1.63 (1.11-2.38) | 1.32 (1.00-1.76) | 1.95 (1.36-2.80) |
| Substance abuse | 1.39 (1.01-1.93) | 1.75 (1.32-2.33) | 1.94 (1.52-2.49) | 1.21 (1.01-1.45) | 0.98 (0.81-1.18) | 1.12 (0.93-1.35) | 1.20 (0.71-2.02) | 1.27 (0.87-1.85) | 2.47 (1.61-3.79) |
| Bullying | 1.21 (0.91-1.62) | 1.72 (1.37-2.16) | 1.73 (1.40-2.14) | 1.52 (1.26-1.84) | 1.40 (1.15-1.71) | 1.59 (1.36-1.86) | 1.44 (0.99-2.08) | 1.43 (1.09-1.87) | 2.45 (1.73-3.46) |
| Physical abuse | 1.40 (1.05-1.86) | 1.48 (1.14-1.92) | 1.50 (1.16-1.92) | 0.99 (0.81-1.22) | 0.99 (0.80-1.23) | 1.02 (0.85-1.23) | 1.84 (1.19-2.84) | 1.41 (1.01-1.98) | 1.66 (1.11-2.47) |
| Physical neglect | - | - | - | - | - | - | 2.09 (1.19-3.69) | 1.12 (0.70-1.79) | 1.96 (1.10-3.47) |
| Sexual abuse | 1.98 (1.22-3.22) | 1.88 (1.20-2.95) | 1.70 (1.11-2.60) | - | - | - | 1.65 (0.41-6.68) | 3.95 (1.62-9.64) | 4.87 (2.01-11.79) |
| Emotional abuse^+^ | 1.32 (1.02-1.73) | 1.94 (1.57-2.39) | 1.90 (1.54-2.33) | - | - | - | 1.99 (1.23-3.22) | 1.49 (1.00-2.22) | 1.81 (1.09-3.01) |
| Emotional neglect | 1.09 (0.83-1.43) | 1.09 (0.83-1.43) | 1.25 (0.96-1.63) | - | - | - | - | - | - |
| Separation/divorce | 1.40 (1.11-1.77) | 1.48 (1.21-1.82) | 1.78 (1.44-2.19) | 1.24 (1.01-1.51) | 1.43 (1.18-1.73) | 1.35 (1.13-1.61) | 1.33 (0.90-1.96) | 1.19 (0.89-1.59) | 1.40 (0.98-2.00) |
| Parental conviction | 1.31 (0.89-1.94) | 1.42 (1.00-2.03) | 1.51 (1.12-2.03) | - | - | - | - | - | - |
| Parental antisocial behaviour | - | - | - | - | - | - | 1.14 (0.74-1.76) | 1.31 (0.96-1.79) | 1.96 (1.35-2.85) |
| *Reference group: neither self-harm or depression (ALSPAC n= 5,810^^^ ; MCS n= 8,600 ; E-Risk n= 1,515). ^^^From m=1. ^+^And/or emotional neglect in the E-Risk study  RRR = relative risk ratio; 95% CI = 95% Confidence Interval.  The Avon Longitudinal Study of Parents and Children (ALSPAC); The Millennium Cohort Study (MCS); The Environmental Risk (E-Risk) Longitudinal Twin Study. | | | | | | | | | |

Appendix S6: Interactions by sex

There were few differences by sex. In the ALSPAC cohort, the association between physical and emotional abuse and co-occurring self-harm and depression was stronger for females (physical abuse: aRRR 2.54, 1.79-3.60; emotional abuse: aRRR 2.70, 1.98-3.67) than males (physical abuse: aRRR 0.96, 0.68-1.36; emotional abuse: aRRR 1.42, 1.07-1.88). In the MCS, the association between bullying and self-harm alone was stronger for males (aRRR 2.14, 1.54-2.97) than females (aRRR 1.38, 1.08-1.76). In the E-Risk study, physical neglect was associated with an increased risk of self-harm alone among males (aRRR 3.75, 1.73-8.15), but there was little evidence of an association among females (aRRR 0.82, 0.33-2.02). Results are stratified by sex in Tables S12a and S12b below.

Table S12a: Stratified by sex, adjusted relative risk ratios for the association between adverse childhood experiences and self-harm and depression among males.

|  | **ALSPAC** | | | **MCS** | | | **E-Risk** | | |
| --- | --- | --- | --- | --- | --- | --- | --- | --- | --- |
| Adverse childhood experience | Self-harm alone*  (n= 993) | Depression alone*  (n= 973) | Co-occurring self-harm and depression*  (n= 1,735) | Self-harm alone (n= 714) | Depression alone (n= 801) | Co-occurring self-harm and depression (n= 893) | Self-harm alone (n=132) | Depression alone (n=253) | Co-occurring self-harm and depression (n=161) |
|  | RRR (95% CI) | RRR (95% CI) | RRR (95% CI) | RRR (95% CI) | RRR (95% CI) | RRR (95% CI) | RRR (95% CI) | RRR (95% CI) | RRR (95% CI) |
| Parental mental health problems | 1.06 (0.76-1.49) | 1.82 (1.28-2.59) | 1.36 (1.08-1.70) | 1.45 (1.04-2.01) | 1.35 (0.99-1.85) | 1.57 (1.05-2.35) | 1.58 (0.74-3.39) | 0.92 (0.59-1.44) | 1.68 (0.83-3.39) |
| Domestic violence | 1.19 (0.80-1.77) | 1.44 (0.91-2.27) | 1.44 (1.05-1.96) | 1.44 (0.99-2.09) | 1.07 (0.73-1.56) | 1.41 (0.91-2.17) | 1.85 (0.91-3.78) | 1.17 (0.73-1.88) | 2.54 (1.37-4.70) |
| Substance abuse | 1.09 (0.63-1.90) | 2.12 (1.30-3.45) | 1.70 (1.19-2.43) | 1.21 (0.89-1.63) | 1.10 (0.84-1.46) | 1.42 (0.94-2.16) | 1.20 (0.50-2.90) | 0.98 (0.50-1.91) | 2.35 (1.15-4.80) |
| Bullying | 1.07 (0.67-1.71) | 1.86 (1.22-2.85) | 1.51 (1.11-2.04) | 2.14 (1.54-2.97) | 1.55 (1.12-2.16) | 1.94 (1.33-2.84) | 1.81 (1.01-3.26) | 1.69 (1.11-2.57) | 3.10 (1.60-6.01) |
| Physical abuse | 1.15 (0.75-1.76) | 1.14 (0.74-1.77) | 0.96 (0.68-1.36) | 1.13 (0.80-1.59) | 1.27 (0.92-1.75) | 1.29 (0.86-1.93) | 1.47 (0.72-2.99) | 1.21 (0.71-2.05) | 1.13 (0.61-2.10) |
| Physical neglect | - | - | - | - | - | - | 3.75 (1.73-8.15) | 1.00 (0.47-2.11) | 1.46 (0.59-3.59) |
| Sexual abuse^a^ | 1.54 (0.65-3.66) | 1.59 (0.58-4.40) | 1.26 (0.55-2.88) | - | - | - | - | - | 9.54 (2.49-36-55) |
| Emotional abuse^+^ | 1.13 (0.74-1.71) | 1.98 (1.33-2.96) | 1.42 (1.07-1.88) | - | - | - | 1.68 (0.74-3.78) | 1.38 (0.74-2.55) | 1.31 (0.60-2.84) |
| Emotional neglect | 0.77 (0.51-1.17) | 1.15 (0.72-1.82) | 0.93 (0.66-1.32) | - | - | - | - | - | - |
| Separation/divorce | 1.02 (0.70-1.50) | 1.72 (1.12-2.64) | 1.41 (1.03-1.93) | 1.18 (0.83-1.66) | 1.58 (1.19-2.11) | 1.61 (1.05-2.46) | 1.07 (0.54-2.11) | 1.14 (0.71-1.84) | 1.39 (0.74-2.59) |
| Parental conviction | 1.10 (0.62-1.95) | 0.95 (0.46-1.96) | 1.08 (0.71-1.65) | - | - | - | - | - | - |
| Parental antisocial behaviour | - | - | - | - | - | - | 1.56 (0.75-3.27) | 1.42 (0.85-2.38) | 1.95 (1.03-3.72) |
| The Avon Longitudinal Study of Parents and Children (ALSPAC): Adjusted for household social class, parity, home ownership status, mother’s educational qualifications, mother’s age at delivery.  The Millennium Cohort Study (MCS): Adjusted for child and mother’s ethnicity, mother’s age at delivery, mother’s education, household income, family housing tenure.  The Environmental Risk (E-Risk) Longitudinal Twin Study: Adjusted for household social class, mother’s age at delivery, mother’s qualifications. | | | | | | | | | |
| *Reference group: neither self-harm or depression (ALSPAC n= 5,810^^^; MCS n= 8,600; E-Risk n= 1,515). ^^^From m=1. ^+^And/or emotional neglect in the E-Risk study  ^a^No observations in E-Risk Study among males for the outcomes self-harm alone and depression alone.  RRR = relative risk ratio; 95% CI = 95% Confidence Interval. | | | | | | | | | |

Table S12b: Stratified by sex, adjusted relative risk ratios for the association between adverse childhood experiences and self-harm and depression among females

|  | **ALSPAC** | | | **MCS** | | | **E-Risk** | | |
| --- | --- | --- | --- | --- | --- | --- | --- | --- | --- |
| Adverse childhood experience | Self-harm alone*  (n= 993) | Depression alone*  (n= 973) | Co-occurring self-harm and depression*  (n= 1,735) | Self-harm alone (n= 714) | Depression alone (n= 801) | Co-occurring self-harm and depression (n= 893) | Self-harm alone (n=132) | Depression alone (n=253) | Co-occurring self-harm and depression (n=161) |
|  | RRR (95% CI) | RRR (95% CI) | RRR (95% CI) | RRR (95% CI) | RRR (95% CI) | RRR (95% CI) | RRR (95% CI) | RRR (95% CI) | RRR (95% CI) |
| Parental mental health problems | 1.31 (0.99-1.73) | 1.43 (1.13-1.81) | 1.64 (1.25-2.16) | 1.17 (0.93-1.48) | 1.45 (1.15-1.84) | 1.55 (1.25-1.92) | 1.32 (0.78-2.23) | 1.84 (1.23-2.75) | 2.35 (1.45-3.79) |
| Domestic violence | 1.40 (0.97-2.01) | 1.10 (0.80-1.51) | 1.57 (1.07-2.31) | 1.34 (1.04-1.72) | 1.32 (1.00-1.73) | 1.41 (1.12-1.77) | 1.22 (0.76-1.98) | 1.28 (0.85-1.92) | 1.52 (0.95-2.43) |
| Substance abuse | 1.33 (0.87-2.05) | 1.49 (1.01-2.18) | 1.81 (1.22-2.69) | 1.24 (0.98-1.58) | 1.01 (0.78-1.30) | 1.14 (0.92-1.41) | 0.90 (0.45-1.78) | 1.29 (0.77-2.18) | 2.32 (1.30-4.14) |
| Bullying | 1.19 (0.81-1.74) | 1.67 (1.26-2.21) | 1.63 (1.12-2.35) | 1.38 (1.08-1.76) | 1.38 (1.09-1.75) | 1.66 (1.38-2.00) | 1.19 (0.74-1.92) | 1.22 (0.84-1.77) | 2.14 (1.39-3.29) |
| Physical abuse | 1.42 (0.99-2.04) | 1.71 (1.24-2.35) | 2.54 (1.79-3.60) | 1.07 (0.79-1.45) | 0.93 (0.69-1.25) | 1.19 (0.94-1.50) | 2.22 (1.23-4.01) | 1.87 (1.16-3.01) | 2.29 (1.33-3.94) |
| Physical neglect | - | - | - | - | - | - | 0.82 (0.33-2.02) | 1.05 (0.56-1.95) | 1.91 (0.85-4.27) |
| Sexual abuse | 2.07 (1.18-3.61) | 1.59 (0.92-2.73) | 2.26 (1.31-3.90) | - | - | - | 1.34 (0.32-5.58) | 3.91 (1.52-10.05) | 2.24 (0.66-7.59) |
| Emotional abuse^+^ | 1.32 (0.94-1.85) | 1.94 (1.49-2.52) | 2.70 (1.98-3.67) | - | - | - | 2.01 (1.05-3.85) | 1.66 (0.94-2.91) | 2.22 (1.06-4.63) |
| Emotional neglect | 1.30 (0.91-1.85) | 1.08 (0.77-1.53) | 1.34 (0.87-2.04) | - | - | - | - | - | - |
| Separation/divorce | 1.35 (1.00-1.83) | 1.27 (0.97-1.65) | 1.66 (1.24-2.24) | 1.22 (0.96-1.55) | 1.34 (1.04-1.72) | 1.18 (0.96-1.44) | 1.14 (0.65-2.01) | 1.04 (0.69-1.55) | 1.16 (0.72-1.88) |
| Parental conviction | 1.20 (0.74-1.94) | 1.56 (1.07-2.27) | 1.89 (1.21-2.96) | - | - | - | - | - | - |
| Parental antisocial behaviour | - | - | - | - | - | - | 0.57 (0.32-1.04) | 0.97 (0.61-1.55) | 1.54 (0.93-2.54) |
| The Avon Longitudinal Study of Parents and Children (ALSPAC): Adjusted for household social class, parity, home ownership status, mother’s educational qualifications, mother’s age at delivery.  The Millennium Cohort Study (MCS): Adjusted for child and mother’s ethnicity, mother’s age at delivery, mother’s education, household income, family housing tenure.  The Environmental Risk (E-Risk) Longitudinal Twin Study: Adjusted for household social class, mother’s age at delivery, mother’s qualifications. | | | | | | | | | |
| *Reference group: neither self-harm or depression (ALSPAC n= 5,810^^^; MCS n= 8,600; E-Risk n= 1,515). ^^^From m=1. ^+^And/or emotional neglect in the E-Risk study  RRR = relative risk ratio; 95% CI = 95% Confidence Interval. | | | | | | | | | |

Figure S2a: Elbow plots showing variation against number of variables selected during the Least Absolute Shrinkage and Selection Operator (lasso) procedure, for the association between each adverse childhood experience (in early childhood, middle childhood, and early adolescence) and self-harm, depression, and co-occurring self-harm and depression (at age 16 years) in ALSPAC.


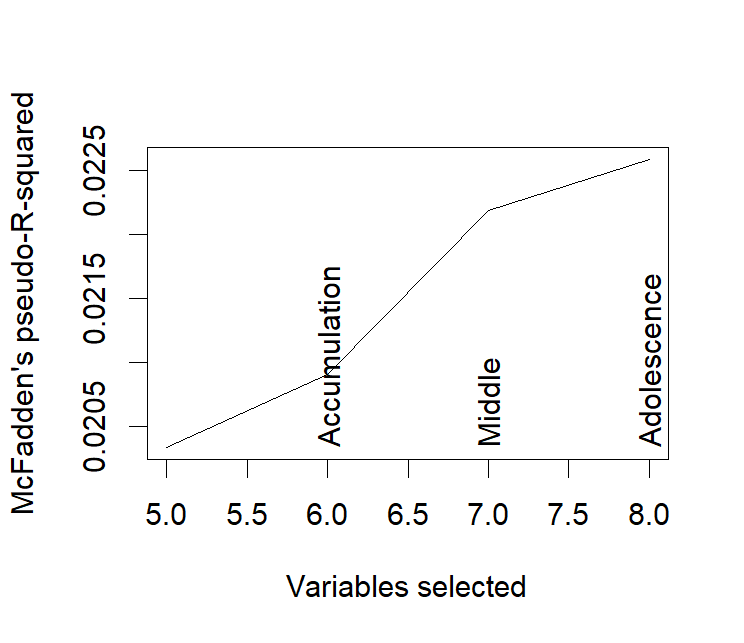

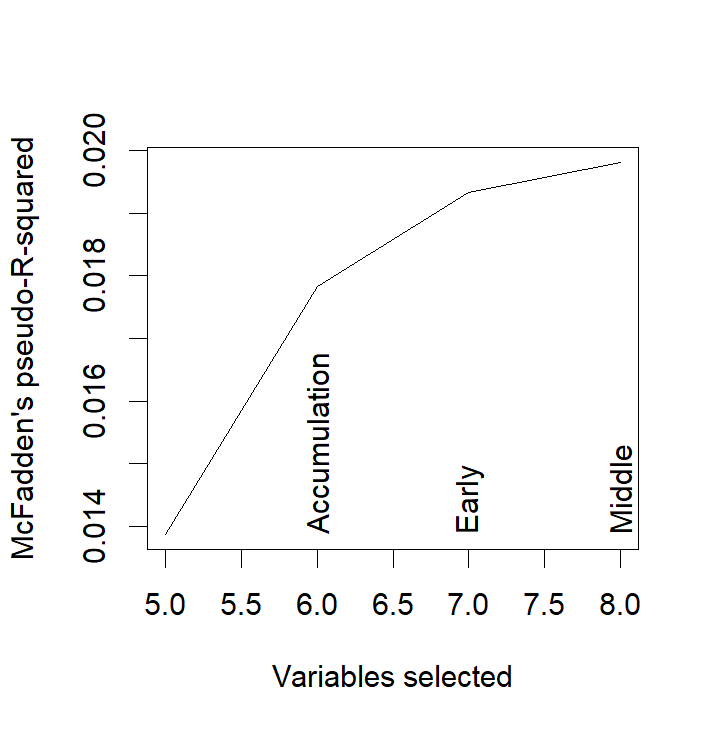

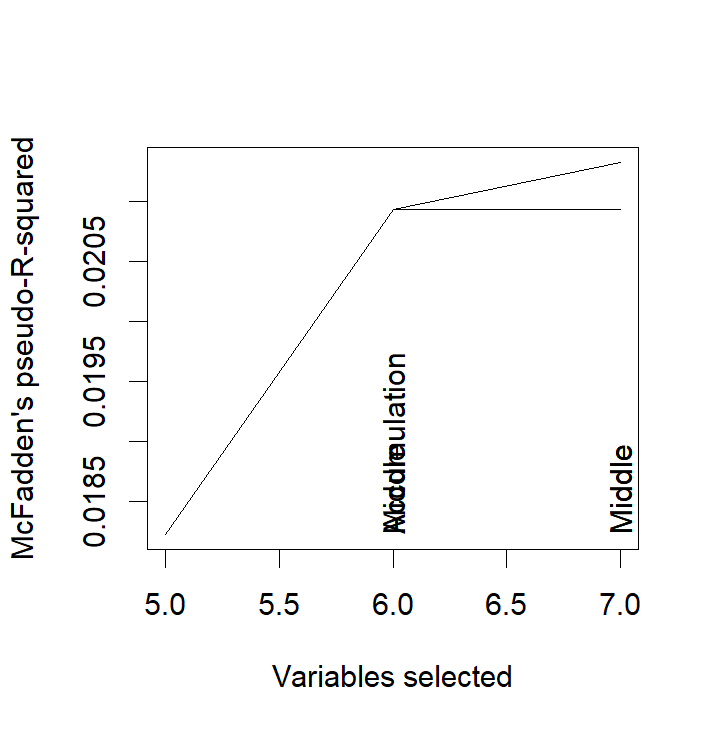


**Figure 1A: Parental mental health problems**

Self-harm Depression Co-occurring self-harm and depression


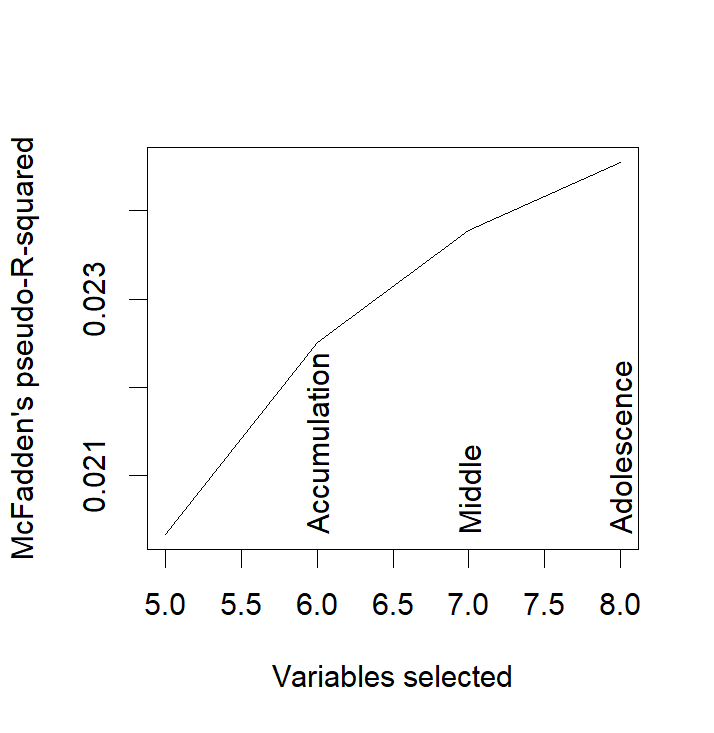

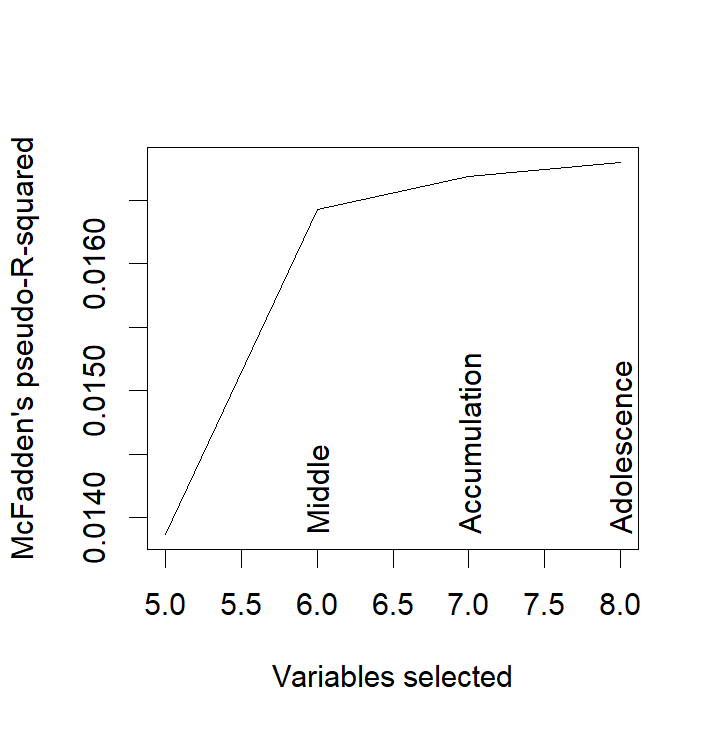

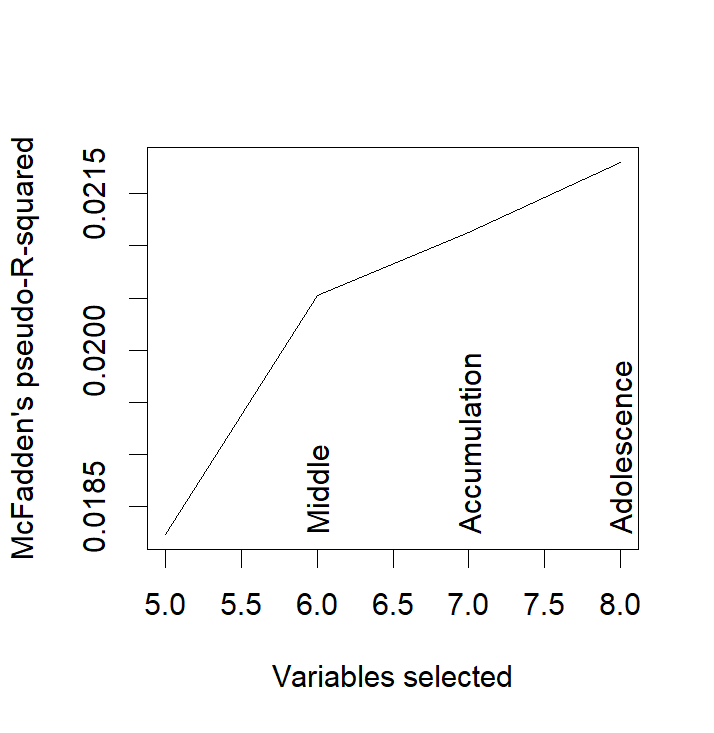


**Figure 1B: Domestic violence**

Self-harm Depression Co-occurring self-harm and depression

**Figure 1C: Physical abuse**


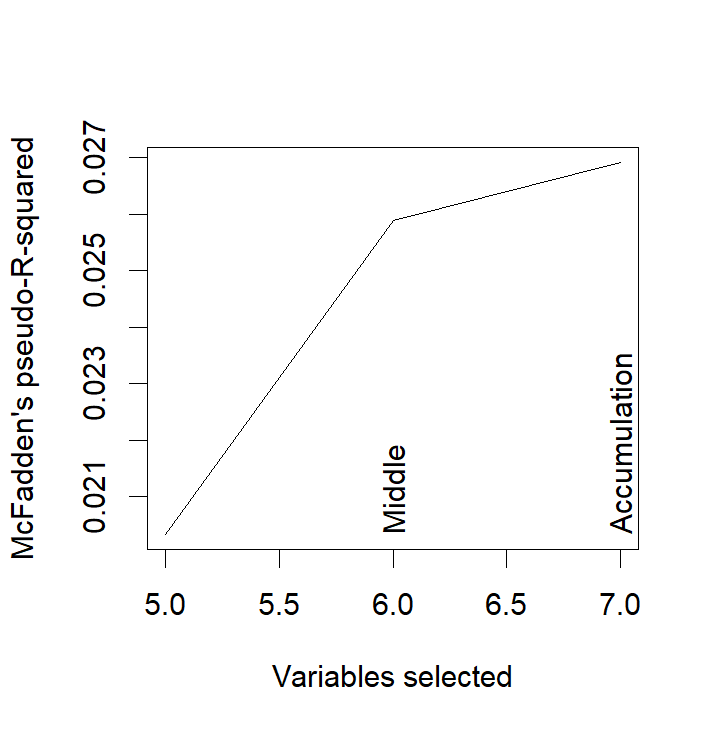

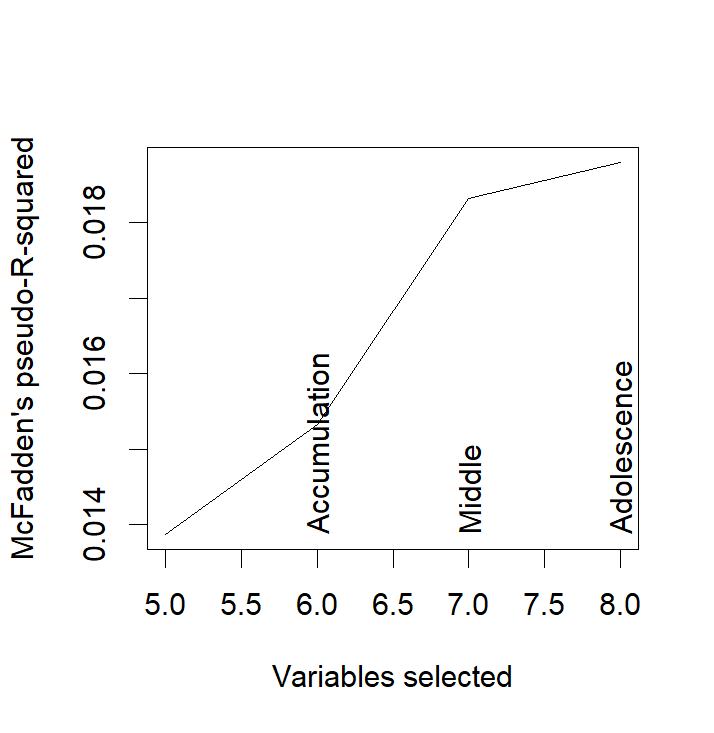

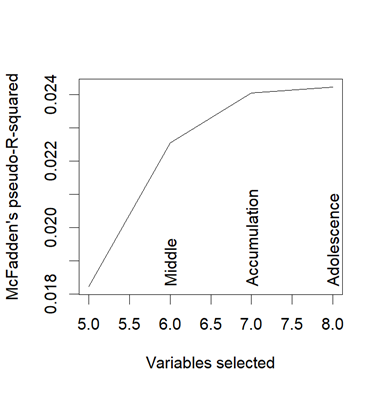


Self-harm Depression Co-occurring self-harm and depression

**Figure 1D: Emotional abuse**

Self-harm Depression Co-occurring self-harm and depression


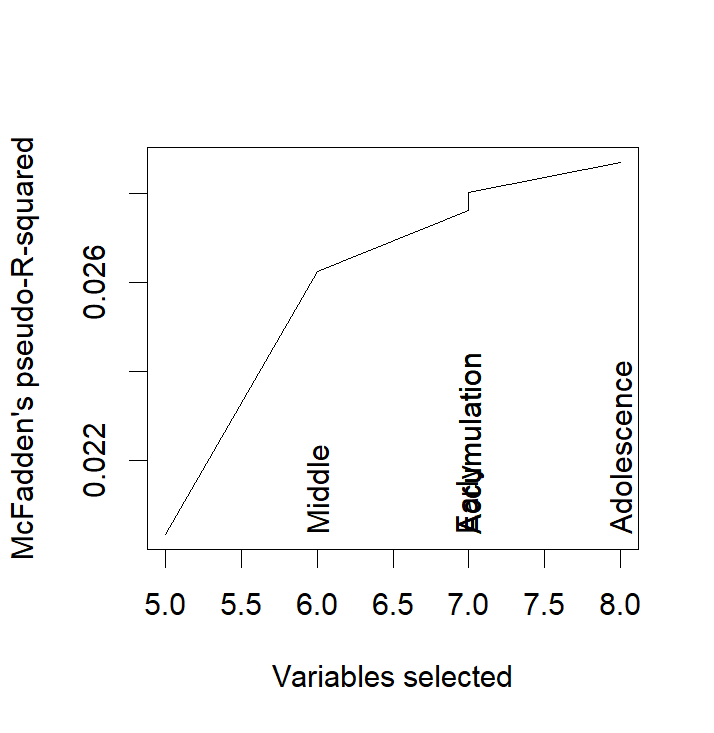

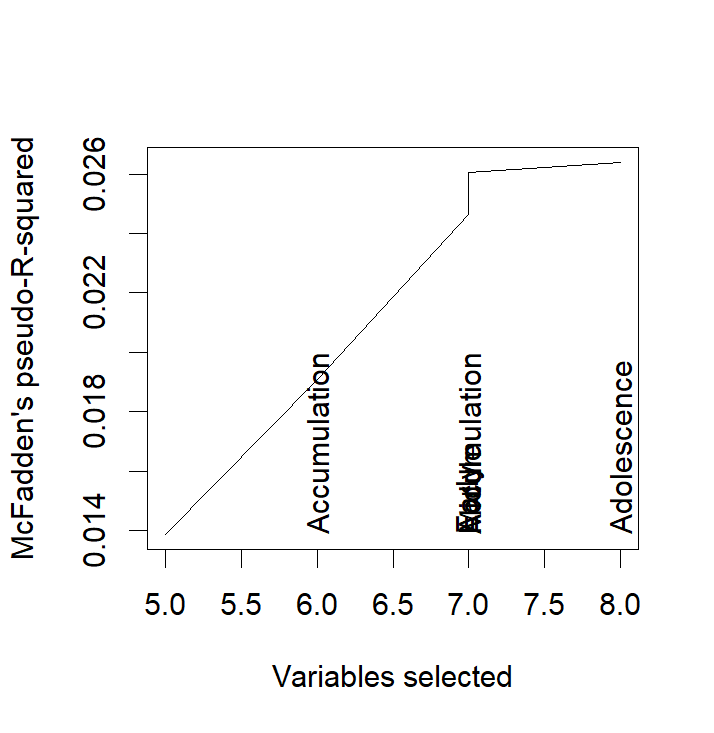

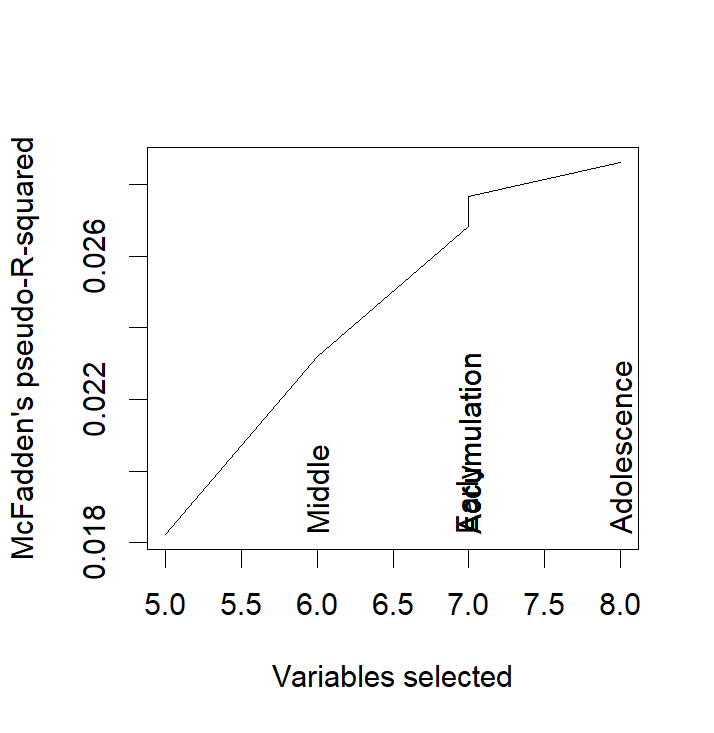


**Figure 1E: Separation/divorce**


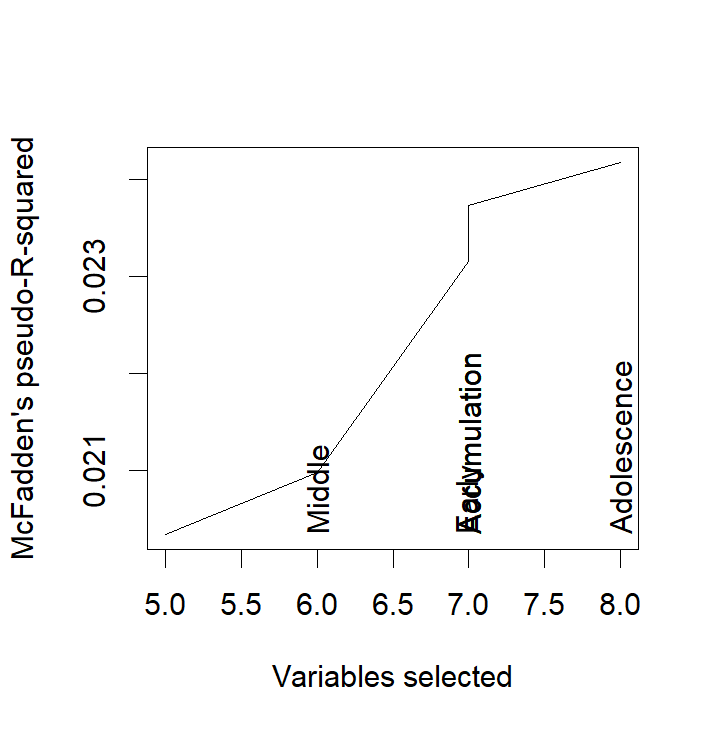

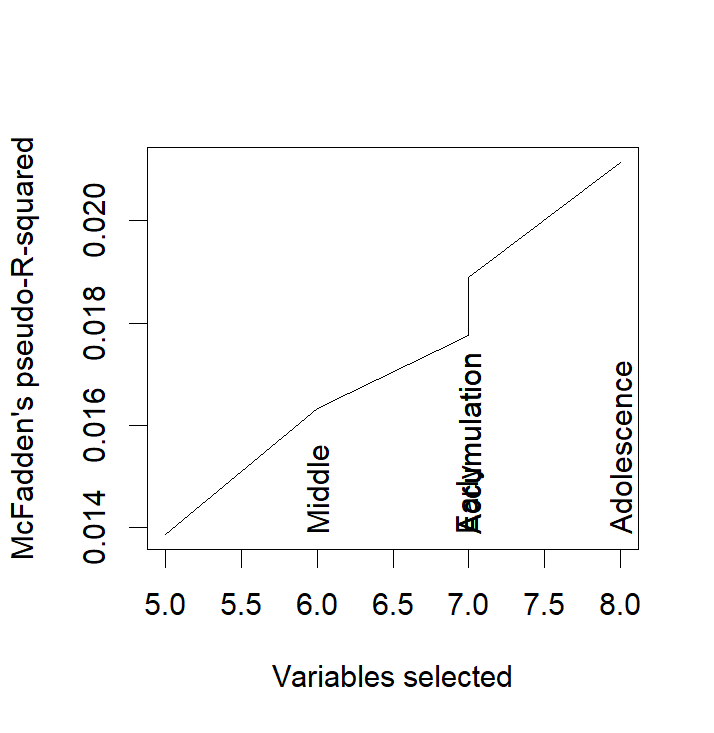

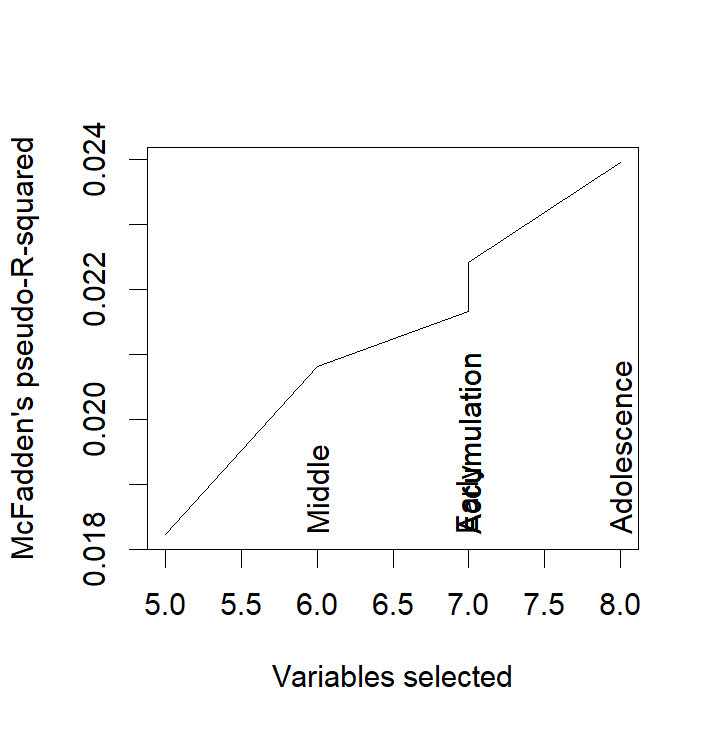


Self-harm Depression Co-occurring self-harm and depression

**Figure 1F: Parental conviction**


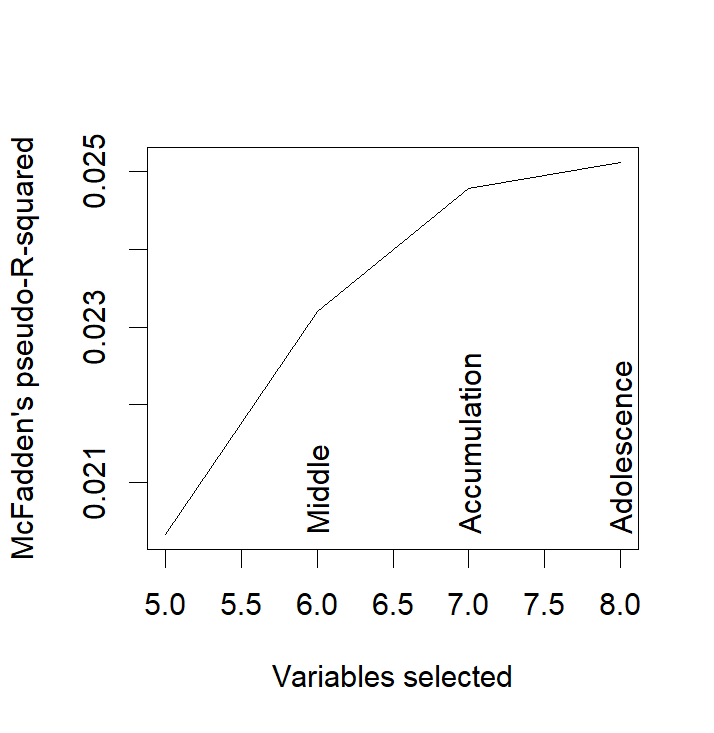

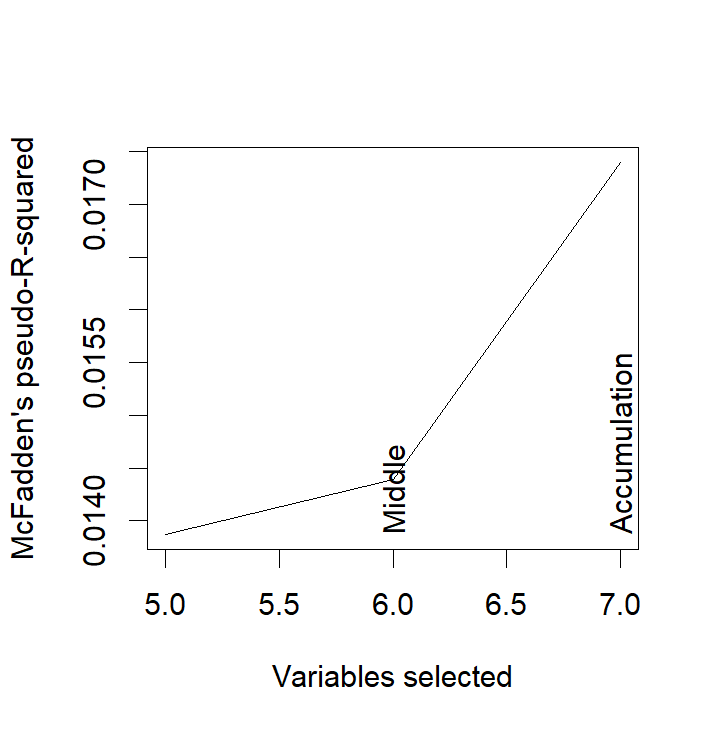

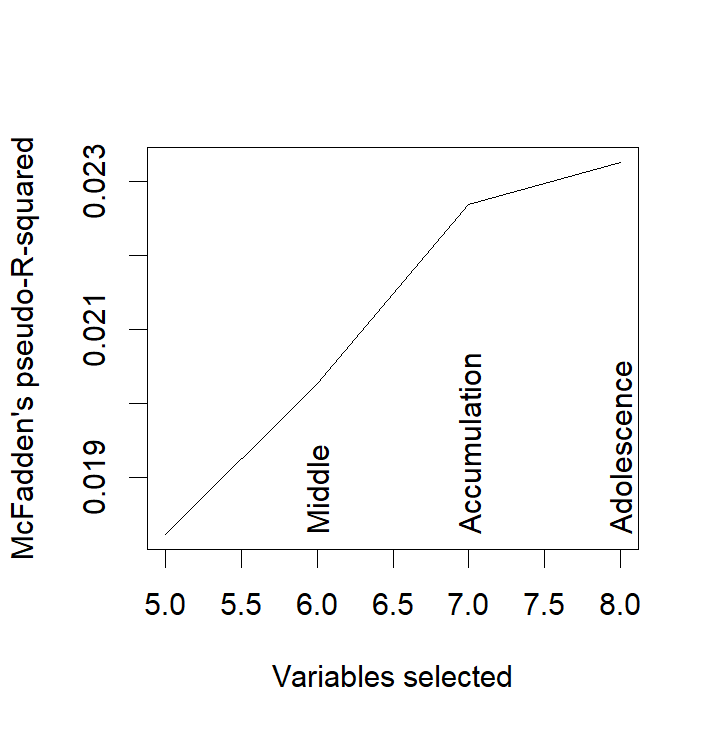


Self-harm Depression Co-occurring self-harm and depression

Figure S2b: Elbow plots showing variation against number of variables selected during the Least Absolute Shrinkage and Selection Operator (lasso) procedure, for the association between each adverse childhood experience (in early childhood, middle childhood, and early adolescence) and self-harm, depression, and co-occurring self-harm and depression (at age 14 years) in MCS.

**Figure 2A: Parental mental health problems**


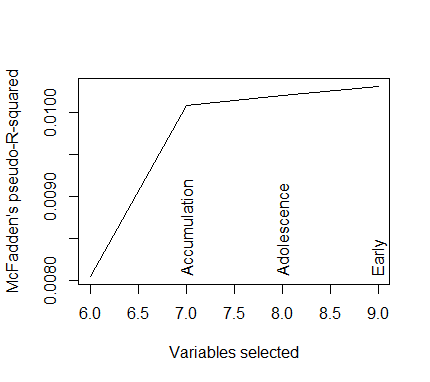

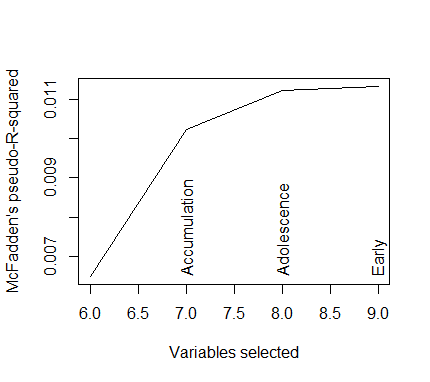

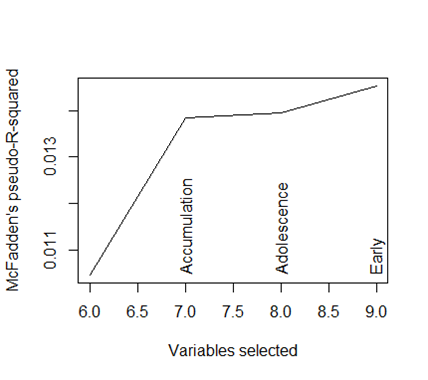


Self-harm Depression Co-occurring self-harm and depression

**Figure 2B: Domestic violence**


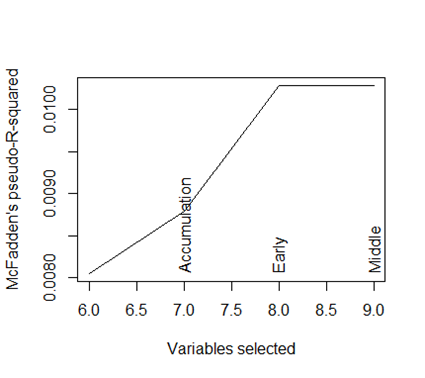

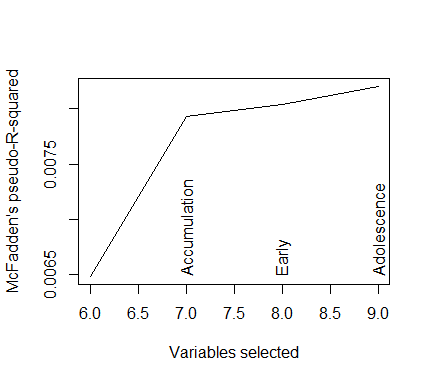

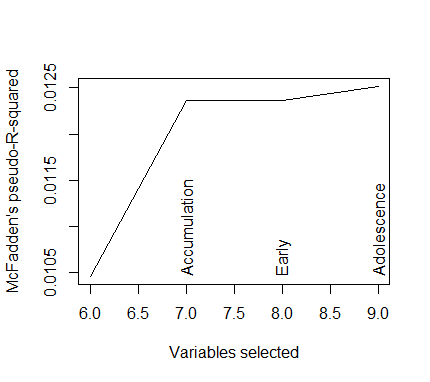


Self-harm Depression Co-occurring self-harm and depression

**Figure 2C: Bullying**


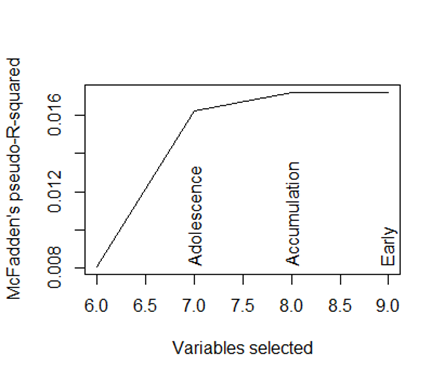

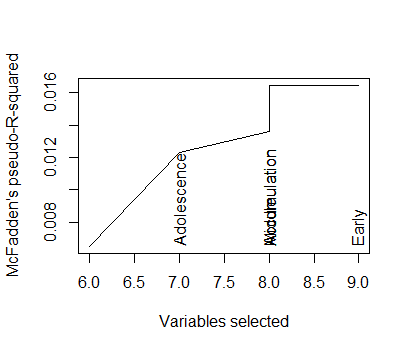

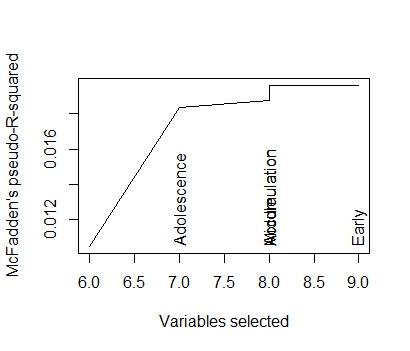


Self-harm Depression Co-occurring self-harm and depression

**Figure 2D: Substance abuse**


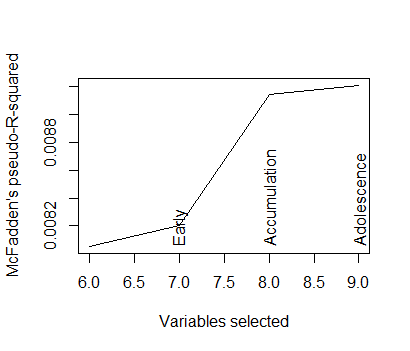

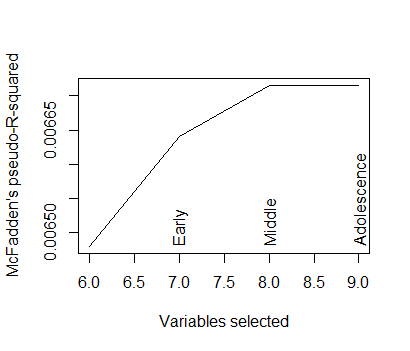

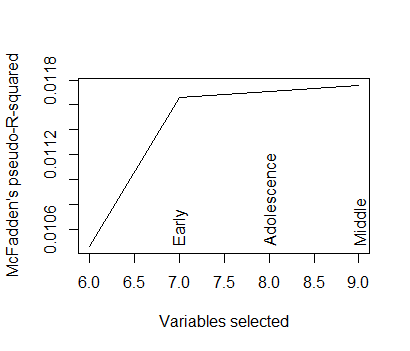


Self-harm Depression Co-occurring self-harm and depression

**Figure 2E: Separation/divorce**


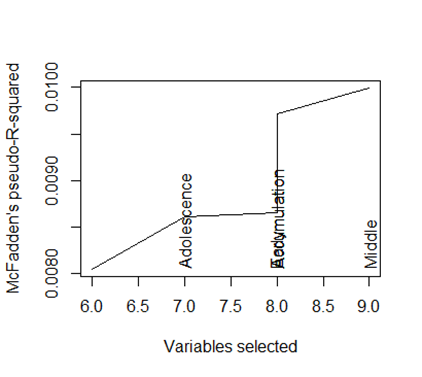

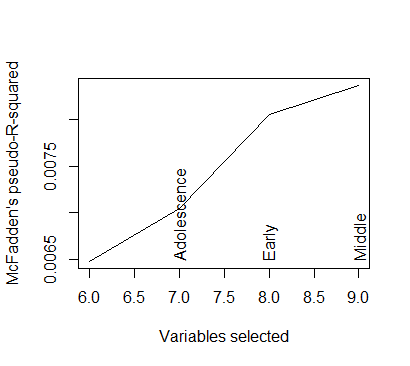

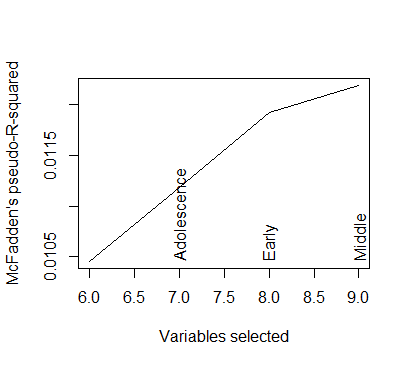


Self-harm Depression Co-occurring self-harm and depression

Early refers to exposure to an ACE during the early child period, between birth and 5 years of age; Middle refers to exposure to an ACE during the middle childhood period, between 6 and 10 years of age; Adolescence refers to exposure to an ACE during early adolescence, between 11 and 13 years of age; Accumulation refers to cumulative exposure to the same ACE across early childhood, middle childhood, and adolescence (i.e. sum of the three critical periods).

The elbow plots show the outcome variation explained (pseudo R^2^) by each model after adding the life course hypotheses (early childhood, middle childhood, adolescence, accumulation of risk). Each hypothesis on the elbow plots above is presented in order of the strength of its’ association with the outcome variation. In our paper we present the first hypotheses selected by the lasso. This corresponds to the hypothesis with the strongest association with the outcome, explaining the greatest proportion of variance in the outcome. Additional hypotheses are selected by the lasso in order of strength of association with outcome variation. For example, for the ACE separation/divorce (Figure 2E) in the MCS, the adolescence period was the first variable selected by lasso as it was the simplest hypothesis explaining the greatest variation in depression. The next two hypotheses selected by the lasso were early childhood, and middle childhood. The first peak in the elbow plots indication the most optimum number of variables (represented by the ACE occurring at each of the three critical periods or accumulation of the ACE) after adjusting for confounders.

Figure S2c Tetrachoric correlations for each ACE over time in ALSPAC and MCS.


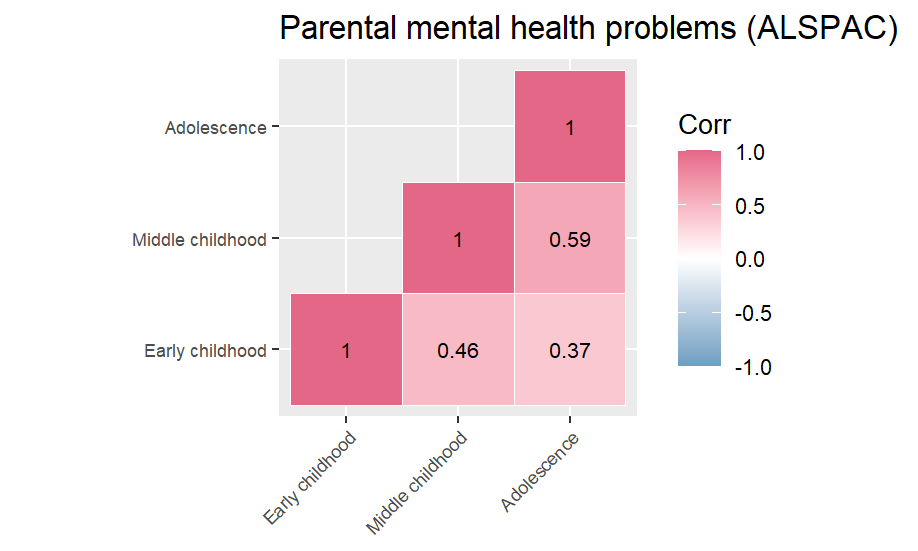

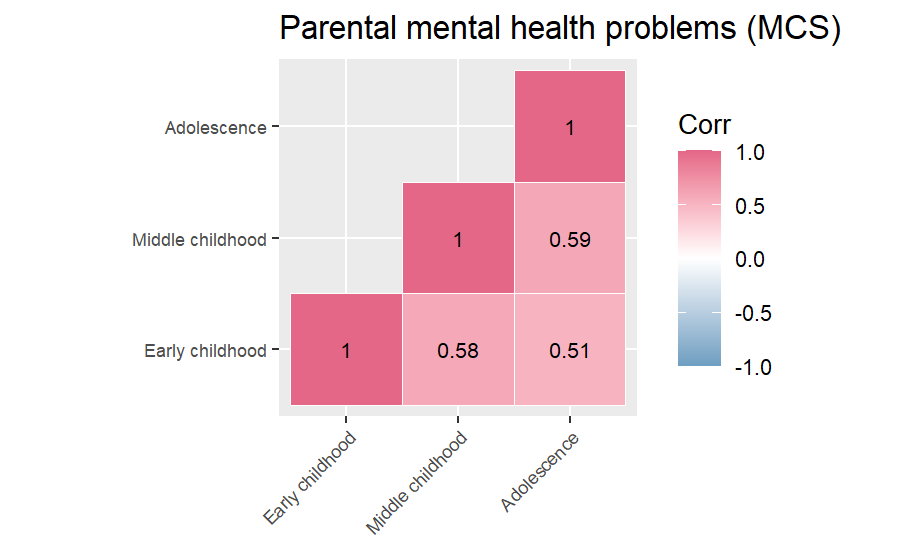

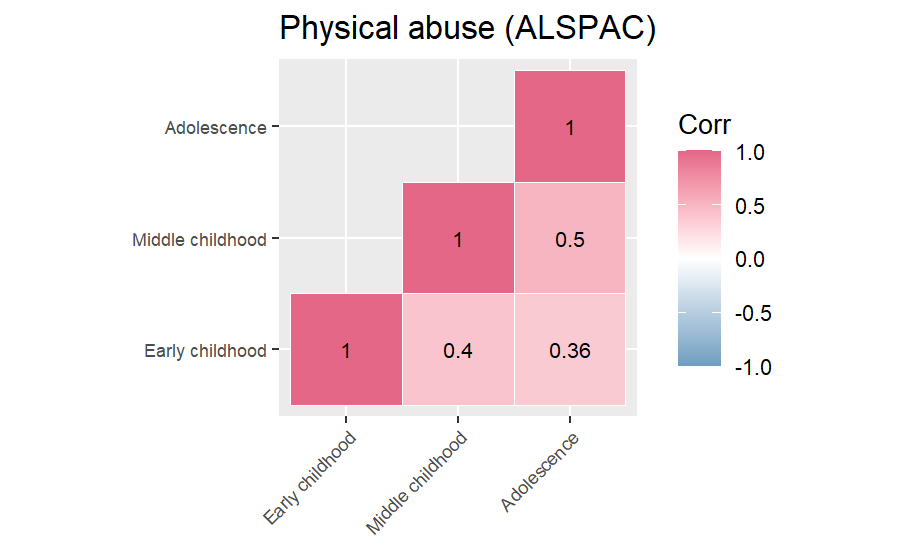

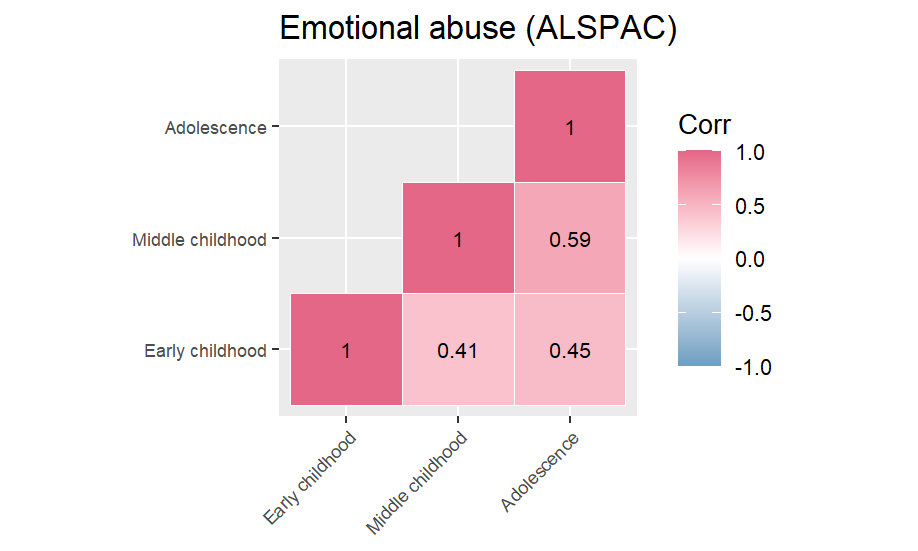

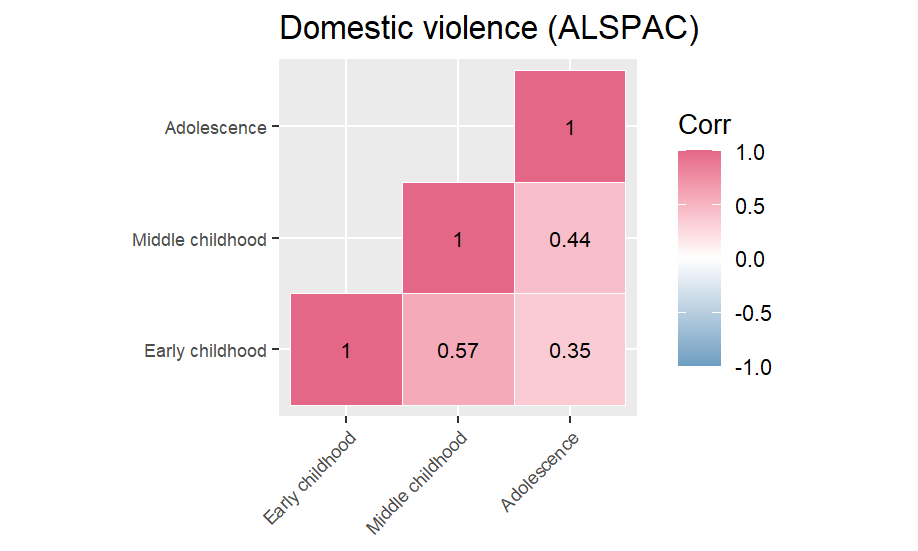

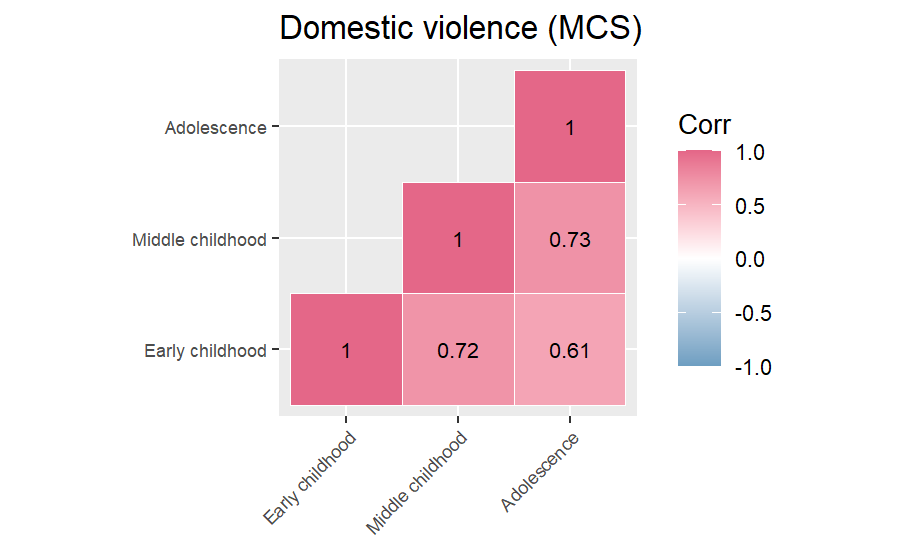

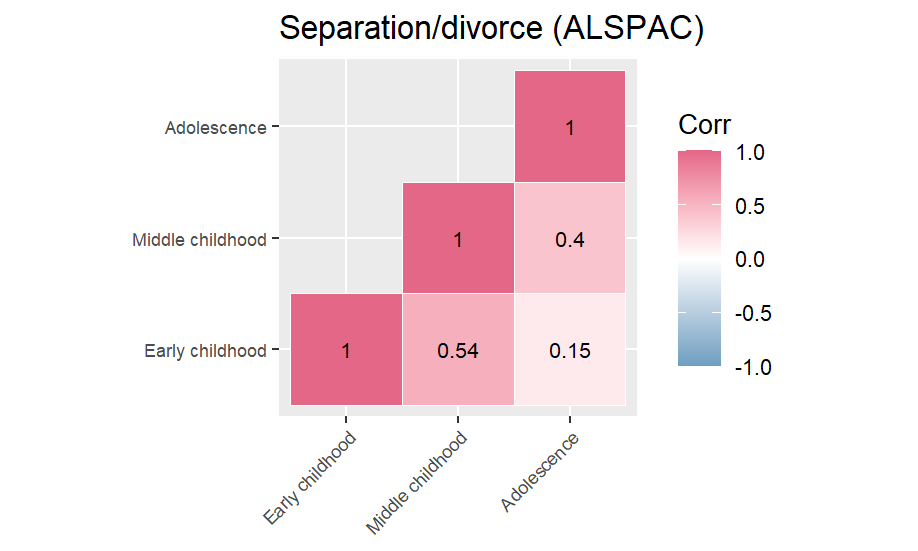

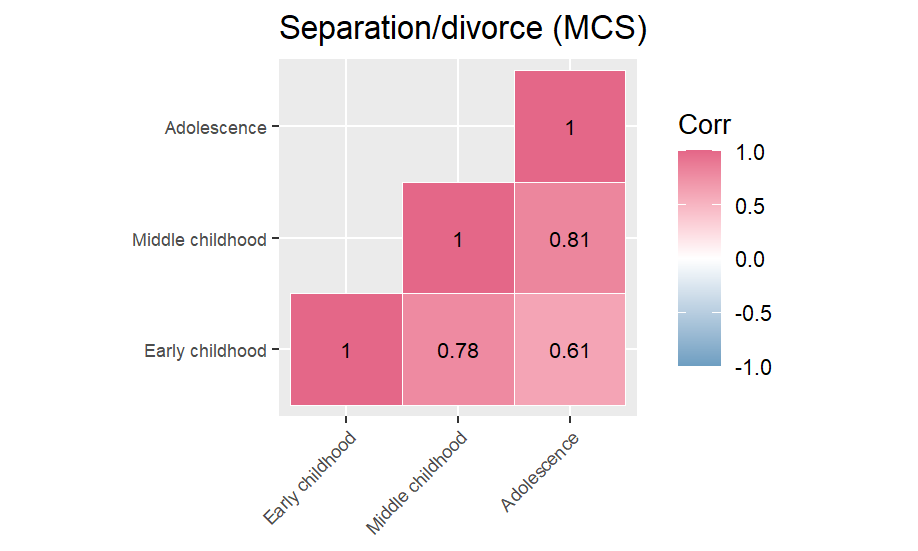

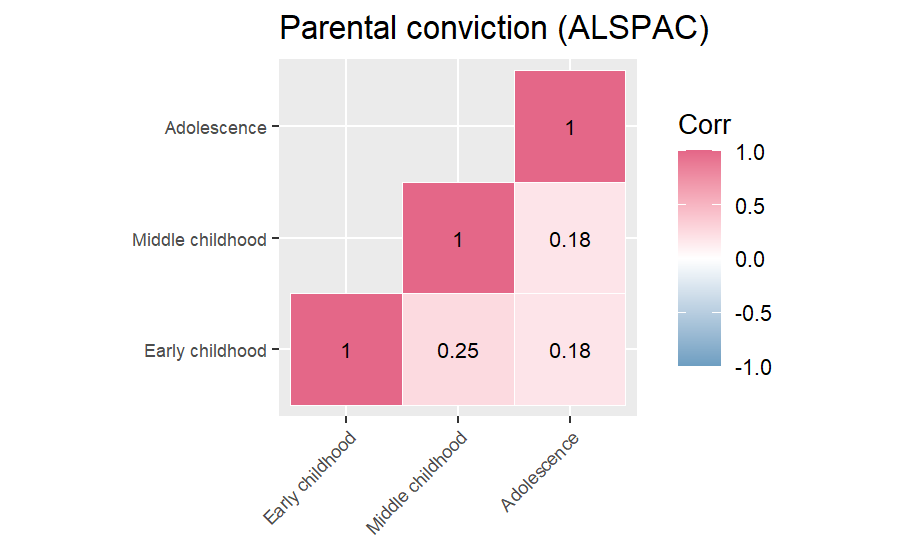

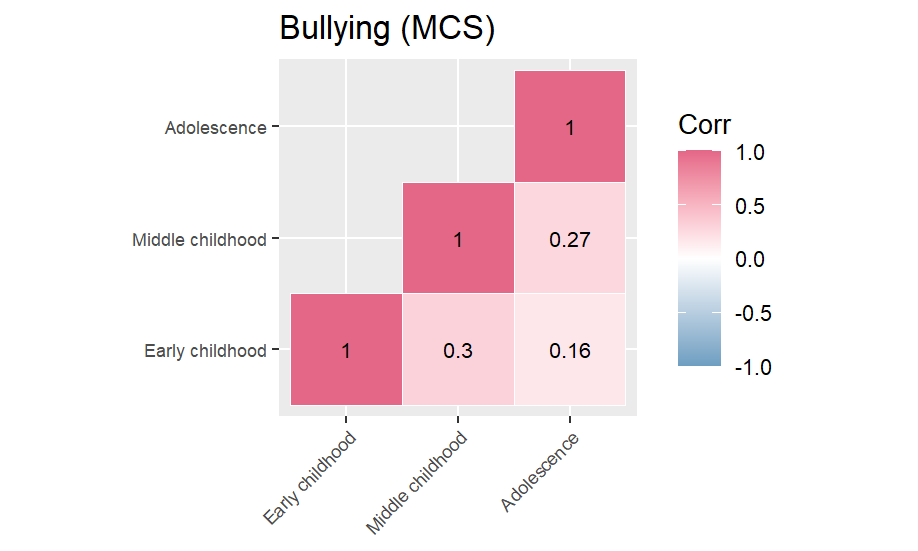

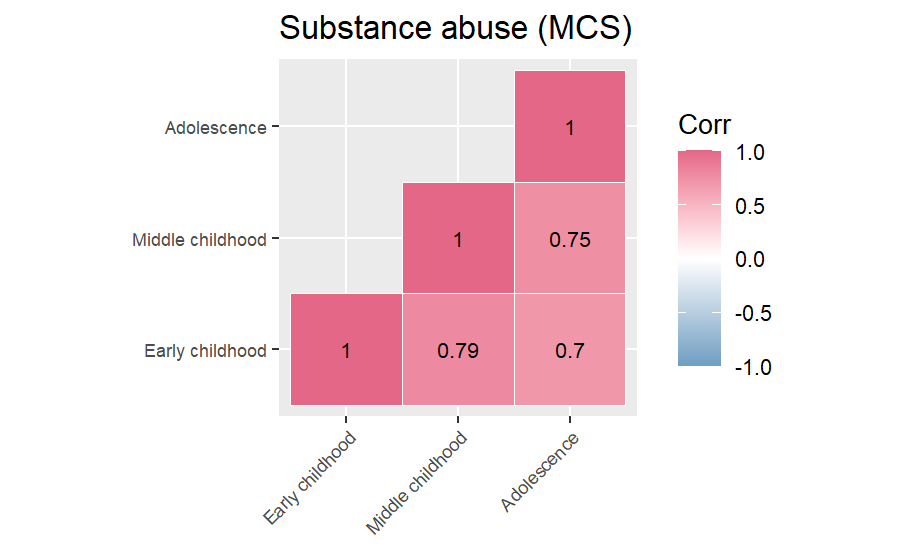


Appendix S7: ALSPAC complete-case sensitivity analyses

Table S13a shows results of analyses among those with complete data on exposures, outcomes, and confounders in the ALSPAC study for the association between individual adverse childhood experiences and self-harm and depression.

*Self-harm alone*

Consistent with findings from the multiply imputed data, there was weak evidence of an association between domestic violence and self-harm, however no association for physical abuse and self-harm were found among the complete-cases. Results for sexual abuse were consistent with the multiply imputed data, although the effect estimate was larger. Additionally, there were associations in the complete-cases that were not found in the multiply imputed data; parental mental health problems (aRRR 1.65, 95% CI 1.15-2.35), and emotional abuse (aRRR 1.57, 95% CI 1.01-2.43) were associated with an increased risk of self-harm.

*Depression alone*

Findings were consistent with the multiply imputed data for the association between parental mental health problems, bullying, physical abuse, sexual abuse, emotional abuse and depression. However, in the complete-cases no association were found for substance abuse and separation/divorce and depression.

*Co-occurring self-harm and depression*

Results for domestic violence, substance abuse, bullying, physical abuse, emotional abuse, and parental mental health problems and co-occurring self-harm and depression were consistent with the multiply imputed data, although the evidence of an association between parental mental health problems and co-occurring self-harm and depression was weak among the complete-cases. However, no associations for separation/divorce and parental conviction were found among the complete-cases. In contrast to the multiply imputed data, there was an increased risk of co-occurring self-harm and depression among those exposed to sexual abuse (aRRR 3.06, 95% CI 1.23-7.63).

Table S13a: Crude and adjusted relative risk ratios for the association between adverse childhood experiences and self-harm and depression in ALSPAC complete-cases

|  | Neither self-harm or depression n= 1,800 | Self-harm alone n= 136 | Depression alone n= 215 | Co-occurring self-harm and depression n= 83 | Neither self-harm or depression n= 1,800 | Self-harm alone n= 136 | Depression alone n= 215 | Co-occurring self-harm and depression n= 83 |
| --- | --- | --- | --- | --- | --- | --- | --- | --- |
|  | Model 1: unadjusted | | | | Model 2: adjusted | | | |
|  | Reference group | RRR (95% CI) | RRR (95% CI) | RRR (95% CI) | Reference group | RRR (95% CI) | RRR (95% CI) | RRR (95% CI) |
| Parental mental health problems | - | 1.66 (1.17-2.35) | 1.73 (1.30-2.30) | 1.53 (0.98-2.38) | - | 1.65 (1.15-2.35) | 1.67 (1.25-2.23) | 1.50 (0.96-2.35) |
| Domestic violence | - | 1.56 (1.02-2.40) | 1.05 (0.71-1.55) | 1.71 (1.01-2.90) | - | 1.51 (0.98-2.35) | 1.06 (0.71-1.58) | 1.72 (1.01-2.94) |
| Substance abuse | - | 1.45 (0.80-2.65) | 1.57 (0.97-2.53) | 2.55 (1.37-4.75) | - | 1.30 (0.70-2.40) | 1.50 (0.92-2.44) | 2.36 (1.25-4.45) |
| Bullying | - | 1.31 (0.81-2.13) | 1.95 (1.37-2.79) | 1.99 (1.16-3.42) | - | 1.35 (0.83-2.22) | 2.02 (1.41-2.90) | 2.08 (1.20-3.60) |
| Physical abuse | - | 1.23 (0.73-2.09) | 1.62 (1.09-2.41) | 3.31 (2.00-5.47) | - | 1.22 (0.71-2.10) | 1.62 (1.08-2.42) | 3.33 (2.00-5.57) |
| Sexual abuse | - | 4.44 (2.20-8.95) | 2.72 (1.36-5.44) | 3.93 (1.60-9.62) | - | 3.35 (1.63-6.89) | 2.15 (1.06-4.36) | 3.06 (1.23-7.63) |
| Emotional abuse | - | 1.61 (1.05-2.48) | 2.00 (1.43-2.79) | 3.20 (2.00-5.11) | - | 1.57 (1.01-2.43) | 1.97 (1.40-2.77) | 3.18 (1.97-5.13) |
| Emotional neglect | - | 1.23 (0.78-1.95) | 0.93 (0.62-1.40) | 1.37 (0.78-2.40) | - | 1.31 (0.82-2.09) | 0.94 (0.62-1.43) | 1.47 (0.83-2.60) |
| Separation/divorce | - | 1.25 (0.79-1.96) | 1.19 (0.82-1.73) | 1.65 (0.97-2.79) | - | 1.17 (0.73-1.88) | 1.12 (0.76-1.64) | 1.58 (0.91-2.74) |
| Parental conviction | - | 0.57 (0.23-1.42) | 1.45 (0.87-2.41) | 1.16 (0.50-2.73) | - | 0.51 (0.20-1.28) | 1.42 (0.85-2.38) | 1.08 (0.46-2.56) |
| Model 2: adjusted for sex, household social class, parity, home ownership status, mother’s educational qualifications, mother’s age at delivery  RRR = relative risk ratio; 95% CI = 95% Confidence Interval. | | | | | | | | |

*Developmental timing and duration of exposure – ALSPAC complete-cases*

Due to the large number of lasso models considered, we restricted our reporting here to the model indicated by the first variable selected by lasso (i.e. the simplest hypothesis explaining the greatest variation in the outcome). Table S13b shows the first hypothesis selected by the Least Absolute Shrinkage and Selection Operator (lasso) that best supported the ALSPAC complete-case data, separately for each outcome measure, and the associated effect estimates. The accumulation of risk, early, and middle childhood periods best supported the observed data.

*Early childhood*

In contrast to the ALSPAC multiply imputed data, evidence of an early childhood period emerged from the complete-case data.

For the outcome self-harm, domestic violence (OR 2.05, 99% CI 0.98-4.30) occurring in early childhood was best supported by the complete-case data, as opposed to cumulative exposure supported by the multiply imputed data. Similarly, separation/divorce in early childhood, as opposed to middle childhood, best supported the complete-case data, however the confidence intervals included the null (OR 1.53, 99% CI 0.74-3.17).

For the outcome depression, parental mental health problems (OR 1.69, 99% CI 1.17-2.44) and physical abuse (OR 2.33, 99% CI 1.25-4.35) occurring in early childhood, as opposed to cumulative exposure supported by the multiply imputed data, were best supported by the complete case data. Similarly, early childhood exposure to domestic violence (OR 1.80, 99% CI 0.88-3.69), or separation/divorce (OR 1.42, 99% CI 0.70-2.87), as opposed to middle childhood, best supported the complete-case data for depression, however the confidence intervals included the null.

For the outcome co-occurring self-harm and depression, early childhood exposure to domestic violence (OR 2.56, 99% CI 0.97-6.73), emotional abuse (OR 2.07, 99% CI 0.99-4.32), and separation/divorce (OR 2.03, 99% CI 0.77-5.35) best supported the complete-case data, although the confidence intervals include the null for separation/divorce. However, middle childhood exposure to these ACEs was supported the multiply imputed data.

*Middle childhood*

For the outcome self-harm, consistent with the multiply imputed data, exposure to physical abuse (OR 3.29, 99% CI 0.81-13.45) or parental conviction (OR 2.17, 99% CI 0.69-6.78) in middle childhood was supported the complete-case data, however the confidence intervals included the null.

Exposure to parental mental health problems during middle childhood, as opposed to cumulative exposure among the multiply imputed data, best supported the complete-case data – although the confidence intervals included the null (OR 1.28, 99% CI 0.79-2.05).

There was no evidence of a middle childhood for the outcome depression. For the outcome co-occurring self-harm and depression, exposure to parental mental health problems (OR 1.42, 99% CI 0.72-2.80), and substance abuse (OR 2.78, 99% CI 0.67-11.58) in middle childhood best supported the complete-case data, however the confidence intervals included the null. In the multiply imputed data the accumulation of exposure to parental mental health problems was the best-fitting hypothesis, and substance abuse was not examined.

*Accumulation of risk*

For self-harm, accumulation of exposure to emotional abuse (OR 1.40, 99% CI 1.01-1.95), as opposed to the middle childhood period found in the multiply imputed data, was the best-fitting hypothesis. Similarly, accumulation of exposure to substance abuse best supported the data, although the confidence intervals spanned the null (OR 1.37, 99% CI 0.76-2.50). Substance abuse was not examined in the multiply imputed data.

For the outcome depression, consistent with the multiply imputed data, the accumulation of exposure to emotional abuse best supported the data (OR 1.71, 99% CI 1.28-2.29). Additionally, accumulation of exposure to substance abuse (OR 1.69, 99% CI 0.99-2.88), and parental conviction (OR 1.42, 99% CI 0.81-2.50) supported the complete-case data, although the confidence intervals spanned the null for the latter. Exposure to parental conviction in middle childhood emerged as the best-fitting in the multiply imputed data.

For co-occurring self-harm and depression, accumulation of exposure of physical abuse (OR 1.88, 99% CI 0.97-3.61), and parental conviction (OR 1.48, 99% CI 0.64-3.45) was best supported by the data. However, middle childhood exposure to these ACEs best supported the multiply imputed data.

Table S13b: Adjusted odds ratios and 99% confidence intervals, for the association between the first life course hypothesis selected by the Least Absolute Shrinkage and Selection Operator (lasso) and each outcome measure among the ALSPAC complete-cases.

|  | Self-harm | | | Depression | | | Co-occurring self-harm and depression | | |
| --- | --- | --- | --- | --- | --- | --- | --- | --- | --- |
| Adverse childhood experience | Hypothesis | OR (99% CI) | P-value* | Hypothesis | OR (99% CI) | P-value* | Hypothesis | OR (99% CI) | P-value* |
| Parental mental health problems | Middle childhood | 1.28 (0.79-2.05) | 0.755 | Early childhood | 1.69 (1.17-2.44) | 0.001 | Middle childhood | 1.42 (0.72-2.80) | 0.718 |
| Domestic violence | Early childhood | 2.05 (0.98-4.30) | 0.048 | Early childhood | 1.80 (0.88-3.69) | 0.134 | Early childhood | 2.56 (0.97-6.73) | 0.050 |
| Substance abuse | Accumulation | 1.37 (0.76-2.50) | 0.685 | Accumulation | 1.69 (0.99-2.88) | 0.048 | Middle childhood | 2.78 (0.67-11.58) | 0.262 |
| Physical abuse | Middle childhood | 3.29 (0.81-13.45) | 0.117 | Early childhood | 2.33 (1.25-4.35) | 0.002 | Accumulation | 1.88 (0.97-3.61) | 0.055 |
| Emotional abuse | Accumulation | 1.40 (1.01-1.95) | 0.029 | Accumulation | 1.71 (1.28-2.29) | <0.001 | Early childhood | 2.07 (0.99-4.32) | 0.046 |
| Separation/divorce | Early childhood | 1.53 (0.74-3.17) | 0.532 | Early childhood | 1.42 (0.70-2.87) | 0.811 | Early childhood | 2.03 (0.77-5.35) | 0.242 |
| Parental conviction | Middle childhood | 2.17 (0.69-6.78) | 0.323 | Accumulation | 1.42 (0.81-2.50) | 0.436 | Accumulation | 1.48 (0.64-3.45) | 0.913 |
| *Bonferroni correction applied  OR = Odds Ratio; 99% CI = 99% Confidence Interval. | | | | | | | | | |

Appendix S8: The association between retrospectively reported adverse childhood experiences and self-harm and depression in the E-Risk study.

Retrospective measures of the following adverse childhood experiences were available in the E-Risk study: physical abuse and neglect, emotional abuse and neglect, and sexual abuse. Overall, the effects estimates were larger for retrospectively reported abuse and neglect compared to those reported prospectively. Results were consistent with prospectively reported ACEs in E-Risk and ALSPAC for some ACEs, however some differences were also observed.

The association between retrospectively reported physical abuse and all three outcome measures (self-harm: aRRR 5.30, 95% CI 2.82-9.97; depression: aRRR 2.79, 95% CI 1.55-5.00; both: aRRR 7.03, 95% CI 3.90-12.65), and physical neglect and self-harm (aRRR 3.81, 95% CI 1.77-8.21) and Co-occurring self-harm and depression (aRRR 6.16, 95% CI 3.21-11.85), were in line with those reported prospectively, albeit with larger effect sizes. There was an increased risk for depression among those that retrospectively reported emotional abuse and neglect (aRRR 2.62, 95% CI 1.85-3.71), however no association was found among those that prospectively reported emotional abuse and neglect, and the pattern for self-harm (aRRR 3.67, 95% CI 2.41-5.59) and co-occurring self-harm and depression (aRRR 7.65, 95% CI 5.27-11.12) was similar to that seen among the prospective measures. Separate measures of emotional abuse (self-harm: aRRR 3.69, 95% CI 2.30-5.91; depression: aRRR 3.06, 95% CI 2.06-4.54; both: aRRR 8.17, 95% CI 5.55-12.04) and neglect (self-harm: aRRR 2.69, 95% CI 1.59-4.55; depression: aRRR 1.73, 95% CI 1.09-2.75; both: aRRR 7.28, 95% CI 4.72-11.22) reported retrospectively were also associated with all three outcomes – these could not be disaggregated among the prospective measures. However, due to small numbers for both measures, the confidence intervals were wide. Retrospectively measured sexual abuse was associated with an increased risk of self-harm (aRRR 4.64, 95% CI 1.34-16.02) and co-occurring self-harm and depression (aRRR 16.09, 95% CI 6.27-41.25) – unlike prospectively collected reports of sexual abuse, which was associated with depression alone, and co-occurring self-harm and depression, but not self-harm alone.

Appendix S9: MCS complete-case sensitivity analyses

Table S14a shows crude and adjusted relative risk ratios (aRRR) for the association between individual ACEs and self-harm and depression in the MCS complete-case data.

*Self-harm alone*

Results were consistent with the MCS multiply imputed data for the association between substance abuse, and bullying and self-harm. In contrast to the multiply imputed data, no associations were found for parental mental health problems, domestic violence, and separation/divorce among the complete-cases.

*Depression alone*

Findings were consistent with the multiply imputed data for parental mental health problems, bullying, and separation/divorce and depression among the complete-cases, although the evidence of an association between separation/divorce and depression was weak among the complete-cases. No associations were found for domestic violence among the complete-cases, although the evidence in the multiply imputed data was weak.

*Co-occurring self-harm and depression*

The association between parental mental health problems, and bullying and co-occurring self-harm and depression was consistent with the multiply imputed data. In contrast to the multiply imputed data, no associations were found for domestic violence, substance abuse, and separation/divorce and co-occurring self-harm and depression among the complete-case data.

Table S14a: Crude and adjusted relative risk ratios for the association between adverse childhood experiences and self-harm and depression in MCS complete-cases

|  | Neither self-harm or depression n= 3,673 | Self-harm alone n= 306 | Depression alone n= 304 | Co-occurring self-harm and depression n= 366 | Neither self-harm or depression n= 3,673 | Self-harm alone n= 306 | Depression alone n= 304 | Co-occurring self-harm and depression n= 366 |
| --- | --- | --- | --- | --- | --- | --- | --- | --- |
|  | Model 1: unadjusted | | | | Model 2: adjusted | | | |
|  | Reference group | RRR (95% CI) | RRR (95% CI) | RRR (95% CI) | Reference group | RRR (95% CI) | RRR (95% CI) | RRR (95% CI) |
| Parental mental health problems | - | 1.11 (0.83-1.49) | 1.48 (1.12-1.96) | 1.50 (1.16-1.94) | - | 1.10 (0.81-1.50 | 1.44 (1.08-1.92) | 1.41 (1.08-1.85) |
| Domestic violence | - | 1.25 (0.91-1.71) | 1.14 (0.79-1.64) | 1.13 (0.84-1.50) | - | 1.25 (0.91-1.72) | 1.13 (0.79-1.63) | 1.12 (0.82-1.53) |
| Substance abuse | - | 1.40 (1.11-1.77) | 1.18 (0.89-1.56) | 1.09 (0.82-1.46) | - | 1.44 (1.13-1.83) | 1.23 (0.92-1.64) | 1.20 (0.89-1.60) |
| Bullying | - | 1.58 (1.18-2.10) | 1.41 (1.07-1.86) | 1.32 (1.03-1.70) | - | 1.68 (1.27-2.23) | 1.46 (1.10-1.94) | 1.45 (1.12-1.89) |
| Physical abuse | - | 0.86 (0.62-1.20) | 1.14 (0.83-1.56) | 0.97 (0.71-1.33) | - | 0.94 (0.68-1.31) | 1.23 (0.90-1.69) | 1.15 (0.83-1.59) |
| Separation/divorce | - | 1.19 (0.81-1.76) | 1.41 (0.98-2.03) | 1.11 (0.79-1.56) | - | 1.21 (0.82-1.77) | 1.41 (0.98-2.04) | 1.10 (0.77-1.57) |
| Model 2: adjusted for sex, child and mother’s ethnicity, mother’s age at delivery, mother’s education, household income, family housing tenure | | | | | | | | |

*Developmental timing and duration of exposure – MCS complete-cases*

Table S14b shows the first hypothesis selected by the Least Absolute Shrinkage and Selection Operator (lasso) that best supported the MCS complete-case data, separately for each outcome measure, and the associated effect estimates. The accumulation of risk, early, and middle childhood, and adolescence periods best supported the observed data.

*Early childhood*

For depression and co-occurring self-harm and depression, consistent with the MCS multiply imputed data, exposure to substance abuse (depression: OR 1.21, 99% CI 0.95-1.54; both: OR 1.29, 99% CI 0.95-1.76) in early childhood best supported the complete-case data. However, the confidence intervals included the null. Results were also not consistent with the ALSPAC complete-case data, where accumulation and middle childhood best supported the data.

*Middle childhood*

For the outcomes self-harm and co-occurring self-harm and depression, exposure to domestic violence (self-harm: OR 1.47, 99% CI 1.02-2.12; both: OR 1.64, 99% CI 1.04-2.59) in middle childhood best supported the complete-case data. However in the MCS multiply imputed data, accumulation of exposure was the best-fitting hypothesis, and in the ALSPAC complete-case data, early childhood exposure best supported the data.

*Early adolescence*

Consistent with results from the MCS imputed data, exposure to separation/divorce (self-harm: OR 1.19, 99% CI 0.72-1.96; depression: OR 1.23, 99% CI 0.75-2.02; both: OR 1.24, 99% CI 0.66-2.35) and bullying (self-harm: OR 1.66, 99% CI 1.25-2.21; depression: OR 1.77, 99% CI 1.33-2.35; both: OR 1.65, 99% CI 1.14-2.38) during early adolescence best supported the data for their association with all three outcomes. However, among the complete-cases the confidence intervals for separation/divorce included the null. Among the ALSPAC complete-case sample, early childhood exposure to separation/divorce best supported the data for all three outcomes. Bullying was not examined in ALSPAC as measures were not available for all three critical periods.

*Accumulation of risk*

For all three outcomes, consistent with results from the MCS multiply imputed data, cumulative exposure to parental mental health problems best supported the complete-case data (self-harm: OR 1.11, 99% CI 0.99-1.25; depression: OR 1.19, 99% CI 1.06-1.33; both: OR 1.17, 99% CI 1.01-1.36). However middle and early childhood best supported the ALSPAC complete-case data.

For self-harm, accumulation of exposure to substance abuse, as opposed to exposure during early childhood in the multiply imputed data, best supported the MCS complete-case data (OR 1.14, 99% CI 1.03-1.26). This was also consistent with the ALSPAC complete-case results.

For the outcome depression, consistent with the MCS multiply imputed data, accumulation of exposure to domestic violence best supported the complete-case data (OR 1.15, 99% CI 1.00-1.32). However, exposure during early childhood best supported the ALSPAC complete-case data.

Table S14b: Adjusted odds ratios and 99% confidence intervals, for the association between the first life course hypothesis selected by the Least Absolute Shrinkage and Selection Operator (lasso) and each outcome measure among the MCS complete-cases.

|  | Self-harm | | | Depression | | | Co-occurring self-harm and depression | | |
| --- | --- | --- | --- | --- | --- | --- | --- | --- | --- |
| Adverse childhood experience | Hypothesis | OR (99% CI) | P-value* | Hypothesis | OR (99% CI) | P-value* | Hypothesis | OR (99% CI) | P-value* |
| Parental mental health problems | Accumulation | 1.11 (0.99-1.25) | 0.065 | Accumulation | 1.19 (1.06-1.33) | 0.000 | Accumulation | 1.17 (1.01-1.36) | 0.021 |
| Domestic violence | Middle childhood | 1.47 (1.02-2.12) | 0.026 | Accumulation | 1.15 (1.00-1.32) | 0.045 | Middle childhood | 1.64 (1.04-2.59) | 0.020 |
| Substance abuse | Accumulation | 1.14 (1.03-1.26) | 0.005 | Early childhood | 1.21 (0.95-1.54) | 0.178 | Early childhood | 1.29 (0.95-1.76) | 0.134 |
| Separation/divorce | Early adolescence | 1.19 (0.72-1.96) | 1.00 | Early adolescence | 1.23 (0.75-2.02) | 1.00 | Early adolescence | 1.24 (0.66-2.35) | 1.00 |
| Bullying | Early adolescence | 1.66 (1.25-2.21) | 0.000 | Early adolescence | 1.77 (1.33-2.35) | 0.000 | Early adolescence | 1.65 (1.14-2.38) | 0.002 |
| *Bonferroni correction applied  OR = Odds Ratio; 99% CI = 99% Confidence Interval. | | | | | | | | | |

Appendix S10: SLCMA study code

*## SLCMA script for stacked multiply imputed data with Bonferroni correction ##*

*# Load libraries*

*library(glmnet)*

*# This setting helps behaviour*

*glmnet.control(fdev = 0)*

*### Function for lasso logistic regression*

*### based on glmnet*

*### but wrapped as if from lars*

*lars.glmnet.binomial <- function(x, y, lambda.min.ratio = ifelse(nobs < nvars, 0.01, 1e-04), ...) {*

*x <- scale(x,TRUE,FALSE)*

*sx <- sqrt(colSums(x^2))*

*x <- scale(x,FALSE,sx)*

*nobs <- nrow(x)*

*nvars <- ncol(x)*

*fit1 <- glmnet(x, y, family="binomial",*

*alpha = 1, lambda.min.ratio=lambda.min.ratio, lambda=NULL, standardize=FALSE, intercept=TRUE , ...)*

*nl <- length(fit1$lambda)*

*sign.beta <- sign(fit1$beta)*

*change.after <- which(apply(sign.beta[,-1] != sign.beta[,-nl],2,any))*

*starts <- fit1$lambda[change.after]*

*stops <- fit1$lambda[change.after+1]*

*dl <- fit1$lambda[1]*lambda.min.ratio*

*lambda.grid <- unlist(mapply(seq, starts, stops, -dl))*

*lambda.grid <- unique(rev(sort(c(fit1$lambda, lambda.grid))))*

*fit2 <- glmnet(x, y, family="binomial",*

*alpha = 1, lambda.min.ratio=lambda.min.ratio, lambda=lambda.grid, standardize=FALSE, intercept=TRUE , ...)*

*beta.grid <- fit2$beta*

*beta.grid[,1] <- fit1$beta[,1]*

*ng <- length(lambda.grid)*

*sign.beta <- sign(beta.grid)*

*change.after <- which(apply(sign.beta[,-1] != sign.beta[,-ng],2,any))*

*lambda <- numeric(0)*

*action <- numeric(0)*

*a0 <- numeric(0)*

*beta <- beta.grid[,0]*

*for(ch in change.after) {*

*vars <- which(sign.beta[,ch] != sign.beta[,ch+1])*

*for(v in vars) {*

*if(sign.beta[v,ch] != 0 & sign.beta[v,ch+1] == 0) {*

*lambda <- c(lambda, lambda.grid[ch+1]);*

*action <- c(action, -v)*

*a0 <- c(a0, fit2$a0[ch+1])*

*beta <- cbind(beta, beta.grid[,ch+1])*

*}*

*}*

*for(v in vars) {*

*if(sign.beta[v,ch] == 0 & sign.beta[v,ch+1] != 0) {*

*lambda <- c(lambda, lambda.grid[ch])*

*action <- c(action, v)*

*a0 <- c(a0, fit2$a0[ch])*

*beta <- cbind(beta, beta.grid[,ch])*

*}*

*}*

*}*

*lambda <- c(lambda, fit1$lambda[nl])*

*action <- c(action, 0)*

*a0 <- c(a0, fit1$a0[nl])*

*beta <- cbind(beta, beta.grid[,ng])*

*while(any(diff(lambda)==0)) {*

*dups <- which(diff(lambda)==0)*

*l <- dups[length(dups)] + 1*

*lambda[l] <- lambda[l]-0.5*dl*

*v <- action[l-1]*

*if(v > 0) {*

*beta[v,l] <- 2e-05*

*}*

*if(v < 0) {*

*beta[-v,l-1] <- 2e-05*

*}*

*}*

*list(call = match.call(), family="binomial", action=as.list(action), lambda=lambda*nobs, standardize=FALSE,*

*beta = t(beta), a0 = a0, scale=attributes(x)$`scaled:scale`, x=x, y=y)*

*}*

*source("Binomial lasso functions 2022.R")*

*library(mice)*

*library(boot)*

*library(glmnet)*

*########## ALSPAC data #############*

*### Load stacked dataset*

*library(foreign)*

*stackedData <- read.dta ('final_imp_alspac.dta')*

*m <- 50*

*# Size of each imputed dataset*

*n <- 8360*

*# Encode hypotheses*

*# Parental mental health problems*

*criticalmh_1 <- stackedData$mentalhealthprob5*

*criticalmh_2 <- stackedData$mentalhealthprob10*

*criticalmh_3 <- stackedData$mentalhealthprob13*

*accummh <- stackedData$mentalhealthprob5 + stackedData$mentalhealthprob10 + stackedData$mentalhealthprob13*

*# Domestic violence*

*criticaldv_1 <- stackedData$domesticviol5*

*criticaldv_2 <- stackedData$domesticviol10*

*criticaldv_3 <- stackedData$domesticviol13*

*accumdv <- stackedData$domesticviol5 + stackedData$domesticviol10 + stackedData$domesticviol13*

*# Separation/divorce*

*criticaldiv_1 <- stackedData$separation5*

*criticaldiv_2 <- stackedData$separation10*

*criticaldiv_3 <- stackedData$separation13*

*accumdiv <- stackedData$separation5 + stackedData$separation10 + stackedData$separation13*

*# Physical abuse*

*criticalpa_1 <- stackedData$physicalabuse5*

*criticalpa_2 <- stackedData$physicalabuse10*

*criticalpa_3 <- stackedData$physicalabuse13*

*accumpa <- stackedData$physicalabuse5 + stackedData$physicalabuse10 + stackedData$physicalabuse13*

*# Emotional abuse*

*criticalea_1 <- stackedData$emotionalabus5*

*criticalea_2 <- stackedData$emotionalabus10*

*criticalea_3 <- stackedData$emotionalabus13*

*accumea <- stackedData$emotionalabus5 + stackedData$emotionalabus10 + stackedData$emotionalabus13*

*# Parental conviction*

*criticalconv_1 <- stackedData$parentconvct5*

*criticalconv_2 <- stackedData$parentconvct10*

*criticalconv_3 <- stackedData$parentconvct13*

*accumconv <- stackedData$parentconvct5 + stackedData$parentconvct10 + stackedData$parentconvct13*

*# Encode covariates: mother’s age at delivery, educational qualifications, social class, housing tenure, and parity*

*mage <- stackedData$mage*

*mqual <- stackedData$mqual*

*socialc <- stackedData$socialclass18w*

*housing <- stackedData$housing*

*parity <- stackedData$parity*

*n_covars <- 5*

*n_hypos <- 4*

*X_stackedmh <- cbind(mage, mqual, socialc, housing, parity,*

*criticalmh_1, criticalmh_2, criticalmh_3, accummh)*

*X_stackeddv <- cbind(mage, mqual, socialc, housing, parity,*

*criticaldv_1, criticaldv_2, criticaldv_3, accumdv)*

*X_stackeddiv <- cbind(mage, mqual, socialc, housing, parity,*

*criticaldiv_1, criticaldiv_2, criticaldiv_3, accumdiv)*

*X_stackedpa <- cbind(mage, mqual, socialc, housing, parity,*

*criticalpa_1, criticalpa_2, criticalpa_3, accumpa)*

*X_stackedea <- cbind(mage, mqual, socialc, housing, parity,*

*criticalea_1, criticalea_2, criticalea_3, accumea)*

*X_stackedconv <- cbind(mage, mqual, socialc, housing, parity,*

*criticalconv_1, criticalconv_2, criticalconv_3, accumconv)*

*# Outcomes*

*y_stackedsh <- stackedData$selfharm_16_12m_org*

*y_stackeddepr <- stackedData$depression16_org*

*y_stackedboth <- stackedData$shdepression*

*# Apply lars.glmnet.binomial*

*penalty <- c(rep(0,n_covars),rep(1,n_hypos))*

*require(glmnet)*

*# parental mental health problems and self-harm*

*lasso_stackedmhsh <- lars.glmnet.binomial(X_stackedmh, y_stackedsh, penalty.factor=penalty, weights=rep(1/m, m*n))*

*# parental mental health problems and depression*

*lasso_stackedmhdep <- lars.glmnet.binomial(X_stackedmh, y_stackeddepr, penalty.factor=penalty, weights=rep(1/m, m*n))*

*# parental mental health problems and both self-harm and depression*

*lasso_stackedboth1 <- lars.glmnet.binomial(X_stackedmh, y_stackedboth, penalty.factor=penalty, weights=rep(1/m, m*n))*

*# domestic violence and self-harm*

*lasso_stackeddvsh <- lars.glmnet.binomial(X_stackeddv, y_stackedsh, penalty.factor=penalty, weights=rep(1/m, m*n))*

*# domestic violence and depression*

*lasso_stackeddvdep <- lars.glmnet.binomial(X_stackeddv, y_stackeddepr, penalty.factor=penalty, weights=rep(1/m, m*n))*

*# domestic violence and both self-harm and depression*

*lasso_stackedboth2 <- lars.glmnet.binomial(X_stackeddv, y_stackedboth, penalty.factor=penalty, weights=rep(1/m, m*n))*

*# separation/divorce and self-harm*

*lasso_stackeddivsh <- lars.glmnet.binomial(X_stackeddiv, y_stackedsh, penalty.factor=penalty, weights=rep(1/m, m*n))*

*# separation/divorce and depression*

*lasso_stackeddivdep <- lars.glmnet.binomial(X_stackeddiv, y_stackeddepr, penalty.factor=penalty, weights=rep(1/m, m*n))*

*# separation/divorce and both self-harm and depression*

*lasso_stackedboth3 <- lars.glmnet.binomial(X_stackeddiv, y_stackedboth, penalty.factor=penalty, weights=rep(1/m, m*n))*

*# physical abuse and self-harm*

*lasso_stackedpash <- lars.glmnet.binomial(X_stackedpa, y_stackedsh, penalty.factor=penalty, weights=rep(1/m, m*n))*

*# separation/divorce and depression*

*lasso_stackedpadep <- lars.glmnet.binomial(X_stackedpa, y_stackeddepr, penalty.factor=penalty, weights=rep(1/m, m*n))*

*# separation/divorce and both self-harm and depression*

*lasso_stackedboth4 <- lars.glmnet.binomial(X_stackedpa, y_stackedboth, penalty.factor=penalty, weights=rep(1/m, m*n))*

*# emotional abuse and self-harm*

*lasso_stackedeash <- lars.glmnet.binomial(X_stackedea, y_stackedsh, penalty.factor=penalty, weights=rep(1/m, m*n))*

*# separation/divorce and depression*

*lasso_stackedeadep <- lars.glmnet.binomial(X_stackedea, y_stackeddepr, penalty.factor=penalty, weights=rep(1/m, m*n))*

*# separation/divorce and both self-harm and depression*

*lasso_stackedboth5 <- lars.glmnet.binomial(X_stackedea, y_stackedboth, penalty.factor=penalty, weights=rep(1/m, m*n))*

*# parent convicted and self-harm*

*lasso_stackedconvsh <- lars.glmnet.binomial(X_stackedconv, y_stackedsh, penalty.factor=penalty, weights=rep(1/m, m*n))*

*# separation/divorce and depression*

*lasso_stackedconvdep <- lars.glmnet.binomial(X_stackedconv, y_stackeddepr, penalty.factor=penalty, weights=rep(1/m, m*n))*

*# separation/divorce and both self-harm and depression*

*lasso_stackedboth6 <- lars.glmnet.binomial(X_stackedconv, y_stackedboth, penalty.factor=penalty, weights=rep(1/m, m*n))*

*# Elbow plot using McFadden's psuedo-R2*

*elbow(lasso_stackedmhsh) # mh problems and self-harm*

*elbow(lasso_stackedmhdep) # mh problems and depression*

*elbow(lasso_stackedboth1) # mh problems and both self-harm and depression*

*elbow(lasso_stackeddvsh) # domestic violence and self-harm*

*elbow(lasso_stackeddvdep) # domestic violence and depression*

*elbow(lasso_stackedboth2) # domestic violence and both self-harm and depression*

*elbow(lasso_stackeddivsh) # sep/div and self-harm*

*elbow(lasso_stackeddivdep) # sep/div and depression*

*elbow(lasso_stackedboth3) # sep/div and both self-harm and depression*

*elbow(lasso_stackedpash) # physical abuse and self-harm*

*elbow(lasso_stackedpadep) # physical abuse and depression*

*elbow(lasso_stackedboth4) # physical abuse and both self-harm and depression*

*elbow(lasso_stackedeash) # emotional abuse and self-harm*

*elbow(lasso_stackedeadep) # emotional abuse and depression*

*elbow(lasso_stackedboth5) # emotional abuse and both self-harm and depression*

*elbow(lasso_stackedconvsh) # parent conv and self-harm*

*elbow(lasso_stackedconvdep) # parent conv and depression*

*elbow(lasso_stackedboth6) # parent conv and both self-harm and depression*

*# Function that gets coefficients and applies Bonferroni correction*

*# (Bonferroni correction is a conservative approach, it means multiplying p-values by n_hypos)*

*stackedcoef <- function(lgbobj, actnum, weights, n_hypos, alpha=0.05) {*

*beta <- lgbobj$beta[actnum,]*

*se <- beta*

*pval <- beta*

*positions <- beta != 0*

*coeftable <- summary(glm(lgbobj$y ~ lgbobj$x[,positions], family="binomial", weights=weights))$coef*

*beta[positions] <- coeftable[,1][-1]*

*beta <- beta / lgbobj$scale*

*se[positions] <- coeftable[,2][-1]*

*se <- se / lgbobj$scale*

*pval[positions] <- coeftable[,4][-1]*

*pval <- pval*n_hypos*

*pval[pval>1] <- 1*

*lower <- beta + qnorm(alpha/2)*se*

*upper <- beta + qnorm(1-alpha/2)*se*

*round(cbind(beta, se, lower, upper, pval)[positions,],3)*

*}*

*# Ignore warnings about 'non-integer #successes'*

*## Parental mental health problems & self-harm*

*stackedcoef(lasso_stackedmhsh, 2, weights=rep(1/m, m*n), n_hypos=n_hypos)# first variable plus covariates*

*#stackedcoef(lasso_stackedmhsh, 3, weights=rep(1/m, m*n), n_hypos=n_hypos) # first two variables plus covariates*

*#stackedcoef(lasso_stackedmhsh, 4, weights=rep(1/m, m*n), n_hypos=n_hypos) # first three variables plus covariates*

*## Parental mental health problems & depression*

*stackedcoef(lasso_stackedmhdep, 2, weights=rep(1/m, m*n), n_hypos=n_hypos)# first variable plus covariates*

*#stackedcoef(lasso_stackedmhdep, 3, weights=rep(1/m, m*n), n_hypos=n_hypos) # first two variables plus covariates*

*#stackedcoef(lasso_stackedmhdep, 4, weights=rep(1/m, m*n), n_hypos=n_hypos) # first three variables plus covariates*

*## Parental mental health problems and both sh & depression*

*stackedcoef(lasso_stackedboth1, 2, weights=rep(1/m, m*n), n_hypos=n_hypos)# first variable plus covariates*

*#stackedcoef(lasso_stackedboth1, 3, weights=rep(1/m, m*n), n_hypos=n_hypos) # first two variables plus covariates*

*#stackedcoef(lasso_stackedboth1, 4, weights=rep(1/m, m*n), n_hypos=n_hypos) # first three variables plus covariates*

*## Domestic violence & self-harm*

*stackedcoef(lasso_stackeddvsh, 2, weights=rep(1/m, m*n), n_hypos=n_hypos)# first variable plus covariates*

*#stackedcoef(lasso_stackeddvsh, 3, weights=rep(1/m, m*n), n_hypos=n_hypos) # first two variables plus covariates*

*#stackedcoef(lasso_stackeddvsh, 4, weights=rep(1/m, m*n), n_hypos=n_hypos) # first three variables plus covariates*

*## Domestic violence & depression*

*stackedcoef(lasso_stackeddvdep, 2, weights=rep(1/m, m*n), n_hypos=n_hypos)# first variable plus covariates*

*#stackedcoef(lasso_stackeddvdep, 3, weights=rep(1/m, m*n), n_hypos=n_hypos) # first two variables plus covariates*

*#stackedcoef(lasso_stackeddvdep, 4, weights=rep(1/m, m*n), n_hypos=n_hypos) # first three variables plus covariates*

*## Domestic violence and both self-harm & depression*

*stackedcoef(lasso_stackedboth2, 2, weights=rep(1/m, m*n), n_hypos=n_hypos)# first variable plus covariates*

*#stackedcoef(lasso_stackedboth2, 3, weights=rep(1/m, m*n), n_hypos=n_hypos) # first two variables plus covariates*

*#stackedcoef(lasso_stackedboth2, 4, weights=rep(1/m, m*n), n_hypos=n_hypos) # first three variables plus covariates*

*## Physical abuse & self-harm*

*stackedcoef(lasso_stackedpash, 2, weights=rep(1/m, m*n), n_hypos=n_hypos)# first variable plus covariates*

*#stackedcoef(lasso_stackedpash, 3, weights=rep(1/m, m*n), n_hypos=n_hypos) # first two variables plus covariates*

*#stackedcoef(lasso_stackedpash, 4, weights=rep(1/m, m*n), n_hypos=n_hypos) # first three variables plus covariates*

*## Physical abuse & depression*

*stackedcoef(lasso_stackedpadep, 2, weights=rep(1/m, m*n), n_hypos=n_hypos)# first variable plus covariates*

*#stackedcoef(lasso_stackedpadep, 3, weights=rep(1/m, m*n), n_hypos=n_hypos) # first two variables plus covariates*

*#stackedcoef(lasso_stackedpadep, 4, weights=rep(1/m, m*n), n_hypos=n_hypos) # first three variables plus covariates*

*## Physical abuse and both self-harm & depression*

*stackedcoef(lasso_stackedboth4, 2, weights=rep(1/m, m*n), n_hypos=n_hypos)# first variable plus covariates*

*#stackedcoef(lasso_stackedboth4, 3, weights=rep(1/m, m*n), n_hypos=n_hypos) # first two variables plus covariates*

*#stackedcoef(lasso_stackedboth4, 4, weights=rep(1/m, m*n), n_hypos=n_hypos) # first three variables plus covariates*

*## Emotional abuse & self-harm*

*stackedcoef(lasso_stackedeash, 2, weights=rep(1/m, m*n), n_hypos=n_hypos)# first variable plus covariates*

*#stackedcoef(lasso_stackedeash, 3, weights=rep(1/m, m*n), n_hypos=n_hypos) # first two variables plus covariates*

*#stackedcoef(lasso_stackedeash, 4, weights=rep(1/m, m*n), n_hypos=n_hypos) # first three variables plus covariates*

*## Emotional abuse & depression*

*stackedcoef(lasso_stackedeadep, 2, weights=rep(1/m, m*n), n_hypos=n_hypos)# first variable plus covariates*

*#stackedcoef(lasso_stackedeadep, 3, weights=rep(1/m, m*n), n_hypos=n_hypos) # first two variables plus covariates*

*#stackedcoef(lasso_stackedeadep, 4, weights=rep(1/m, m*n), n_hypos=n_hypos) # first three variables plus covariates*

*## Emotional abuse and both sh & depression*

*stackedcoef(lasso_stackedboth5, 2, weights=rep(1/m, m*n), n_hypos=n_hypos)# first variable plus covariates*

*#stackedcoef(lasso_stackedboth5, 3, weights=rep(1/m, m*n), n_hypos=n_hypos) # first two variables plus covariates*

*#stackedcoef(lasso_stackedboth5, 4, weights=rep(1/m, m*n), n_hypos=n_hypos) # first three variables plus covariates*

*## Separation/divorce & sh*

*stackedcoef(lasso_stackeddivsh, 2, weights=rep(1/m, m*n), n_hypos=n_hypos)# first variable plus covariates*

*#stackedcoef(lasso_stackeddivsh, 3, weights=rep(1/m, m*n), n_hypos=n_hypos) # first two variables plus covariates*

*#stackedcoef(lasso_stackeddivsh, 4, weights=rep(1/m, m*n), n_hypos=n_hypos) # first three variables plus covariates*

*## Separation/divorce & depression*

*stackedcoef(lasso_stackeddivdep, 2, weights=rep(1/m, m*n), n_hypos=n_hypos)# first variable plus covariates*

*#stackedcoef(lasso_stackeddivdep, 3, weights=rep(1/m, m*n), n_hypos=n_hypos) # first two variables plus covariates*

*#stackedcoef(lasso_stackeddivdep, 4, weights=rep(1/m, m*n), n_hypos=n_hypos) # first three variables plus covariates*

*## Separation/divorce and both sh & depression*

*stackedcoef(lasso_stackedboth3, 2, weights=rep(1/m, m*n), n_hypos=n_hypos)# first variable plus covariates*

*#stackedcoef(lasso_stackedboth3, 3, weights=rep(1/m, m*n), n_hypos=n_hypos) # first two variables plus covariates*

*#stackedcoef(lasso_stackedboth3, 4, weights=rep(1/m, m*n), n_hypos=n_hypos) # first three variables plus covariates*

*## Parent conviction & sh*

*stackedcoef(lasso_stackedconvsh, 2, weights=rep(1/m, m*n), n_hypos=n_hypos)# first variable plus covariates*

*#stackedcoef(lasso_stackedconvsh, 3, weights=rep(1/m, m*n), n_hypos=n_hypos) # first two variables plus covariates*

*#stackedcoef(lasso_stackedconvsh, 4, weights=rep(1/m, m*n), n_hypos=n_hypos) # first three variables plus covariates*

*## Parent conviction & depression*

*stackedcoef(lasso_stackedconvdep, 2, weights=rep(1/m, m*n), n_hypos=n_hypos)# first variable plus covariates*

*#stackedcoef(lasso_stackedconvdep, 3, weights=rep(1/m, m*n), n_hypos=n_hypos) # first two variables plus covariates*

*#stackedcoef(lasso_stackedconvdep, 4, weights=rep(1/m, m*n), n_hypos=n_hypos) # first three variables plus covariates*

*## Parent conviction and both sh & depression*

*stackedcoef(lasso_stackedboth6, 2, weights=rep(1/m, m*n), n_hypos=n_hypos)# first variable plus covariates*

*#stackedcoef(lasso_stackedboth6, 3, weights=rep(1/m, m*n), n_hypos=n_hypos) # first two variables plus covariates*

*#stackedcoef(lasso_stackedboth6, 4, weights=rep(1/m, m*n), n_hypos=n_hypos) # first three variables plus covariates*

References

Azur, M. J., Stuart, E. A., Frangakis, C., & Leaf, P. J. (2011). Multiple imputation by chained equations: what is it and how does it work? *International Journal of Methods in Psychiatric Research, 20*(1), 40-49.

Bernstein, D. P., Fink, L., Handelsman, L., Foote, J., Lovejoy, M., Wenzel, K., et al. (1994). Initial reliability and validity of a new retrospective measure of child abuse and neglect. *The American Journal of Psychiatry, 151*(8), 1132-1136.

Bernstein, D. P., Stein, J. A., Newcomb, M. D., Walker, E., Pogge, D., Ahluvalia, T., et al. (2003). Development and validation of a brief screening version of the Childhood Trauma Questionnaire. *Child Abuse & Neglect, 27*(2), 169-190.

Boyd, A., Golding, J., Macleod, J., Lawlor, D. A., Fraser, A., Henderson, J., et al. (2013). Cohort Profile: the 'children of the 90s'--the index offspring of the Avon Longitudinal Study of Parents and Children. *International Journal of Epidemiology, 42*(1), 111-127.

Bradley, R. H., & Caldwell, B. M. (1977). Home observation for measurement of the environment: a validation study of screening efficiency. *American Journal of Mental Deficiency, 81*(5), 417-420.

Centre for Longitudinal Studies. (2015). *Millennium Cohort Study: Psychological, Developmental and Health Inventories*. London Centre for Longitudinal Studies, Institute of Education.

Danese, A., Moffitt, T. E., Arseneault, L., Bleiberg, B. A., Dinardo, P. B., Gandelman, S. B., et al. (2017). The Origins of Cognitive Deficits in Victimized Children: Implications for Neuroscientists and Clinicians. *The American Journal of Psychiatry, 174*(4), 349-361.

Fraser, A., Macdonald-Wallis, C., Tilling, K., Boyd, A., Golding, J., Davey Smith, G., et al. (2013). Cohort Profile: the Avon Longitudinal Study of Parents and Children: ALSPAC mothers cohort. *International Journal of Epidemiology, 42*(1), 97-110.

Houtepen, L. C., Heron, J., Suderman, M. J., Tilling, K., & Howe, L. D. (2018). Adverse childhood experiences in the children of the Avon Longitudinal Study of Parents and Children (ALSPAC). *Wellcome Open Research, 3*, 106.

Kessler, R. C., Andrews, G., Colpe, L. J., Hiripi, E., Mroczek, D. K., Normand, S. L., et al. (2002). Short screening scales to monitor population prevalences and trends in non-specific psychological distress. *Psychological Medicine, 32*(6), 959-976.

Lansford, J. E., Dodge, K. A., Pettit, G. S., Bates, J. E., Crozier, J., & Kaplow, J. (2002). A 12-year prospective study of the long-term effects of early child physical maltreatment on psychological, behavioral, and academic problems in adolescence. *Archives of Pediatrics and Adolescent Medicine, 156*(8), 824-830.

Mensah, F. K., & Kiernan, K. E. (2010). Parents' mental health and children's cognitive and social development: families in England in the Millennium Cohort Study. *Social Psychiatry and Psychiatric Epidemiology, 45*(11), 1023-1035.

Moore, L., Jayaweera, H., Redshaw, M., & Quigley, M. (2019). Migration, ethnicity and mental health: evidence from mothers participating in the Millennium Cohort Study. *Public Health, 171*, 66-75.

Northstone, K., Ben Shlomo, Y., Teyhan, A., Hill, A., Groom, A., Mumme, M., et al. (2023). The Avon Longitudinal Study of Parents and children ALSPAC G0 Partners: A cohort profile [version 1; peer review: 1 approved with reservations]. *Wellcome Open Research, 8*(37).

Scher, C. D., Stein, M. B., Asmundson, G. J., McCreary, D. R., & Forde, D. R. (2001). The childhood trauma questionnaire in a community sample: psychometric properties and normative data. *Journal of Traumatic Stress, 14*(4), 843-857.

Straus, M. A. (1979). Measuring Intrafamily Conflict and Violence: The Conflict Tactics (CT) Scales. *Journal of Marriage and Family, 41*(1), 75-88.

Weissman, M. M., Wickramaratne, P., Adams, P., Wolk, S., Verdeli, H., & Olfson, M. (2000). Brief Screening for Family Psychiatric History: The Family History Screen. *Archives of General Psychiatry, 57*(7), 675-682.

White, I. R., Royston, P., & Wood, A. M. (2011). Multiple imputation using chained equations: Issues and guidance for practice. *Statistics in Medicine, 30*(4), 377-399.
